# A highly resolved food web for insect seed predators in a species‐rich tropical forest

**DOI:** 10.1111/ele.13359

**Published:** 2019-07-29

**Authors:** Sofia Gripenberg, Yves Basset, Owen T. Lewis, J. Christopher D. Terry, S. Joseph Wright, Indira Simón, D. Catalina Fernández, Marjorie Cedeño‐Sanchez, Marleny Rivera, Héctor Barrios, John W. Brown, Osvaldo Calderón, Anthony I. Cognato, Jorma Kim, Scott E. Miller, Geoffrey E. Morse, Sara Pinzón‐Navarro, Donald L. J. Quicke, Robert K. Robbins, Juha‐Pekka Salminen, Eero Vesterinen

**Affiliations:** ^1^ School of Biological Sciences University of Reading Reading UK; ^2^ Smithsonian Tropical Research Institute Balboa Republic of Panama; ^3^ Department of Zoology University of Oxford Oxford UK; ^4^ Biodiversity Unit University of Turku Turku Finland; ^5^ ForestGEO Smithsonian Tropical Research Institute Balboa Republic of Panama; ^6^ Faculty of Science University of South Bohemia Ceske Budejovice Czech Republic; ^7^ Biology Centre of the Czech Academy of Sciences Institute of Entomology Ceske Budejovice Czech Republic; ^8^ Maestria de Entomologia Universidad de Panamá Panama Republic of Panama; ^9^ National Museum of Natural History Smithsonian Institution Washington, DC USA; ^10^ Department of Entomology Michigan State University East Lansing MI USA; ^11^ Department of Chemistry University of Turku Turku Finland; ^12^ Biology Department University of San Diego San Diego CA USA; ^13^ Integrative Ecology Laboratory, Department of Biology, Faculty of Science Chulalongkorn University Bangkok Thailand; ^14^ Department of Ecology Swedish University of Agricultural Sciences Uppsala Sweden

**Keywords:** Apparent competition, Barro Colorado Island, host specialisation, interaction network, Janzen–Connell hypothesis, Panama, plant traits, quantitative food web, seed predation

## Abstract

The top‐down and indirect effects of insects on plant communities depend on patterns of host use, which are often poorly documented, particularly in species‐rich tropical forests. At Barro Colorado Island, Panama, we compiled the first food web quantifying trophic interactions between the majority of co‐occurring woody plant species and their internally feeding insect seed predators. Our study is based on more than 200 000 fruits representing 478 plant species, associated with 369 insect species. Insect host‐specificity was remarkably high: only 20% of seed predator species were associated with more than one plant species, while each tree species experienced seed predation from a median of two insect species. Phylogeny, but not plant traits, explained patterns of seed predator attack. These data suggest that seed predators are unlikely to mediate indirect interactions such as apparent competition between plant species, but are consistent with their proposed contribution to maintaining plant diversity via the Janzen–Connell mechanism.

## Introduction

Natural enemies of plants such as herbivores and pathogens are important determinants of the fitness of individual plants, the dynamics of plant populations, and the diversity and composition of entire plant communities (Crawley 1989; Burdon *et al.* 2006; Bradley *et al.* 2008; Bagchi *et al.* 2014). Plant‐enemy interactions are particularly frequent and intense in tropical forests (Schemske *et al.* 2009), where the diversity of both plants and enemies peaks (Willig *et al.* 2003). However, patterns of enemy attack across plant species, and patterns of host use by individual enemy species, are often particularly poorly known in tropical forests. As a result, these patterns are often inferred from other information. For example, closely related plant species are expected to be attacked by similar sets of enemies, leading to phylogenetic clustering of susceptibility and patterns of enemy attack (Gilbert & Webb 2007; Gilbert *et al.* 2015).

Plant species vary greatly in their susceptibility to natural enemies (Coley 1983; Fritz & Simms 1992; Marquis *et al.* 2001). Understanding the causes and consequences of this variation is a central aim for ecologists studying plant‐enemy interactions (e.g. Carmona *et al.* 2011; Loranger *et al.* 2012; Turcotte *et al.* 2014). A variety of traits and ecological circumstances have been hypothesised to drive interspecific variation in plant susceptibility to enemies. For example, plant species that invest heavily in defences are likely to be less prone to enemy attack and to have fewer and more specialised consumer species (Walters 2011), as are plant species with unpredictable distributions in space or time (Feeny 1976).

Most studies linking interspecific variation in enemy attack to plant traits have focused on leaf herbivory (e.g. Coley 1983; Schuldt *et al.* 2012; Cárdenas *et al.* 2014). However, leaves represent only one component of plant biomass. Traits influencing plant susceptibility to enemy attack will differ among plant tissues and organs (Sang *et al.* 1984; Brown *et al.* 2003), and may change during ontogeny (Boege & Marquis 2006) for example as a result of developmental changes in plant tolerance to enemy attack (Boege *et al.* 2007). Thus, new insights into the role of enemies in structuring plant communities could emerge from studying variation in the susceptibility of plants to enemies attacking non‐foliar plant tissues and different stages of the plant life cycle. Of particular interest are the seed and early seedling stages, which represent important demographic bottlenecks (e.g. Fenner & Thompson 2005; Leck *et al.* 2008; Green *et al.* 2014). Despite calls for more focus on the seed stage (e.g. Janzen 1969; Lewis & Gripenberg 2008), few studies have investigated plant susceptibility to seed enemies (but see e.g. Zalamea *et al.* 2018).

Information on the identity and specificity of plant enemies is also important for predicting their community‐level effects. Such effects have received particular attention in tropical forests, where plant enemies are thought to contribute to maintaining the high diversity of woody plant species. According to the Janzen–Connell hypothesis (Janzen 1970; Connell 1971), plant enemies can facilitate plant species coexistence and promote diversity, provided that these enemies are relatively host‐specific (Sedio & Ostling 2013). Existing data suggest that, while specificity varies markedly among different guilds and taxa, relatively few enemies are specialised on a single plant species (e.g. Novotny *et al.* 2002, 2010). Where enemies have host ranges spanning multiple species, they might instead contribute to eroding plant diversity (by excluding the species that are least tolerant to enemy attack), or to structuring the plant community through processes such as ‘apparent competition’ and ‘apparent mutualism’ (Holt 1977; Lewis & Gripenberg 2008). If enemies are specialised on several closely related plant species (e.g. Gilbert *et al.* 2012), we expect the potential for enemy‐mediated indirect interactions to depend on the degree of phylogenetic relatedness among plants. While enemy‐mediated indirect interactions have been documented in a number of systems, they have so far received little attention in the context of tropical forest plant communities (but see Garzon‐Lopez *et al.* 2015; Downey *et al.* 2018).

Obtaining detailed, multi‐species information on plant‐enemy interactions and their consequences can be challenging, particularly in species‐rich tropical forests. For example, although leaves that have experienced herbivory are often ubiquitous, herbivores are rarely observed in the process of feeding (Elton 1975). Moreover, even if host associations of plant enemies can be established, it can be difficult to quantify the fitness consequences for long‐lived plants. In this context, the internally feeding insect seed predators attacking developing and mature seeds offer a convenient and relevant study system. By killing seeds, either after they have been dispersed or while they are still attached to the mother plant, these insects have direct and measurable fitness consequences for their hosts (e.g. Crawley 2000; Kolb *et al.* 2007). In a classic paper, Janzen (1980) assessed patterns of host use by 110 species of beetles associated with seeds of 100 plant species in Guanacaste province, Costa Rica. His study showed that the specificity of beetles was high, consistent with a contribution to maintaining plant species diversity via the Janzen–Connell hypothesis.

In this study, we document a food web including the great majority of woody plants and their endophagous seed predators at our study site, Barro Colorado Island in Panama. Our principal aims are (1) to describe community‐level patterns of insect seed predator attack; (2) to assess the role of phylogeny and of a set of relevant plant traits (Table 1) in influencing how prone plant species are to seed predator attack; (3) to measure the degree of seed predator host‐specificity and the extent to which it varies among taxa; and (4) to test whether there is potential for enemy‐mediated interactions between plant species via shared seed predators.

**Table 1:** Traits hypothesised to influence plant species’ susceptibility to attack by internally‐feeding seed predators. In the context of our study, the proneness of plant species to seed predator attack was assessed as incidence of seed predators, seed predator richness (number of seed predator species observed on each plant species) and seed predation rates (assessed by seed dissection). For details on how individual variables were estimated, see Appendix S2

Variable	Predicted relationship
*Variables reflecting resource availability at various spatial scales*	
Local seed abundance	Species that are locally abundant at the seed stage are more prone to seed predator attack than species that are rare since they are more likely to be colonised by and to sustain viable seed predator populations (e.g. Pacala & Crawley 1992; Hanski 2001)
Maximum tree height	Species with large growth forms (e.g. canopy trees) are more prone to seed predator attack than species with smaller growth forms (e.g. shrubs and understory trees) since local seed crop sizes are likely to be bigger and more apparent to enemies (Janzen 1968)
Confamilial species on BCI	Species with many confamilial species in the local plant community are more prone to seed predator attack than phylogenetically isolated species (Janzen 1968), since the abundance of resources available to seed predators specialised at the family level will be higher
Congeneric species on BCI	Species with many congeneric species in the local plant community are more prone to seed predator attack than phylogenetically isolated species (Janzen 1968) since the abundance of resources available to seed predators specialised at the genus level will be higher
Local abundance of adult trees	Species that are locally abundant as adults are more prone to seed predator attack than species that are locally rare since they are more likely to be colonised by and to sustain viable seed predator populations (e.g. Pacala & Crawley 1992; Hanski 2001)
*Variables reflecting seed size and investment in seed defences*	
Seed mass	Species with large seeds are more prone to seed predator attack than species with small seeds since their seeds provide larger quantities of resources to developing seed predators (cf. Fenner *et al. * 2002)
Endocarp investment	Species that invest little in protective tissues surrounding the seeds are more prone to seed predator attack than species that invest large amounts of resources in seed protection (cf. Kuprewicz & García‐Robledo 2010)
Polyphenol concentration	Species with low investment in polyphenol production are more prone to seed predator attack than species with high polyphenol concentrations in their seeds (Janzen 1971; McArt *et al. * 2013)
*Variables reflecting temporal patterns in fruit fall*	
Interannual variation in seed crop sizes	Species with temporally predictable fruiting patterns are more prone to seed predator attack than species with large interannual variation in fruit crop sizes (Janzen 1971, 1976) since they provide a more stable resource base for specialist seed predators
Fruiting season	The proneness to seed predation varies between species fruiting in the wet *versus* the dry season if the abundance of seed predators varies with patterns of rainfall (cf. Wolda 1988)
Overlap in fruit production by other species	Species fruiting at times of the year when few other species fruit are more prone to seed predator attack than species that fruit when many co‐fruiting species fruit (Kelly 1994)
*Other*	
Growth form	Lianas are more prone to seed predator attack than trees, since they invest less in defence chemicals (Asner & Martin 2012; but see Gripenberg *et al. * 2018)
Relative growth rate (RGR)	Fast‐growing species tend to invest less in defense and are therefore more prone to seed predator attack than slow‐growing species (Coley *et al. * 1985)

## Methods

### Study site

Our study targeted tree, shrub and liana species on Barro Colorado Island (BCI), a 15.6 km^2^ island, located in Lake Gatun in central Panama. Its flora is exceptionally well known (e.g. Croat 1978; Leigh 1999), and long‐term studies of a 50‐ha forest dynamics plot (Condit 1998; Hubbell *et al.* 1999, 2005) have yielded unprecedented data on spatial and temporal patterns of seed and fruit production (e.g. Harms *et al.* 2000; Wright *et al.* 2005; Wright & Calderón 2006). Seed predation by insects has been studied in detail on a small number of plant species (e.g. Wright 1983; Jones & Comita 2010; Visser *et al.* 2011), but there has been limited investigation of community‐level patterns of insect seed predation (but see Pinzón‐Navarro *et al.* 2010).

### Documenting plant‐seed predator relationships

Host relationships for endophagous insect seed predators can be established and quantified conveniently by rearing insects from collected seeds or fruits. We aimed to collect at least 200 seeds/fruits of each woody plant species fruiting during our study. To achieve this, we adopted several approaches. First, sections of the ~ 40 km BCI trail network were walked each week and freshly fallen, intact‐looking seeds (individual diaspores) and fruits (seed‐bearing structures with one or more seeds) were collected from the forest floor. Seeds/fruits on branches that could be reached by hand or using a telescoping pole pruner were also occasionally collected to increase samples sizes. Second, each month we generated a target list that included species known to produce seeds/fruits at that time of the year (based on data from S. J. Wright’s long‐term seed monitoring project; Zimmerman *et al.* 2007) and for which the opportunistic trail walks had not yet yielded the target of 200 seeds/fruits. A team of botanical field technicians was tasked with collecting seeds from species on this list, generating samples of species not encountered during the trail walks and boosting sample sizes for rarer species. Third, we also obtained seeds and fruits from a network of seed fall traps in the 50‐ha forest dynamics plot (for details, see below). Seed collections took place between July 2010 and November 2013. Although our target was the plant community of BCI, a small subset (2.4%) of seed/fruit samples were collected on surrounding mainland peninsulas within approximately 2 km of BCI with a similar forest composition.

Seeds and fruits were sorted according to species and degree of maturity (mature*/*immature), counted, and stored in plastic pots lined with absorbent paper and covered with fine nylon mesh. All samples (seeds/fruits of a given species collected on a given day at a particular site) were identified to species. The pots were stored on shelves in a shade house in conditions resembling ambient forest understory conditions for three months. Given the biology of endophagous seed predators, we consider the possibility of transmission of insects between individual seeds/fruits in the rearing pots extremely unlikely.

During the rearing period, each pot was checked approximately twice a week and emerging adults and larvae were removed, processed and identified by morphological or molecular methods (Supporting Information, Appendix S1). At the end of the 3‐month rearing period each seed was carefully examined and dissected to check for evidence of insect attack such as exit holes, feeding tunnels, presence of frass or dead or living insects. Seeds with apparent insect damage but where other possibilities (such as fungal attack causing the interior of the seed to deteriorate) could not be ruled out were not scored as predated by insects.

We derived three variables reflecting susceptibility of plant species to seed predator attack: the *incidence of seed predators* (whether seed predators were reared from the plant species), *seed predator richness* (number of seed predator species reared from the plant species) and *seed predation rate* (proportion of dissected seeds showing signs of seed predator attack). To account for dependence of the incidence of seed predators and seed predator richness on sample size, we restricted certain analyses (identified below) to a subset of the data comprising plant species with ≥ 200 seeds (hereafter referred to as well‐sampled plant species). Above this sample size, a statistically significant relationship between the likelihood of seed predator detection and sample size could not be detected (Fig. S1). To check the robustness of our results to identification errors, we repeated several analyses excluding interactions documented only once (details below).

### Role of plant phylogeny and traits on patterns of seed predator host use

To test for phylogenetic signal in patterns of seed predator attack across the plant community we used the *D* statistic (Fritz & Purvis 2010) for incidence and Blomberg’s K (Blomberg *et al.* 2003) for seed predator richness and seed predation rates. In all cases, we used a phylogeny provided by David Erickson (Smithsonian Institution) that was constructed following methods in Kress *et al. *(2009). This phylogeny included 362 of our sampled plant species. Where sampled plant species were missing from the phylogeny but congeners were present (*n* = 59 species), they were added at the root of the genus using the *add.species.to.genus* function of the R package phytools (Revell 2012). Analyses were implemented using the *phylo.d* function of the R package caper (Orme *et al.* 2018) and the *multiPhylosignal* function in the R package picante (Kembel *et al.* 2010). For details, see Appendix S1.

To assess whether particular traits or trait values hypothesised to make plants particularly susceptible to enemies (Table 1) influenced seed predator attack we compiled data on relevant plant species traits from a range of sources (Appendix S2). This data set was at least 50% complete for all of our well‐sampled species (Appendix S1). We used random forest models (Cutler *et al.* 2007) to examine whether these traits were informative for predicting seed predator incidence, richness, and seed predation rates. Random forest algorithms combine many classification or regression tree models to produce more robust predictions. The technique can identify valuable predictors from heterogeneous data sources with missing values and does not require the prior specification of relationships between traits and response variables. Analyses were conducted for each of our three response variables. Only well‐sampled plant species (*n* = 214) were included in the main analyses. Since we tested a mixture of categorical and continuous predictors, to minimise variable selection bias we used conditional inference trees via the *cforest* function in the R package party (Strobl *et al.* 2007). Model hyperparameters are given in Appendix S1. To assess the overall ability to predict seed predator incidence, we used Cohen’s κ $\left(\frac{Accuracy-Baseline\;Accuracy}{1-Baseline\;Accuracy}\right)$to determine the extent to which the model can improve upon an expected level of classification accuracy (Landis & Koch 1977). For seed predator richness and seed predation rates we based our assessment on pseudo‐*R* ^2^ values. We compared the relative importance of each predictor trait using variable importance plots, which measure the mean decrease in accuracy of the model when the focal trait is randomised. We also conducted parallel analyses in which all plant species were included along with sample size as an additional predictor variable.

### Constructing quantitative plant‐seed predator food webs

To provide a visual and quantitative description of patterns of host use by seed predators, a set of quantitative food webs (one overall web and two taxon‐specific webs focusing on Coleoptera and Lepidoptera, respectively) were constructed and analysed using the R package bipartite (Dormann *et al.* 2008). To construct a fully quantified food web (e.g. Müller *et al.* 1999; Lewis *et al.* 2002), information on host densities and the frequency of trophic interactions between hosts and enemies is needed.

#### Estimating host densities

Data on species‐specific seed abundances per unit area were obtained from S. J. Wright’s long‐term study on seed and fruit production in the 50‐ha plot (see Appendix S1). In brief, we used information on the number of seeds and fruits falling into a network of 200 seed traps (each 0.5 m^2^) during 1987–2015, along with information on the average number of seeds per fruit. Since many seed predator species in our data set are likely to be pre‐dispersal seed predators (S. Gripenberg, pers. obs.), we quantified host seed abundances based on both immature and mature fruits and seeds. Of 486 plant species observed in the seed traps, 357 (74.5%) were collected for insect rearing. Of the 129 species we failed to collect, 75 were not detected in the seed traps during the period of our study and the remainder were collected in very small numbers (<25 seeds). Conversely, seeds were collected for insect rearing from 122 plant species that did not appear in the seed traps; these species were excluded from the food webs but used in other analyses.

#### Estimating the number of seeds killed per seed predator species

For each predator × prey association in the data set, we estimated the typical number of insect individuals emerging per attacked seed by dividing the number of insect individuals emerging by the number of seeds scored as predated. In most cases, only one insect could develop successfully in a seed. Since assessing the number of Hymenoptera individuals per infested seed proved difficult, our food webs do not include Hymenoptera (which comprised only 2% of seed predator individuals). We encountered multiple cases where one or several seed predator individuals fed inside fruits containing multiple small seeds (e.g. *Ficus* spp., *Apeiba* spp.), killing an unknown proportion of seeds. Since our methods did not allow us to estimate the proportion of seeds killed by these seed predators, these species (*n* = 16, 4.5% of the species that could potentially have been included in the food web) were excluded. A further 22 (6.2%) plant species were excluded from the food webs since the metadata needed to assess interaction frequencies (e.g. mean number of seeds per fruit) were not available. The resulting food web data set included information for 322 predator‐prey associations.

### Seed predator specialisation

The degree of seed predator specialisation is reflected visually in the quantitative food web plots, and was assessed quantitatively using Blüthgen *et al.*’s species‐level specialisation index (d’). This metric can be interpreted as the ‘deviation of the actual interaction frequencies from a null model which assumes that all partners are used in proportion to their availability’ (Blüthgen *et al.* 2006). To facilitate comparisons between taxa, we calculated d’ values for species within each order (Coleoptera, Lepidoptera) in the quantitative food web as well as for all seed predator taxa combined. We also calculated a specialisation index for the entire network (H2’; Blüthgen *et al.* 2006). Using the larger data set that included species not found in the seed traps in the 50‐ha forest dynamics plot we also produced histograms showing the frequency distribution of species against different diet richness values (the number of plant species recorded in the diet of each seed predator species; Futuyma & Gould 1979). As for d’, we examined the distribution of diet richness for all seed predators combined, as well as separately for each insect order (including Hymenoptera). Since limited sampling effort could inflate apparent specialisation, we limited diet richness analyses to insect species with a sample size of ≥ 10 individuals.

### Potential for enemy‐mediated indirect interactions

Using the food web data set, we assessed the potential for seed predator‐mediated indirect interactions following Müller *et al. *(1999):

$$d_{\mathrm{ij}}=\sum_{k}\left[\frac{\alpha_{\mathrm{jk}}}{\sum_{l}\alpha_{\mathrm{il}}}\frac{\alpha_{\mathrm{jk}}}{\sum_{m}\alpha_{\mathrm{mk}}}\right]$$

where *d_ij_* is the probability that that a seed predator attacking species *i* developed on species *j*, α is the link frequency, the summation *m* is over all host species from 1 to H (where H is the total number of host species), *k* is a seed predator species, and the summation *l* is over all seed predator species from 1 to P (the total number of seed predator species). Pairwise *d_ij_* values were calculated for the quantitative food web data set using the *PAC* function in bipartite. To assess whether the potential for apparent competition was highest for closely‐related plant species, we used Spearman’s rank correlation to assess whether there was a relationship between PAC values and pairwise phylogenetic distances. Species pairs for which PAC = 0 were excluded from this analysis.

## Results

### Community‐level patterns of seed predator attack

We collected 9325 samples totalling 207 201 seeds and fruits representing 478 plant species, 281 genera and 78 families (Table S1). We reared 21 604 adult insects and 3843 larvae from these samples. 13 293 (61.5%) insect individuals representing 369 (morpho)species were considered highly likely to be seed predators based on principles outlined in Appendix S1. Of these, the majority (221 species; 11,063 individuals) were Coleoptera (notably Curculionidae and Bruchinae), while the remainder were Lepidoptera (111 species representing ten families; 1965 individuals) and Hymenoptera (37 species of Eurytomidae; 254 individuals). A total of 471 interactions between plant species and seed predator species was documented. Table S2 provides a list of these interactions along with taxonomic information and Barcode Index Numbers (Ratnasingham & Hebert 2013) of seed predator species. Data on sequenced insect specimens (including images) are available on BOLD (http://www.boldsystems.org) accessible through https://doi.org/10.5883/DS-BCISP.

**Figure 1:**
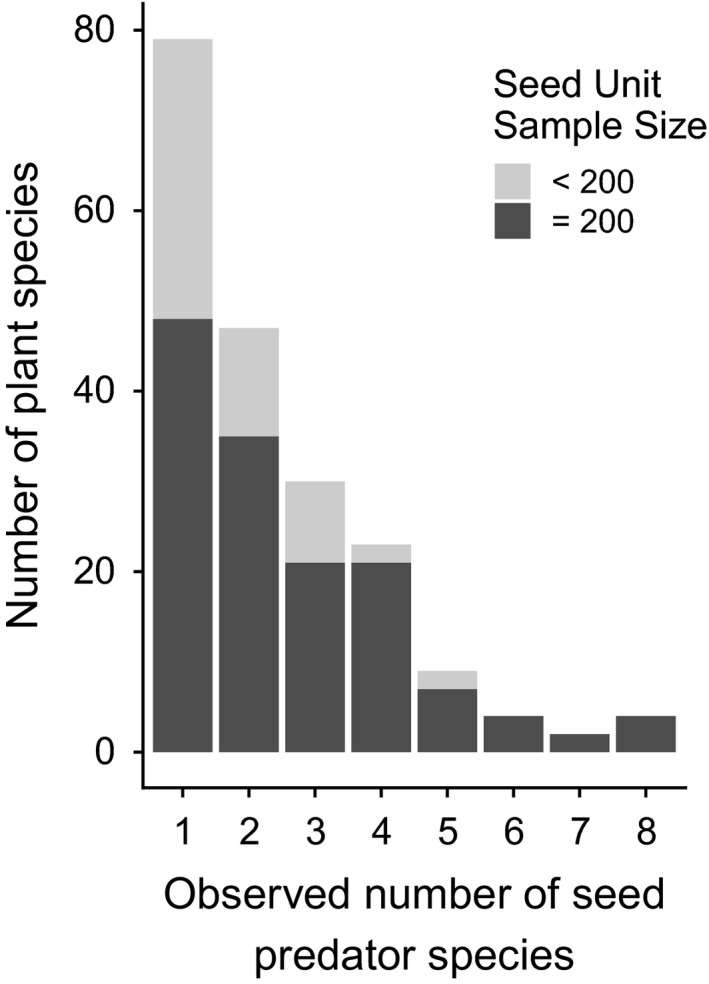
Frequency histogram showing the number of plant species associated with different numbers (*n* = 1–8) of seed predator species. Poorly sampled plant species (< 200 seeds/fruits collected for insect rearing) are shown in light grey. The number of species with no seed predator species were 95 and 212 for well‐sampled and poorly‐sampled plant species, respectively.

Seed predators were reared from 199 of the 478 sampled plant species (41.6%). Since the likelihood of rearing seed predators increased with sample size (Figure S1), the true proportion of plant species attacked by seed predators is likely to be higher. Considering only the 223 well‐sampled plant species, 142 species (63.6%) were attacked by seed predators. The average number of seed predator species per attacked plant species was 2.4 (range 1–8; median 2) for the full data set, and slightly higher (2.6; range 1–8; median 2) for well‐sampled species (Fig. 1). Across the network, estimated interaction coverage was 0.74 (Chao1 estimator; Appendix S3).

**Figure 2:**
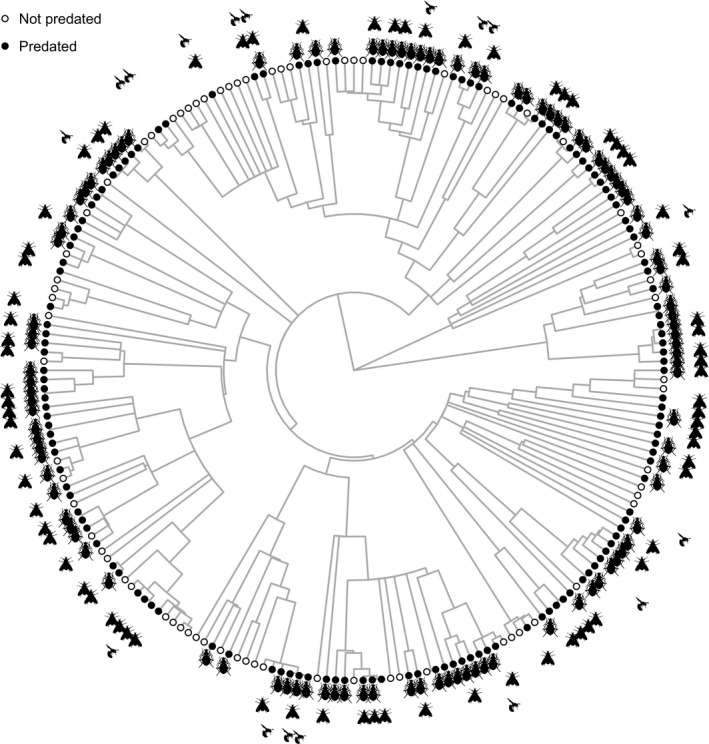
Presence (black circle) or absence (open circle) of seed predators and incidence of seed predator orders (Coleoptera, Lepidoptera, Hymenoptera) plotted against a plant phylogeny that includes only plant species with a minimum sample size of 200 seeds/fruits. Plant species names can be seen in the larger phylogeny shown in Figure S2. The figure was drawn using the R package ggtree (Yu *et al.* 2017).

Seed predators were associated with plant species across a wide range of the plant phylogeny (Fig. 2). For well‐sampled plant species, a weak phylogenetic signal in incidence of seed predators was detected (D = 0.779, P_D<1_ = 0.006; see Table S3 for results from order‐specific analyses which are qualitatively similar). Although close to being statistically significant, the phylogenetic signals in seed predator richness (K = 0.004, P = 0.051) and seed predation rate (K = 0.006, P = 0.082) were negligible.

### Plant traits as predictors of community‐level patterns of seed predator attack

Using the data set which included only well‐sampled plant species, the variables examined in the random forest models only explained a small proportion of the variation in observed patterns of seed predator attack across the plant community. In terms of seed predator incidence*,* Cohen’s κ was 0.049, indicating that the predictor variables added very little information (Landis & Koch 1977). Likewise, the pseudo‐*R* ^2^ values of the models assessing the effect of seed predator richness (*R* ^2^ = 0.098) and seed predation rate (*R* ^2^ = 0.047) were very small.

**Figure 3:**
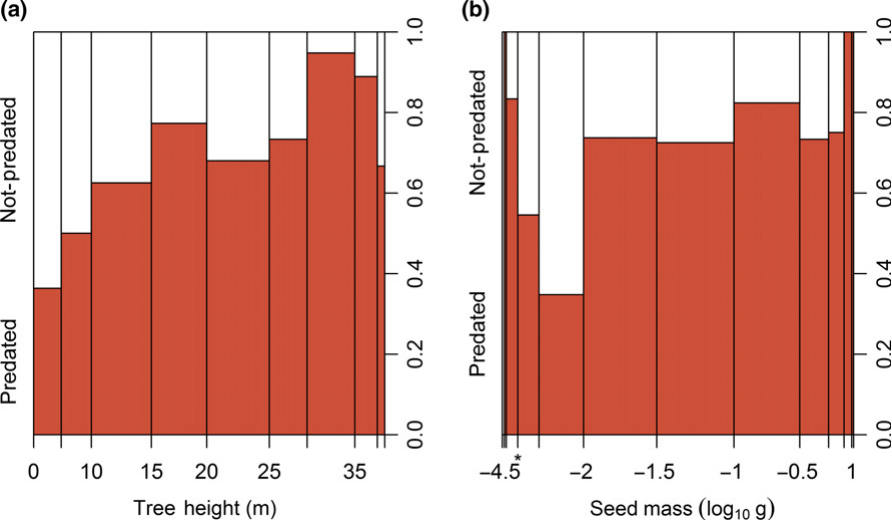
Spine plots showing relationships between the incidence of seed predation and (a) tree height and (b) seed mass. Equivalent plots for other traits are given in Figure S3 and partial dependence plots showing the predictions of random forest models as trait variables are changed are shown in Appendix S4. Note that panel a) includes only free‐standing species, since height (which is used as a proxy for seed crop size; Table 1) is not a meaningful trait for lianas. Internally‐feeding seed predators were never observed on species with seed masses smaller than 10^−3^ g (indicated by a star in panel b). Below this seed size, individual seed predators were consuming multiple seeds within a fruit.

Across the three response variables, the most important predictor variables were seed mass and maximum tree height (Appendix S4). Taller trees and larger seeds were more likely to be predated (Fig. 3), although the overall explanatory power of these relationships was weak. The relationship between seed predator incidence and all studied plant traits are shown in Figure S3. The results from the analyses on the full data set (*n* = 478 species) were qualitatively similar (Appendix S4).

### Host specificity of seed predators

**Figure 4:**
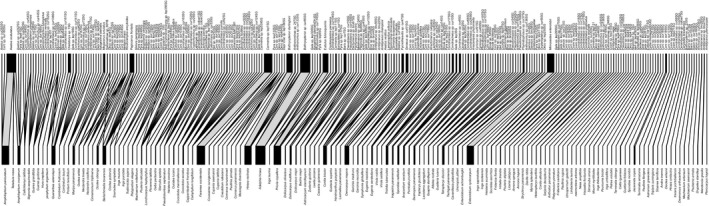
Bipartite network depicting the interactions between seeds and internally feeding insect seed predators in the 50‐ha forest dynamics plot on Barro Colorado Island. The lower bars show individual plant species and the upper bars show individual seed predator species. The widths of the bars reflect the abundance of seeds (lower bars) and the number of seeds killed by each seed predator species (upper bars), respectively. Species are organised according to the size of the network compartment they belong to. Order‐specific webs (Coleoptera and Lepidoptera) and a food web from which interactions documented only once (‘singletons’) have been excluded are shown in Fig. S4 and Fig. S5, respectively. An interactive version of the food web is available at http://bl.ocks.org/jcdterry/29fd8e581a27f0e861a71915ccaec938.

**Figure 5:**
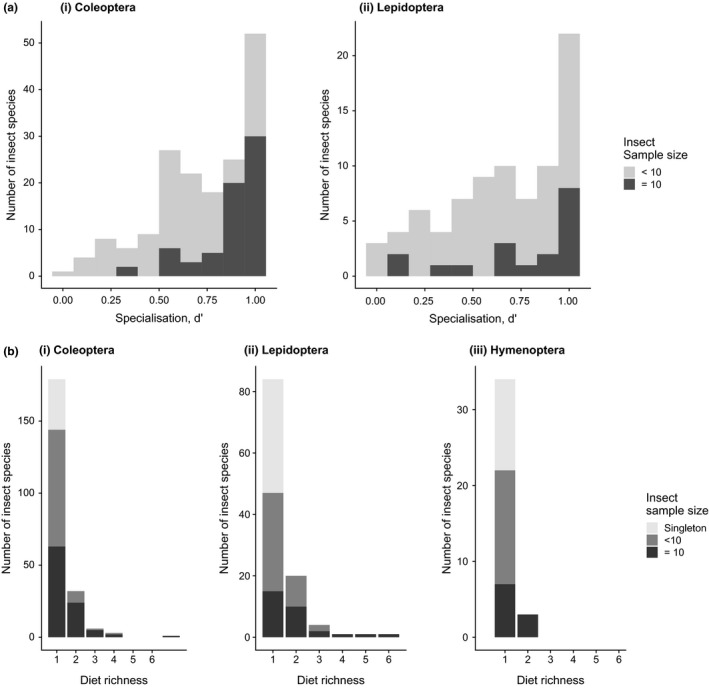
The distribution of diet specialisation among seed predator species displayed as (a) frequency histograms of d’ values of individual species in the food web focusing on the 50‐ha plot (displayed separately for Coleoptera and Lepidoptera) and (b) diet richness of seed predators using the full material (including insect and plant species not included in the food web). In panel (b), the histograms show the number of seed predator species in the data set reared from 1, 2, (…) and 7 plant species. Since the documented diet richness depends on sampling effort, the proportions of insect species in each diet richness category being singletons, species represented by 2–9 individuals, and species with ≥ 10 individuals are shown.

The quantitative plant‐seed predator web included 254 seed predator species feeding on 141 plant species (Fig. 4). Of 35 814 potential links only 322 were realised, yielding a connectance value of 0.009. The H_2_’ value of the network was 0.970, that is, close to the maximum possible value of 1. H_2_’ for the Coleoptera and Lepidoptera webs was 0.975 and 0.974, respectively. Specialisation values at the level of individual species, d’, ranged from 0.118 to 1 (median 0.830; Fig. 5a). Host specialisation (d’) did not differ significantly between Coleoptera and Lepidoptera (Wilcoxon rank sum test, W = 691, P = 0.284; analysis including insect species with ≥ 10 individuals).

The diet richness of seed predators (examined using the full insect rearing data set) showed that seed predator species fed on an average of 1.28 plant species (range 1–7). The diet richness was highly skewed towards monophagous species: of the 135 best‐sampled seed predator species (≥ 10 individuals reared), 85 (63%) were reared from a single plant species (Fig. 5b). The percentage of extreme specialists (species associated with a single host plant species) was 66.3, 50.0 and 70.0%) for Coleoptera, Lepidoptera and Hymenoptera respectively.

In 37.5% of cases where a seed predator species was reared from multiple plant species the host species were congeneric and in 61.1% they were confamilial. Measures of specialisation were robust to the inclusion of poorly‐sampled interactions and seed predator species (Table S4).

### Potential for seed predator mediated indirect interactions between plant species

The potential for seed predator‐mediated apparent competition between plant species was very low. In the data for the 141 plant species that were part of the quantitative food web, only 68 (0.34%) of the 19,740 potential pairwise enemy‐mediated interactions between plant species showed PAC values > 0.1 (corresponding to a situation where 10% of the enemies attacking the focal plant species are predicted to have developed as larvae on the alternative host species). Considering the species pairs for which PAC > 0, there was a significant negative relationship between PAC and pairwise phylogenetic distances (Spearman’s *r* = −0.249, *P* < 0.001; Fig. S6).

## Discussion

Documenting and understanding the ecological implications of the myriad of plant‐enemy interactions in hyper‐diverse forest communities is among the most challenging tasks facing tropical forest ecologists. Most such studies focus on particular plant or enemy taxa (e.g. Endara *et al.* 2017), or on subsets of plant species (e.g. Novotny *et al.* 2002). Our study includes approximately three quarters of the 652 woody plant species recorded at the study site (Croat 1978), and 90.5% of the species recorded as producing fruit there during the study. To the best of our knowledge, this is the largest and most complete data set yet available on plant‐enemy interactions in any tropical community, and it focuses on a guild of plant enemies which, through their direct effects on seed mortality, are expected to have pronounced effects on plant populations and communities.

We identified and quantified host associations for 369 seed predator species. While there was some evidence of phylogenetic clustering in patterns of attack, seed predators were widely distributed across the plant community (Fig. 1), with few plant clades and only 36.3% of well‐sampled plant species escaping this enemy guild. Clades where the majority of plant species were attacked were found within the Malpighiaceae and Fabaceae, whereas shrubs in the genus *Psychotria* (Rubiaceae) and the Melastomataceae genera *Mouriri*, *Conostegia*, *Leandra* and *Miconia* typically lacked seed predators.

One key aim of our study was to link patterns of seed predator attack to plant traits. Previous studies have found that interspecific variation in enemy attack can be explained by interspecific variation in particular traits or suites of traits (e.g. Carmona *et al.* 2011; Turcotte *et al.* 2014), including studies of leaf herbivory in tropical forests (e.g. Coley 1983, Schuldt *et al.* 2012, Cárdenas *et al.* 2014). However, our predicted associations between plant traits and seed predator attack were only weakly supported. The variables explaining the greatest proportion of observed variation were maximum tree height and seed mass. Traits associated with abundant resources were therefore associated with enemy attack, consistent with our predictions. Nevertheless, the plant traits investigated were poor predictors of community‐level patterns of seed predator attack, suggesting that seed predator host use is likely to be either stochastic or driven primarily by factors not investigated in this study. For example, seed predators may respond to chemical compounds other than polyphenols (e.g. Birch *et al.* 1986), or their patterns of host use might be influenced by predation risk (Lill *et al.* 2002).

Our most striking result is the remarkable degree of host specificity of seed predators. Although seed samples were obtained from 478 plant species, no seed predator species was recorded from more than seven host species, and 63% of the best‐sampled seed predator species (those with ≥ 10 individuals reared) were restricted to a single plant species. Given the local rarity and patchy distribution of most plant species in tropical forests (Condit *et al.* 2000), these specialised insects must have evolved highly efficient strategies for host location.

Host specificity was consistently high across insect orders. This contrasts with results from studies on fruit‐associated insects in Papua New Guinea (Ctvrtecka *et al.* 2014; Sam *et al.* 2017), where Curculionidae showed higher levels of specialisation than Lepidoptera. A possible explanation for the discrepancies in results between the two sites might be our more explicit focus on seed‐eating insects (i.e. plant antagonists). Had we included Lepidopteran families with members that are primarily pulp‐eaters or detritivores rather than seed‐eaters (e.g. Tineidae, Blastobasidae), we would likely have detected broader host ranges in Lepidoptera (S. Gripenberg; pers. obs.).

The high degree of host specificity is consistent with a role for seed predators in contributing to the maintenance of tropical forest plant diversity. The Janzen–Connell hypothesis (Janzen 1970; Connell 1971) proposes that enemy‐mediated negative density‐dependence favours plants that are locally rare, thereby promoting diversity at the community level. The Janzen–Connell mechanism requires that enemies are reasonably host specific (Sedio & Ostling 2013). Like the seed‐eating beetles of Guanacaste studied by Janzen (1980), the seed predators on BCI fulfil this criterion. Studies exploring spatial variation in seed predation rates on individual plant species are needed to evaluate further the role of seed predators in suppressing the reproductive output of their hosts in areas where the host is common (Gripenberg 2018). Our whole‐community focus meant that we were unable to quantify spatial variation in seed predation in relation to seed or plant abundance. Nevertheless, predation rates varied greatly among seed samples for individual species (S. Gripenberg, unpublished data), suggesting high spatial variation in rates of insect attack. Further studies are needed to investigate the extent to which this variation is caused by spatial variation in local seed densities.

Studies on tropical insect folivores have revealed lower levels of specialisation than previously assumed (e.g. Novotny *et al.* 2002). This opens up the possibility that insect enemies link the dynamics of tree populations indirectly, via apparent competition. However, few studies have tested for such indirect interactions, probably because predicting which species are most likely to interact via shared enemies requires accurate quantification of networks of trophic interactions across whole communities, data which are particularly challenging to collect in species‐rich tropical forests. On BCI we found little potential for apparent competition mediated by shared insect seed predators, one consequence of the high degree of seed predator host specificity documented. Given the unique features of insect seed predators such as their sessile feeding habit and their dependency on a resource that is typically only available for a small part of the year, the results from our study must not be uncritically extrapolated to other types of plant‐eating insects such as free‐feeding leaf herbivores. For example, seed predators are likely to be more host‐specific than many other herbivorous insect guilds (Novotny *et al.* 2010), leading to different effects on their host plant communities. Similarly, our results from BCI should also not be extrapolated uncritically to other sites: As noted above, Ctvrtecka *et al. *(2014) and Sam *et al. *(2017) found that seed‐ and fruit‐feeding insects in the lowland rainforests of Papua New Guinea were less specialised than those on BCI, so we cannot rule out a role for seed predators as agents of apparent competition in other tropical forests.

In summary, we have compiled and quantified the most complete network of trophic interactions yet documented for rainforest plants and their consumers. Focusing on a poorly‐studied group of plant enemies we found that species varied widely in their susceptibility to insect seed predator attack, partly explained by variables reflecting local resource abundance. The exceptionally high level of host specificity of seed predators of BCI suggests that these insects might contribute to maintaining the diversity of tree and liana species at our study site, while they are less likely to play an important role as mediators of indirect interactions among plant species.

## Authorship

SG and OL conceived the study. Protocols for data collection were designed by SG, OL, YB and SJW with input from SP‐N. Collection of seeds and fruits and insect rearing, sorting and morphotyping was carried out by IS, CF and MC‐S under the supervision of SG, SJW and YB. OC identified seed and fruit samples. MR morphotyped a subset of the insect specimens. Insect specimens were identified by HB, JB, AC, GM, DQ and RR. DNA barcoding of larvae was conducted by EV. SM coordinated Lepidoptera identifications and helped curate the DNA barcode library (managed by YB and SG). J‐PS and JK conducted analyses on seed polyphenols. CT conducted statistical analyses and prepared all figures. SG wrote the first draft of the manuscript. CT, OL, YB and SJW contributed to early revisions and all authors contributed to later revisions.

## Data Availability

Data associated with this manuscript are deposited in the Dryad Digital Repository : https://doi.org/10.5061/dryad.230j5ch. Code for analyses is available on GitHub (https://github.com/jcdterry/BCI_Seed_Predator). Data for sequenced insect specimens (including images) are available on BOLD (http://www.boldsystems.org), accessible by DOI (https://doi.org/10.5883/DS-BCISP).
