# Supplementary material for: A highly resolved food web for insect seed predators in a species‐rich tropical forest
Source: Ecol Lett. 2019 Jul 29;22(10):1638–49. doi: 10.1111/ele.13359 (PMC6852488; doi:10.1111/ele.13359)

Supplementary Information for *A highly-resolved food web for insect seed predators in a species-rich tropical forest*

S. Gripenberg *et al.*

Contents

1 **Appendix S1: Supplementary methods** **3**

2 **Appendix S2: Origins of data sets on plant traits** **12**

3 **Appendix S3: Sample coverage** **16**

4 **Appendix S4: Supplementary results from random forest analyses** **19**

5 **Table S1: List of sampled plant species** **23**

6 **Table S2: List of documented interactions between plant species and seed predator species** **39**

7 **Table S3: Phylogenetic signal in seed predator incidence, seed predator richness, and seed predation rate** **57**

8 **Table S4: Analyses on host specialisation using a data set that excludes singleton observations** **58**

9 **Figure S1: Effect of sample size on seed predator incidence** **59**

10 Figure S2: Plant phylogeny including all plant species 60

11 Figure S3: Spine plots depicting relationships between seed predator incidence and plant traits 61

12 Figure S4: Quantitative food webs for Coleoptera and Lepidoptera 63

13 Figure S5: Quantitative food web excluding singleton observations 65

14 Figure S6: Relationship between potential for apparent competition and phylogenetic distance 66

1 Appendix S1: Supplementary methods

Processing the insect material to obtain (morpho)species identifications

When encountered in rearing pots, adult Coleoptera, Diptera and Hymenoptera were transferred to vials with ethanol, while Lepidoptera were spread and dried. Where insects (typically weevils) emerged from the seed and fruit samples as larvae, they were stored in 95% ethanol to allow molecular identification through DNA barcoding (Hebert *et al.*2003). Where multiple similar-looking larvae were encountered in the same rearing pot on the same day they were assumed to belong to the same species. In such cases, one individual was stored in ethanol while remaining larvae were placed in pots with sterilised soil in an attempt to rear adults for morphology-based identification. Many (72.5%) of the 1889 larvae placed in soil pots did not emerge as adults. The likely species identity of these larvae – and of larvae in ethanol that were not DNA barcoded – was inferred from the DNA barcode of the paired larval samples stored in ethanol or from the identity of any adults emerging from the same soil pot.

All adult insects emerging from seeds or soil were assigned to a morphospecies based on their morphology. Initially, insect specimens reared from different plant species were processed separately and given different morphospecies-by-plant species codes. These were later merged to true species (or morphospecies) based on examination of selected specimens by experts on each taxonomic group. To ensure that the material did not include cryptic species (e.g. Hebert *et al.* 2004), up to 5 individuals per morphospecies excluding Hymenoptera (a total of 2733 adults and 244 larvae) were DNA barcoded (i.e. the mitochondrial COI gene sequenced; Hebert *et al.* 2003). DNA sequencing of adult specimens was done at the Biodiversity Institute of Ontario using their standard protocols and primers (Wilson 2012). DNA barcoding of larvae was done by Eero Vesterinen at the University of Turku. Barcode Index Numbers (Ratnasingham & Hebert 2013) were obtained for 35.8% of the (morpho)species in the full material and for 72.3% of the (morpho)species classified as seed predators (see below). In most cases there was a close match

between our initial morphospecies codes and the DNA barcodes, although in a few cases (notably within the Bruchinae), species assignments based on morphological characters proved difficult for non-specialists.

Assigning insect species and morphospecies to feeding guild

The seed and fruit samples inevitably yielded some insects that were not true seed predators, but pulp feeders, scavengers or fungivores, as well as natural enemies (parasitoids) associated with seed predators or members of the above-mentioned feeding guilds. In the context of the current study, we were exclusively interested in insects that are true seed predators, i.e. those that suppress the reproductive output of their hosts by killing seeds. Based on information about the ecology of different taxa, our own observations, and discussions with experts on different taxonomic groups, each insect species was assigned to its most likely feeding guild (seed predator versus other), following the principles outlined below: Of the Coleoptera, all bruchids (Bruchinae), Curculionidae (with the exception of Scolytinae; see below) and longhorn beetles (Cerambycidae) were classified as seed predators. While the majority of Scolytinae are likely to be pulp feeders or feeding on endocarps or the woody parts of fruits, one species (*Pagiocerus frontalis*) was classified as a seed predator based on clear evidence of feeding damage on the seeds of all of its documented host species (S. Gripenberg, *pers. obs.*). In terms of Lepidoptera, all members of the families Cosmopterigidae, Crambidae, Heliodinidae, Oecophoridae, Pyralidae, Sesiidae and Tortricidae were assumed to be seed predators. Based on expert opinion (Robert Robbins, *pers. comm.*) and feeding damage observed to seeds, three genera of Lycaenidae (*Strymon*, *Strephonota* and *Tmolus*) were assigned as seed predators. Tineidae were assumed to be scavengers, although the family includes diverse larval habits (Robinson 2009). Blastobasidae are historically assumed to be scavengers, although some species may feed on living tissue in fruits (Adamski *et al.* 2010). Most Hymenoptera reared from our samples are likely to be parasitoids of seed and fruit eating insects. Members of two families (Agaonidae and Eurytomidae) were labelled as seed predators based on what is known about their feeding habits in other contexts

(Donald Quicke, *pers. comm.*). In terms of Diptera, we recognise that some species reared from our seed and fruit samples (notably members of the families Tephritidae and Lonchaeidae) are likely to be seed predators. Nevertheless, since our data do not allow us to confidently assign individual dipteran species to the correct feeding guild we have refrained from including this species-rich order in our analyses. It will, however, be included in future analyses focusing on the wider insect communities associated with seeds and fruits (Basset *et al.*, in prep.). Overall, we believe our criteria for assigning species to feeding guild are strict: it is more likely that we have excluded seed predators than included non-seed predating species in our analyses. We note that there is a possibility that some seeds attacked by species scored as seed predators might still be viable (e.g. *Prioria copaifera* seeds with signs of insect seed predator attack have been observed to germinate; Dalling *et al.* 1997). Nevertheless, in the vast majority of interactions documented in this study, we believe the effect of the insects scored as seed predators to be lethal.

Details on analyses testing for phylogenetic signal

The *D* statistic (Fritz & Purvis 2010) which was used to test for phylogenetic signal in the incidence of seed predators across the plant community assesses the sum of changes in estimated nodal values of a binary trait across a phylogeny. The value of *D* was compared to *D* values found under models of phylogenetic randomness and evolution under Brownian motion. *D* values significantly smaller than 1 indicate that the examined trait is phylogenetically clumped and *D* values equal to 0 suggest that the trait is as clumped as if it had evolved under Brownian evolution. Whether observed *D* values deviated significantly from 1 and 0 was assessed using 1000 simulations (for details, see Fritz & Purvis 2010). Analyses were conducted for all seed predator taxa combined, and for each order (Coleoptera, Lepidoptera, Hymenoptera) separately. We tested for phylogenetic signal in seed predator richness and seed predation rates using Blomberg's *K* (Blomberg *et al.* 2003). To obtain P-values associated with *K*, we used the R package phytools (Revell 2012). To minimise potential issues resulting from variable sample sizes and incomplete sampling, only plant species with a minimum sample size of 200 seeds/fruits were included in

Table S1.1 Fraction of missing data for each trait in the plant dataset.

Trait	Well-Sampled Missing %	All Data Missing %
Lifeform	0.0	0.8
BCI genus-level diversity	3.3	7.3
BCI family-level diversity	8.0	6.1
Overlap in fruit production	8.5	26.7
Seed dry mass	14.6	30.7
Endocarp investment	14.6	30.1
Local Seed crop size	28.6	52.0
Interannual crop size variation	30.0	53.0
Tree height	34.3	50.5
Local Abundance	34.7	51.1
Relative Growth Rate	48.8	67.4
Polyphenol concentration	49.8	70.8

the analyses on phylogenetic signal.

Details on random forest analyses relating plant traits to seed predation Data were collated from a number of different sources, listed in Appendix S2. When assessing seed predation rate, we excluded plant species with seeds smaller than 1 mg, since our observations suggested that seeds smaller than this are likely to be too small to be attacked by endophagous seed predators. Amongst the well-sampled plant species (≥200 seeds/fruits) upon which we focussed our main analyses, the trait data completeness was above 50% for all the variables tested (Table S1.1). Missing values are dealt with by the default approach of the cforest() function, which uses surrogate splits where necessary (Hothorn *et al.* 2006).

Correlations between traits were generally low (Figure S1.1). The principal exception was seed crop variables (crop size, crop variation and fruit production overlap) that formed a distinct cluster. We sought to determine if a wide suite of traits could allow the prediction of our seed predator response variables with random forest models. This type of model builds a collection

of classification or regression trees to improve performance. The specification of random forest models can be altered by a wide range of 'hyperparameters', including the number and depth of classification trees and how the model's performance is tested. The results that we present made use of the default settings of the `cforest()` function in the party package (Table S1.2) with one exception: the number of trees per model was increased to 5000. While exploring the data we tested a wide variety of different combinations, as well as other random forest modelling frameworks, but there was little meaningful improvement. Model performance was tested with out-of-bag testing. In this approach a model is fit using all-but-one result, and the ability to fit the sample left out is examined.

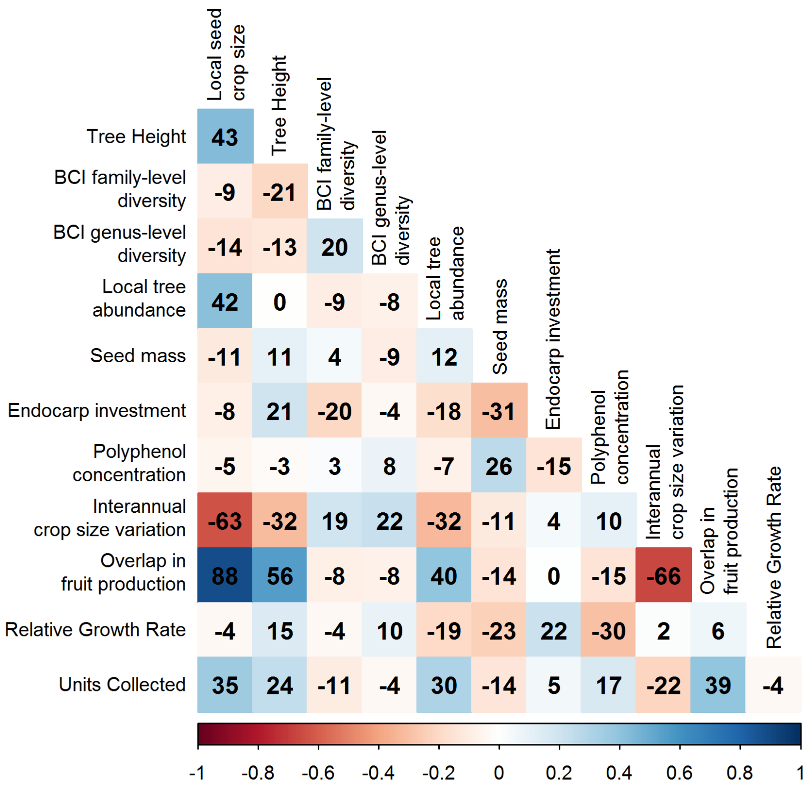

Figure S1.1. Correlations between the numeric continuous seed traits and sample size. Numbers and colour show Spearman's rank correlation coefficient (rescaled to percentages for conciseness).

Table S1.2 Values of hyperparameters used for reported results.

Setting name in cforest()	Explanation	Value
mtry	The number of variables to use in each tree	5
ntree	The number of classification or regression trees to include in each model	5000
mincriterion	The value of the test statistic that must be exceeded to implement a split	0 (i.e unbounded trees)

Comparing the role of different variables in driving the accuracy of predictions is challenging in a random forest model, especially when the variables have differing levels of missingness, are sometimes categorical and may be somewhat correlated. Variable importance measures examine the mean decrease in accuracy if a predictor variable is randomised. To gain some insight into the driving features of our model we used the `varimp()` function in the party package. Since our data had a number of missing values, we used the approach detailed in Hapfelmeier *et al.* (2014).

Details on the estimation of species-specific seed abundances for the quantitative food web

Species-specific seed abundances were estimated based on a data set of seeds (or individual diaspores) and fruits (i.e. seed-bearing structures with one or more seeds) collected from a network of 200 seed traps (each 0.5m^2 in size) in the 50-ha Forest-GEO forest dynamics plot (Anderson-Teixeira *et al.* 2015) on a weekly basis from 1987 to 2015. For each species, the total counts of the following seed and fruit parts falling into the traps were recorded: *mature fruits*, *single diaspores* (i.e. 'seeds'), *capsules* (part that vertebrates never eat; botanically this might be a capsule, pedicel, bract, etc.), *fragments of fruit* dropped by vertebrates (the number of fruit

represented was recorded by counting pedicels or the points of attachment to the mother plant), *immature fruits* (endosperm of seeds is not filled), *fruit with insect emergence hole* (only recorded for selected species), *aborted fruits* (fall soon after flowering, have a swollen ovule, and often have some flower parts attached), *fruit eaten by animal*. To estimate the total number of seeds produced for each species, we multiplied the number of fruits collected by the species-specific average number of seeds per fruit, and then added *either* the number of seeds *or* the number of capsules multiplied by species-specific average number of fruits per capsule multiplied by average number of seeds per fruit; whichever number was larger. The rationale for this approach is that since some of the seeds falling into the trap might have originated from the capsule, we might overestimate seed numbers if we were to use (fruits $\times$ average seeds/fruit) + seeds + (capsules $\times$ average seeds/fruit). Since many seed predators are likely to be pre-dispersal seed predators attacking the fruits before they reach maturity, immature fruits were included in the estimates.

For the majority (77.6%) of species, information on the typical number of seeds per fruit was obtained from fruit dissections carried out on BCI in the context of other research projects (S. J. Wright, unpubl. data). For remaining species, information on typical seed numbers per fruit was extracted from Croat (1978). (Where a range of seed numbers per fruit was reported for a species, we used the median value of this range.) For a small number of species (n=27) for which data on seed numbers per fruit were missing but for which there was information on seed numbers per fruit for congeneric species, we used the genus-specific average number of seeds per fruit (obtained from the above-mentioned sources). Following these approaches, we obtained an estimate for number of seeds per fruit for all but 11 species (3.1%) in the food web data set. We note that the species for which data on seeds per fruit were missing were typically berries, which were excluded from the food web for other reasons (estimating the proportion of seeds killed by seed predators was not possible for these species; see main text).

References for Appendix 1

1. Adamski, D., Copeland, R.S., Miller, S.E., Hebert, P.D.N., Darrow, K. & Luke, Q. (2010). A review of African Blastobasinae (Lepidoptera: Gelechioidea: Coleophoridae), with new taxa reared from native fruits in Kenya. Smithsonian Contributions to Zoology, 1-68.

2. Anderson-Teixeira, K.J., Davies, S.J., Bennett, A.C., Gonzalez-Akre, E.B., Muller-Landau, H.C., Wright, S. J. *et al.* (2015). CTFS-ForestGEO: a worldwide network monitoring forests in an era of global change. Global Change Biology, 21, 528-549.

3. Blomberg, S.P., Garland, T. & Ives, A.R. (2003). Testing for phylogenetic signal in comparative data: behavioral traits are more labile. Evolution, 57, 717-745.

4. Croat, T.B. (1978). Flora of Barro Colorado Island. Stanford University Press.

5. Dalling, J.W., Harms, K.E. & Aizprúa, R. (1997). Seed damage tolerance and seedling resprouting ability of Prioria copaifera in Panamá. J. Trop. Ecol., 13, 481-490.

6. Fritz, S.A. & Purvis, A. (2010). Selectivity in mammalian extinction risk and threat types: a new measure of phylogenetic signal strength in binary traits. Conserv. Biol., 24, 1042-1051.

7. Hapfelmeier, A., Hothorn, T., Ulm, K., & Strobl, C. (2014). A new variable importance measure for random forests with missing data. Statistics and Computing, 24, 21–34.

8. Hebert, P.D., Cywinska, A. & Ball, S.L. (2003). Biological identifications through DNA barcodes. Proc. R. Soc. Lond. B: Biol. Sci., 270, 313-321.

9. Hebert, P.D.N., Penton, E.H., Burns, J.M., Janzen, D.H. & Hallwachs, W. (2004). Ten species in one: DNA barcoding reveals cryptic species in the neotropical skipper butterfly Astraptes fulgerator. Proc. Natl. Acad. Sci. U. S. A., 101, 14812-14817.

10. Hothorn, T., Hornik, K. & Zeileis, A. (2006). Unbiased recursive partitioning: A conditional inference framework. J. Comp. Graph. Stat., 15, 651-674.

11. Ratnasingham, S. & Hebert, P.D.N. (2013). A DNA-Based Registry for All Animal Species: The Barcode Index Number (BIN) System. PloS One, 8, e66213.

12. Revell, L.J. (2012). phytools: an R package for phylogenetic comparative biology (and other things). Meth. Ecol. Evol., 3, 217-223.

13. Robinson, G.S. (2009). Biology, Distribution and Diversity of Tineid Moths. Southdene, Kuala Lumpur.

14. Wilson, J.J. (2012). DNA barcodes for insects. In: DNA Barcodes: Methods and Protocols (eds. Kress, WJ & Erickson, DL). Springer New York, pp. 17-46.

2 Appendix S2: Origins of data sets on plant traits

The data sets needed to assess the relationship between community-level patterns of seed predator attack (incidence of seed predators, seed predator richness, and proportion of seeds scored as predated) and traits hypothesised to make plant species more or less prone to seed predation (Table 1 in the main text) were obtained from a variety of sources:

Local seed abundance – Data used to quantify seed abundances of individual tree and liana species in the BCI community were obtained from S. J. Wright's long term study focusing on seed rain into a network of 200 seed traps (0.5 m2) located in the 50-ha forest dynamics plot on BCI (see e.g. Harms *et al.* 2000, Wright *et al.* 2005). For the species encountered in the seed traps, species-specific seed production was taken as a measure of seed abundance at the community level. Seed production was calculated by summing the counts of seeds falling into the seed traps during the period between January 1987 and October 2010.

Maximum tree height – Data on the maximum height (m) of trees were collected using methods described in Wright *et al.* (2010).

Confamilial species on BCI – For each sampled plant species, information on the number of plant species within the same family known to occur on BCI was obtained from a list of plant species recorded on BCI compiled by Carmen Galdames.

Congeneric species on BCI – Information on the number of plant species within the same genus known to occur on BCI was obtained from a list of plant species recorded on BCI compiled by Carmen Galdames.

Local abundance of adult trees – The local abundance of reproductive-sized adult trees was estimated using data from the 2010 census of the ForestGeo plot (Condit 1998, Hubbell *et al.* 1999, Hubbell *et al.* 2005). To estimate the number of reproductive adults in the 50-ha plot, we extracted species-specific maximum diameter at breast height (DBHmax) and selected all individuals with a DBH larger than 0.5 $\times$ DBHmax (the known size threshold for tree reproduction; Visser *et al.* 2016).

Seed mass – Species-specific seed masses were available in the form of mean dry seed mass (expressed in grams) where a 'seed' is defined to include the endosperm and embryo only. For the majority of species, the mean seed mass was based on an average of 5 seeds collected from 5 individuals and dried to constant mass at 60°C (for some species, sample sizes were slightly lower).

Endocarp investment – To obtain a measure of the degree of investment in mechanical seed defences, we used a largely unpublished data set on species-specific protective tissue content, reflecting the proportion of diaspore mass made up by protective tissue (e.g. endocarps and seed coats) rather than seed mass. These data were obtained by dissecting diaspores into three parts: seed (embryo plus endosperm only), appendages to enable dispersal by wind (wings for virtually all species), and material to protect the seed. All material was oven dried at 60°C for at least 72 hours and then weighed for dry mass. The protective tissue content was taken as the dry weight of the seed protection material divided by the diaspore dry weight.

Polyphenol concentration – Data on polyphenol concentration in seeds (mg/g dry seed mass) were obtained from a study by Gripenberg *et al.* (2017, 2018).

Interannual variation in seed crop sizes – As a measure of the extent of interannual variation in the size of the seed crop we used a variable analogous to the variable CVyear in Wright *et al.* (2005), but implemented on a larger data set involving more species and in which seed fall for each trap was averaged across a longer time period (1987 to 2010) than in the primary publication.

Fruiting season – Based on the mean fruit fall date (as obtained from above-mentioned seed traps), species were assigned to 'wet season species' (mean fruit fall date in the period between 1st June and 30th November) or 'dry season species' (mean fruit fall date in the period between 15th January and 30th March). Species fruiting in the transitional months (April, May, December and early January) were not classified.

Overlap in fruit production by other species – The overlap in fruit production between plant species was taken as the total number of other species observed to fruit in the same week as a given plant in the seed trap dataset associated with the study by Wright *et al.* (2016).

Growth form – Based on their growth form, species were classified as trees or lianas.

Relative growth rate – The relative growth rate (RGR; cm per year) of saplings were collected using methods described in Wright *et al.* (2010).

References for Appendix 2

1. Condit, R. (1998). Tropical forest census plots: methods and results from Barro Colorado Island, Panama and a comparison with other plots. Springer-Verlag, Berlin.

2. Croat, T.B. (1978). Flora of Barro Colorado Island. Stanford University Press.

3. Gripenberg, S., Rota, J., Kim, J., Wright, S.J., Garwood, N.C., Fricke, E.C. *et al.* (2018). Seed polyphenols in a diverse tropical plant community. J. Ecol., 106, 87-100.

4. Gripenberg, S., Rota, J., Kim, J., Wright, S.J., Garwood, N.C., Fricke, E.C. *et al.* (2017). Data from: Seed polyphenols in a diverse tropical plant community. Dryad Digital Repository.

5. Harms, K.E., Wright, S.J., Calderon, O., Hernandez, A. & Herre, E.A. (2000). Pervasive density-dependent recruitment enhances seedling diversity in a tropical forest. Nature, 404, 493-495.

6. Hubbell, S.P., Condit, R. & Foster, R.B. (2005). Barro Colorado Forest Plot Census Data. URL http://ctfs.si.edu/webatlas/datasets/bci.

7. Hubbell, S.P., Foster, R.B., O'Brien, S.T., Harms, K., Condit, R., Wechsler, B. *et al.* (1999). Light-gap disturbances, recruitment limitation, and tree diversity in a neotropical forest. Science, 283, 554-557.

8. Visser, M.D., Bruijning, M., Wright, S.J., Muller-Landau, H.C., Jongejans, E., Comita, L.S. *et al.* (2016). Functional traits as predictors of vital rates across the life cycle of tropical trees. Funct. Ecol., 30, 168-180.

9. Wright, S.J., Calderón, O., Hernandéz, A., Detto, M. & Jansen, P.A. (2016). Interspecific associations in seed arrival and seedling recruitment in a Neotropical forest. Ecology, 97, 2780-2790.

10. Wright, S.J., Kitajima, K., Kraft, N.J., Reich, P.B., Wright, I.J., Bunker, D.E. *et al.* (2010). Functional traits and the growth-mortality trade-off in tropical trees. Ecology, 91, 3664-3674.

11. Wright, S.J., Muller-Landau, H.C., Calderón, O. & Hernandéz, A. (2005). Annual and spatial variation in seedfall and seedling recruitment in a neotropical forest. Ecology, 86, 848-860.

3 Appendix S3: Sample coverage

Sample coverage estimators offer the potential to indicate how complete an interaction network is based on the frequency of observations (Jordano 2016). Coverage estimators are well established at estimating species diversity measures with under-sampling (Gotelli & Colwell 2010). The abundance-based Chao estimator for sample coverage (Chao 1984) is calculated as:

$$1 - \frac{f_1}{n} \frac{(n-1)f_1}{(n-1)f_1 + 2f_2}$$

where n is the total number of observed interactions, f_1 is the number of interactions observed just once, and f_2 is the number of interactions observed exactly twice. For our full network using all observed interactions, n = 471, f_1 = 123, and f_2 = 92, giving an estimated sample coverage of 0.7396. This suggests that 74% of all plant species $\times$ seed predator species interactions occurring in the community are represented in the sample, or in other words that (at least) 26% of the interactions remain unobserved. Note that this is distinct to network completeness, the proportion of 'present' interaction types that were observed. Using the `estimateR` function in the vegan package (Oksanen *et al.* 2018), the lower bound estimate for the total number of interactions was found to be 551.6. A rarefaction curve (Fig. S3.1) suggests that the rate of accumulation of interactions had significantly slowed. However, theory for the use of this approach designed for species richness is less developed for estimating interaction completeness (Jordano 2016), so it is unclear how close to this lower bound the true number of interactions is likely to be. The likely over-dispersion in our data, whereby clusters of observations of interactions are likely to occur, may bias these estimates, and as such they are only intended for use as an indicator that the sampling was sufficiently thorough to capture the principle trends.

Figure S3.1 Rarefaction curve of sample size against accumulated interaction count. Although the accumulation curve has not plateaued, the rate of accumulation is low at large sample sizes.

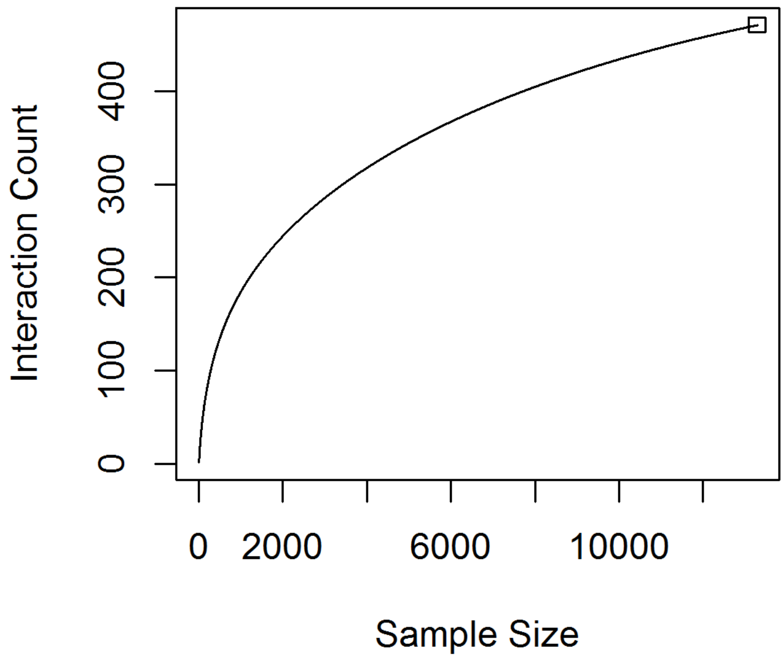

References for Appendix 3

1. Chao, A. (1984). Nonparametric estimation of the number of classes in a population. Scand. J. Stat., 11, 265-270.

2. Gotelli, N.J. & Colwell, R.K. (2010). Estimating species richness. In: Biological Diversity: Frontiers in Measurement and Assessment (eds. Magurran, AE & McGill, BJ). Open University Press, pp. 416-422.

3. Jordano, P. (2016). Sampling networks of ecological interactions. Funct. Ecol., 30, 1883-1893.

4. Oksanen, J., Blanchet, F.G., Friendly, M., Kindt, R., Legendre, P., McGlinn, D. *et al.* (2016).

vegan: Community Ecology Package. R package version 2.4-0. https://CRAN.R-project.org/package=vegan.

4 Appendix S4: Supplementary results from random forest analyses

The relative importance of individual plant traits in the analyses of the full data set are shown in Fig. S4.1. Although our models cannot predict our responses much better than random, it is notable that the same set of variables, in particular seed mass and tree height are consistently identified as important. This is despite tree height data only being available in 65% of cases (see Appendix S1) – many of the remainder are lianas, for which 'height' is not meaningful.

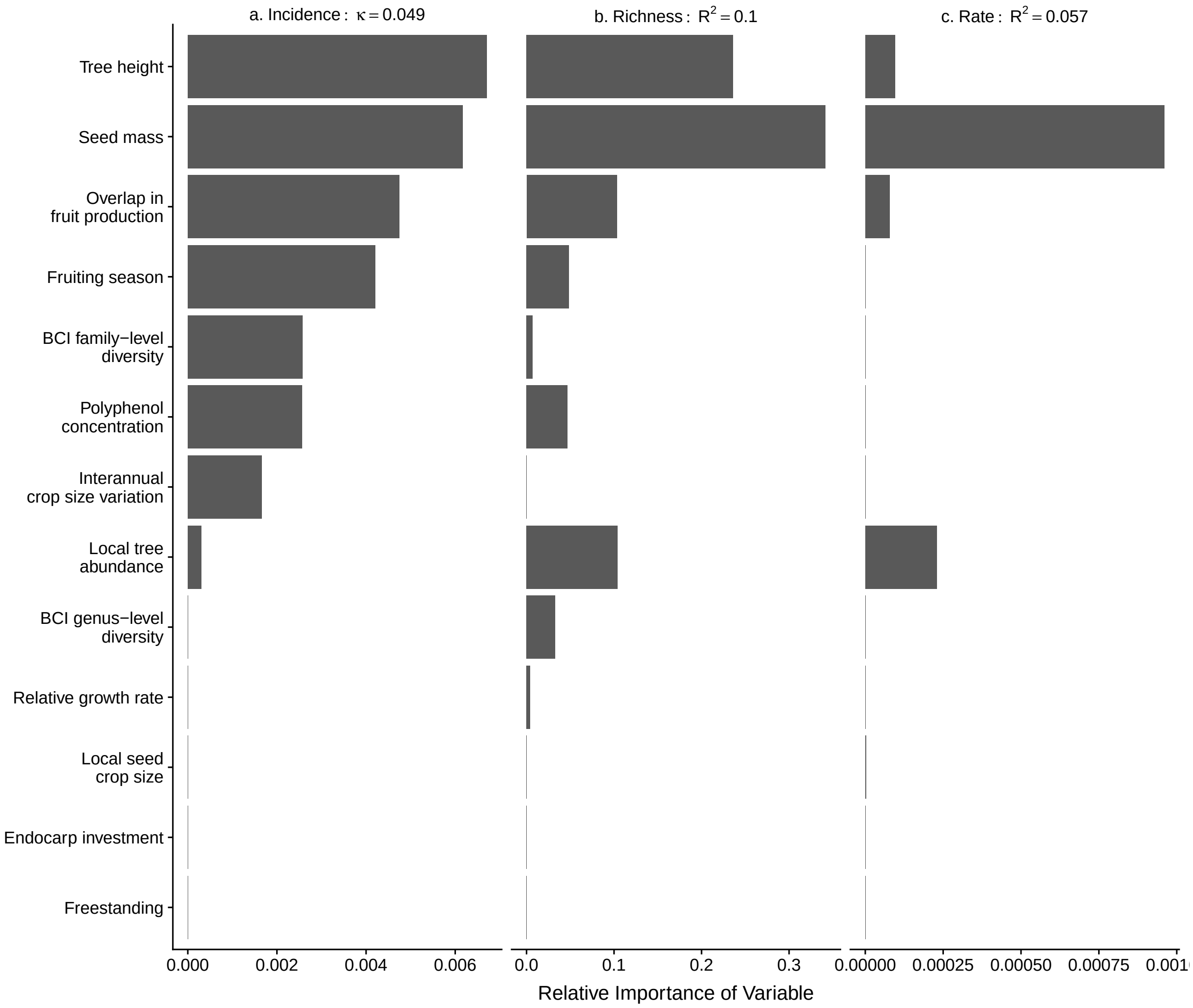

Figure S4.1. Relative trait variable importance for our three response variables for the 'well-sampled' dataset (including species with a minimum of 200 seeds/fruits). Variables are sorted according to their relative importance in explaining the incidence of seed predators.

An alternative approach to assessing how a model uses the information are partial dependence plots. These display how a model's average predictions change as a predictor variable is changed, all else being equal. The partial dependence plots for seed mass and tree height are shown in Fig. S4.2, and generally match the patterns in Fig. 3 in the main text.

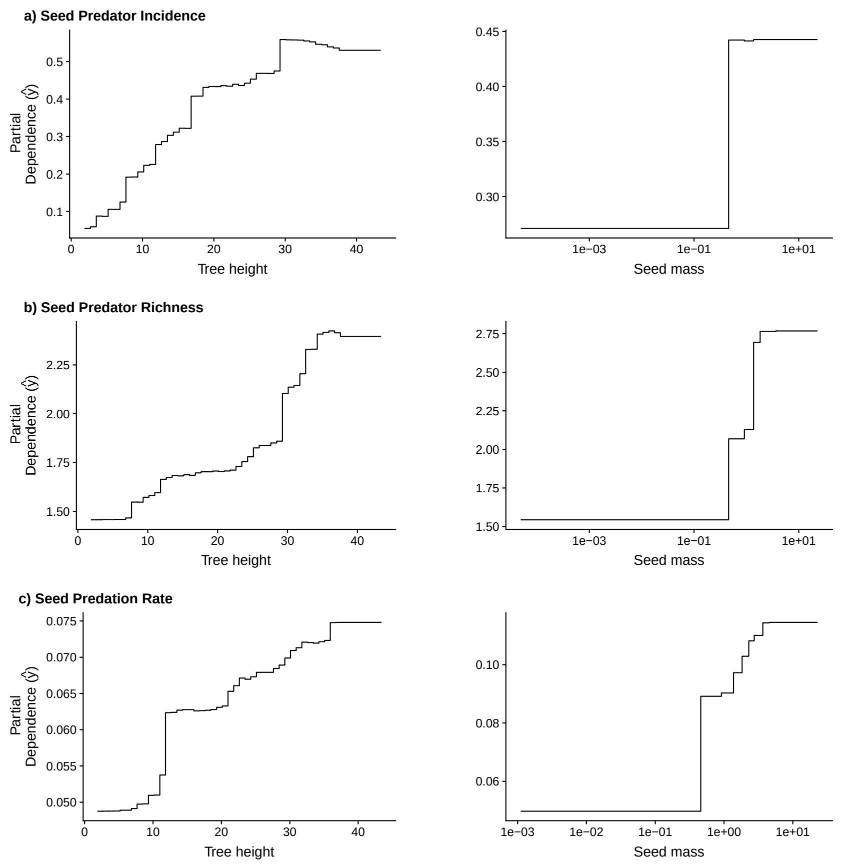

Figure S4.2. Partial dependence plots for tree height and seed mass. These plots show how the predictions of our random forest models change in response to shifts in the values of a particular trait, against a representative background of other traits. There is a positive relationship between tree height and the susceptibility of species to seed predator attack and between seed mass and the susceptibility to seed predator attack. Note the logarithmic scale for seed mass.

To confirm that filtering of plants to include only well sampled species was not affecting the results, we also conducted a parallel analysis in which all plant species were included along with sample size as an additional predictor variable. Seed predator incidence could be predicted quite well, (κ = 0.36), but by far the most important variable was the sample size ('Total Units Collected'; Fig. S4.3). Species richness was predicted moderately well (pseudo-R^2 = 0.248), but this was again dominated by sample size. Species predation rate was poorly predicted on the full dataset (pseudo-R^2 = 0.0455), with sample size not driving rate.

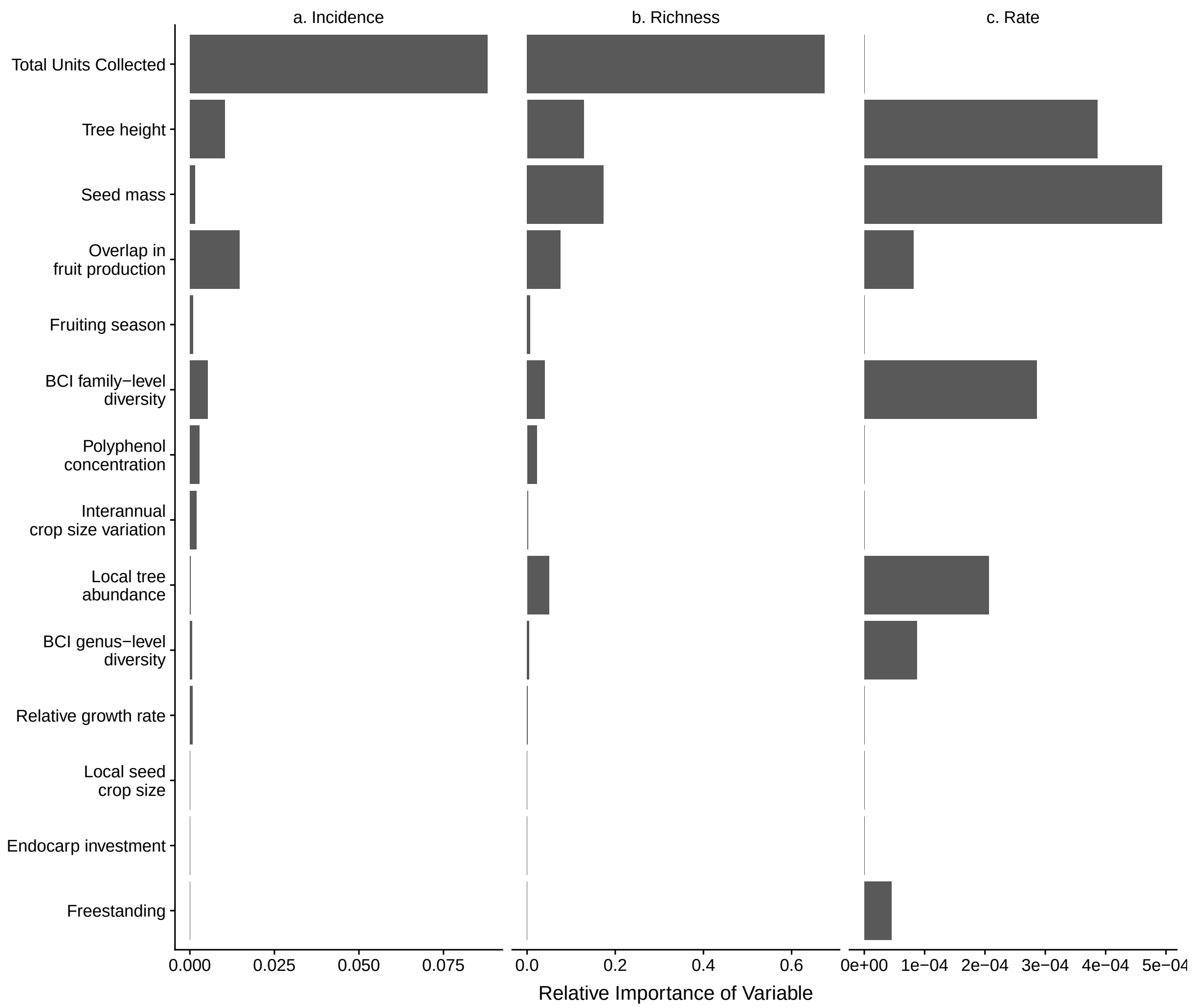

Figure S4.3. Relative variable importance plots using the full dataset, which includes poorly sampled species. For both incidence and richness, total units collected was a driving feature.

5 Table S1: List of sampled plant species

A list of the 478 plant species sampled for insect seed predators. For tree species occurring in the 50-ha forest dynamics plot, the nomenclature follows `http://conditdatacenter.org/taxonomy/BCI/BCIPlotFullTaxonomy.php` (accessed 14th December 2018). The nomenclature of remaining species follows `http://www.theplantlist.org` (accessed 8th January 2019). Code4 and Code6 are abbreviations of plant species names commonly used by researchers working on BCI. The column 'Units collected' shows the total number of seeds or fruits collected for insect rearing and the column 'Phylogeny' indicates whether or not (yes/no) the focal plant species was included in the phylogeny. Data on species-specific values of the 13 traits investigated in our analyses (Table 1 in the main text and Appendix S2) can be found in `https://github.com/jcdterry/BCI_Seed_Predator/` (to be made publicly available upon publication).

Species	Family	Code4	Code6	Units collected	Phylogeny
Abuta racemosa	Menispermaceae	ABUR	ABUTRA	47	Yes
Acalypha macrostachya	Euphorbiaceae	ACA2	ACALMA	287	Yes
Acalypha diversifolia	Euphorbiaceae	ACAD	ACALDI	83	Yes
Acacia polyphylla	Fabaceae	ACAG	NA	16	Yes
Acacia hayesii	Fabaceae	ACAH	ACACHA	196	Yes
Entadopsis polystachya	Fabaceae	ADEP	ADE4PO	183	Yes
Adelia triloba	Euphorbiaceae	ADET	ADE1TR	235	Yes
Aechmea setigera	Bromeliaceae	AECS	NA	8	No
Aegiphila cephalophora	Lamiaceae	AEGC	AEGICE	477	Yes
Aegiphila panamensis	Lamiaceae	AEGP	AEGIPA	186	Yes
Alchornea costaricensis	Euphorbiaceae	ALCC	ALCHCO	323	Yes
Alibertia edulis	Rubiaceae	ALIE	ALIBED	166	Yes
Allamanda cathartica	Apocynaceae	ALLC	ALLACA	9	Yes
Allophylus psilospermus	Sapindaceae	ALLP	ALLOPS	1063	Yes
Alseis blackiana	Rubiaceae	ALSB	ALSEBL	5511	Yes
Amaioua corymbosa	Rubiaceae	AMAC	AMAICO	76	Yes
Amphilophium paniculatum	Bignoniaceae	AMPP	AMPHPA	362	Yes

Anacardium excelsum	Anacardiaceae	ANAE	ANACEX	396	Yes
Anaxagorea panamensis	Annonaceae	ANAP	ANAXPA	801	Yes
Andira inermis	Fabaceae	ANDI	ANDIIN	737	Yes
Annona glabra	Annonaceae	ANNG	ANNOGL	16	Yes
Annona hayesii	Annonaceae	ANNH	ANNOHA	46	Yes
Annona spraguei	Annonaceae	ANNS	ANNOSP	104	Yes
Anomospermum chloranthum_isthmicola	Menispermaceae	ANOC	NA	89	No
Anthurium acutangulum	Araceae	ANTA	NA	350	No
Anthurium brownii	Araceae	ANTB	NA	2008	No
Anthurium clavigerum	Araceae	ANTC	ANTHCL	1484	No
Anthurium friedrichsthalii	Araceae	ANTF	NA	133	No
Anthurium ochranthum	Araceae	ANTO	NA	2	No
Anthodon panamense	Celastraceae	ANTP	ANTHPA	244	Yes
Apeiba membranacea	Malvaceae	APEM	APEIME	2070	Yes
Apeiba tibourbou	Malvaceae	APET	APEITI	711	Yes
Ardisia bartlettii	Myrsinaceae	ARDB	ARDIBA	183	Yes
Ardisia standleyana	Myrsinaceae	ARDF	ARDIFE	101	Yes
Ardisia guianensis	Myrsinaceae	ARDG	ARDIGU	136	Yes
Aristolochia gigantea	Aristolochiaceae	ARI2	ARISGI	3	Yes
Aristolochia tonduzii	Aristolochiaceae	ARIC	ARISTO	18	Yes
Fridericia candicans	Bignoniaceae	ARR1	ARRACA	127	Yes
Fridericia chica	Bignoniaceae	ARR2	ARRACH	138	Yes
Fridericia dichotoma	Bignoniaceae	ARR4	NA	194	Yes
Fridericia florida	Bignoniaceae	ARRF	ARRAFL	253	Yes
Fridericia patellifera	Bignoniaceae	ARRP	ARRAPA	239	Yes
Fridericia schumanniana	Bignoniaceae	ARRV	ARRAVE	24	Yes
Aspidosperma desmanthum	Apocynaceae	ASPC	ASPICR	30	Yes
Astronium graveolens	Anacardiaceae	ASTG	AST2GR	1160	Yes
Astrocaryum standleyanum	Arecaceae	ASTS	AST1ST	283	Yes
Attalea rostrata	Arecaceae	ATTB	SCH1ZO	404	Yes
Bactris coloniata	Arecaceae	BAC1	BACTC1	24	Yes
Bactris coloradensis	Arecaceae	BAC2	BACTC2	803	Yes

Bactris maraja	Arecaceae	BAC5	BACTM1	33	Yes
Bactris major	Arecaceae	BACM	BACTMA	183	Yes
Bauhinia purpurea	Fabaceae	BAU4	NA	42	Yes
Bauhinia guianensis	Fabaceae	BAUG	BAUHGU	91	Yes
Bauhinia reflexa	Fabaceae	BAUR	BAUHRE	26	Yes
Beilschmiedia tovarensis	Lauraceae	BEIP	BEILPE	1986	Yes
Pochota quinata	Bombacaceae	BOMQ	POCHQU	59	Yes
Pachira sessilis	Malvaceae	BOMS	POCHSE	67	Yes
Brosimum alicastrum	Moraceae	BROA	BROSAL	562	Yes
Bucida buceras	Combretaceae	BUCB	BUCIBU	18	No
Buchenavia tetraphylla	Combretaceae	BUCT	BUCHCA	163	No
Bunchosia nitida	Malpighiaceae	BUNC	BUNCCO	46	No
Bursera simaruba	Burseraceae	BURS	BURSSI	2023	No
Byrsonima crassifolia	Malpighiaceae	BYRC	BYRSCR	74	No
Canavalia gladiata	Fabaceae	CAGL	NA	15	No
Callichlamys latifolia	Bignoniaceae	CAL1	CALLLA	473	Yes
Calophyllum longifolium	Clusiaceae	CAL2	CALOLO	996	Yes
Calathea inocephala	Marantaceae	CALI	NA	54	No
Canavalia dictyota	Fabaceae	CAND	NA	22	No
Capparidastrum frondosa	Capparaceae	CAPF	CAPPFR	144	Yes
Vasconcellea cauliflora	Caricaceae	CARC	CARICA	6	No
Casearia aculeata	Salicaceae	CAS1	CASEAC	121	Yes
Casearia arborea	Salicaceae	CAS2	CASEAR	1475	Yes
Cassipourea elliptica	Rhizophoraceae	CAS3	CASSEL	301	Yes
Castilla elastica	Moraceae	CAS4	CASTEL	7	No
Casearia commersoniana	Salicaceae	CASC	CASECO	608	Yes
Casearia guianensis	Salicaceae	CASG	CASEGU	103	Yes
Cavanillesia platanifolia	Malvaceae	CAVP	CAVAPL	294	Yes
Cayaponia granatensis	Cucurbitaceae	CAYG	CAYAGR	10	Yes
Cecropia insignis	Urticaceae	CECI	CECRIN	185	Yes
Cecropia obtusifolia	Urticaceae	CECO	CECROB	196	Yes
Cecropia peltata	Urticaceae	CECP	CECRPE	139	Yes

Cedrela odorata	Meliaceae	CEDO	CEDROD	104	Yes
Ceiba pentandra	Malvaceae	CEIP	CEIBPE	580	Yes
Celtis iguanaea	Cannabaceae	CELI	CELTIG	283	Yes
Celtis schippii	Cannabaceae	CELS	CELTSC	360	Yes
Tanaecium tetragonolobum	Bignoniaceae	CERT	CERATE	452	Yes
Chamaedorea tepejilote	Arecaceae	CHAW	CHA1TE	603	Yes
Chomelia psilocarpa	Rubiaceae	CHOP	CHOMPS	8	Yes
Chondrodendron tomentosum	Menispermaceae	CHOT	CHONTO	81	Yes
Chrysophyllum argenteum	Sapotaceae	CHRA	CHR2AR	397	Yes
Chrysophyllum cainito	Sapotaceae	CHRC	CHR2CA	410	Yes
Cissus erosa	Vitaceae	CISE	CISSER	472	Yes
Cissus verticillata	Vitaceae	CISS	CISSSI	64	Yes
Clitoria javitensis	Fabaceae	CLIJ	CLITJA	36	Yes
Clidemia octona	Melastomataceae	CLIO	CLIDOC	122	Yes
Clidemia septuplinervia	Melastomataceae	CLIS	CLIDSE	153	Yes
Clusia minor	Clusiaceae	CLUM	NA	4	No
Clusia peninsularis	Clusiaceae	CLUP	NA	113	No
Clusia uvitana	Clusiaceae	CLUU	NA	444	No
Cnestidium rufescens	Connaraceae	CNER	CNESRU	1121	Yes
Coccoloba aculeata	Polygonaceae	COC2	COCCA2	145	Yes
Coccoloba coronata	Polygonaceae	COCC	COCCCO	1420	Yes
Coccoloba manzinellensis	Polygonaceae	COCM	COCCMA	2908	Yes
Coccoloba excelsa	Polygonaceae	COCP	COCCP1	671	Yes
Cochlospermum vitifolium	Bixaceae	COCV	COCHVI	1	No
Colubrina glandulosa	Rhamnaceae	COLG	COLUGL	215	No
Combretum cacoucia	Combretaceae	COMC	COMBCA	45	Yes
Combretum decandrum	Combretaceae	COMD	COMBDE	618	Yes
Combretum fruticosum	Combretaceae	COMF	COMBFR	719	Yes
Combretum laxum	Combretaceae	COML	COMBLA	348	Yes
Conostegia cinnamomea	Melastomataceae	CONC	CONOCI	335	Yes
Connarus panamensis	Connaraceae	CONP	CONNPA	559	Yes
Connarus turczaninowii	Connaraceae	CONT	CONNTU	434	Yes

Copaifera aromatica	Fabaceae	COPA	COPAAR	23	No
Cordia alliodora	Cordiaceae	CORA	CORDAL	2630	Yes
Cordia bicolor	Cordiaceae	CORB	CORDBI	1684	Yes
Cordia lasiocalyx	Cordiaceae	CORL	CORDLA	335	Yes
Cordia panamensis	Boraginaceae	CORP	CORDPA	280	Yes
Cosmibuena grandiflora	Rubiaceae	COSS	COSMGR	4	No
Coussarea curvigemmia	Rubiaceae	COUC	COU2CU	2273	Yes
Coutarea hexandra	Rubiaceae	COUH	COUTHE	31	Yes
Couratari guianensis	Lecythidaceae	COUP	COURPA	5	No
Mosannona garwoodii	Annonaceae	CRES	MALMSP	99	Yes
Croton billbergianus	Euphorbiaceae	CROB	CROTBI	803	Yes
Cuervea kappleriana	Celastraceae	CUEK	CUERKA	41	Yes
Cupania latifolia	Sapindaceae	CUPL	CUPALA	507	Yes
Cupania rufescens	Sapindaceae	CUPR	CUPARU	46	Yes
Cupania seemannii	Sapindaceae	CUPS	CUPASY	746	Yes
Bignonia aequinoctialis	Bignoniaceae	CYDA	CYDIAE	235	Yes
Solanum circinatum	Solanaceae	CYPH	CYPHHA	2	Yes
Dalechampia dioscoreifolia	Euphorbiaceae	DALD	DALEDI	12	Yes
Davilla nitida	Dilleniaceae	DAVN	DAVINI	983	Yes
Dendropanax arboreus	Araliaceae	DENA	DENDAR	2239	Yes
Desmodium axillare	Fabaceae	DESA	DESMAX	206	Yes
Desmodium incanum	Fabaceae	DESC	NA	32	Yes
Desmoncus orthacanthos	Arecaceae	DESI	DES1OR	514	Yes
Desmopsis panamensis	Annonaceae	DESP	DES2PA	298	Yes
Dichapetalum gentryi	Dichapetalaceae	DICG	NA	3	No
Diospyros artanthifolia	Ebenaceae	DIO2	DIO2AR	11	Yes
Dioclea wilsonii	Fabaceae	DIOW	DIOCWI	85	Yes
Dipteryx oleifera	Fabaceae	DIPP	DIPTPA	607	Yes
Doliocarpus major	Dilleniaceae	DOL1	DOLIMA	940	Yes
Doliocarpus multiflorus	Dilleniaceae	DOL2	DOLIMU	669	Yes
Doliocarpus dentatus	Dilleniaceae	DOLD	DOLIDE	2	Yes
Doliocarpus olivaceus	Dilleniaceae	DOLO	DOLIOL	842	Yes

Drypetes standleyi	Putranjivaceae	DRYS	DRYPST	289	Yes
Enterolobium cyclocarpum	Fabaceae	ENTC	ENTECY	19	Yes
Entada rhedii	Fabaceae	ENTM	ENTAMO	76	Yes
Enterolobium schomburgkii	Fabaceae	ENTS	ENTESC	180	Yes
Epiphyllum phyllanthus var.rubrocoronatum	Cactaceae	EPPR	NA	3	No
Erythrina costaricensis	Fabaceae	ERYC	ERY1CO	2	Yes
Eugenia coloradoensis	Myrtaceae	EUGC	EUGECO	464	Yes
Eugenia galalonensis	Myrtaceae	EUGG	EUGEGA	84	Yes
Eugenia nesiotica	Myrtaceae	EUGN	EUGENE	278	Yes
Eugenia oerstediana	Myrtaceae	EUGO	EUGEOE	152	Yes
Eugenia venezuelensis	Myrtaceae	EUGV	EUGEVE	508	Yes
Faramea luteovirens	Rubiaceae	FARL	FARALU	146	Yes
Faramea occidentalis	Rubiaceae	FARO	FARAOC	3085	Yes
Fevillea cordifolia	Cucurbitaceae	FEVC	FEVICO	36	Yes
Ficus colubrinae	Moraceae	FIC1	FICUC1	273	Yes
Ficus costaricana	Moraceae	FIC2	FICUC2	17	Yes
Ficus citrifolia	Moraceae	FICI	FICUCI	1497	Yes
Ficus insipida	Moraceae	FIIN	FICUIN	519	Yes
Ficus maxima	Moraceae	FIMA	FICUMA	56	Yes
Ficus nymphaeifolia	Moraceae	FINY	FICUNY	126	Yes
Ficus obtusifolia	Moraceae	FIOB	FICUOB	107	Yes
Ficus pertusa	Moraceae	FIP2	FICUPE	861	Yes
Ficus paraensis	Moraceae	FIPA	FICUPA	32	Yes
Ficus popenoei	Moraceae	FIPO	FICUPO	269	Yes
Fischeria blepharopetala	Apocynaceae	FISF	FISCBL	5	No
Ficus tonduzii	Moraceae	FITO	FICUTO	25	Yes
Ficus trigonata	Moraceae	FITR	FICUTR	219	Yes
Ficus yoponensis	Moraceae	FIYO	FICUYO	64	Yes
Forsteronia acouci	Apocynaceae	FORV	FORSVI	129	Yes
Geonoma congesta	Arecaceae	GEC2	GEONCO	120	No
Genipa americana	Rubiaceae	GENA	GENIAM	59	Yes
Geonoma cuneata_procumbens	Arecaceae	GEOC	GEONCU	144	No

Geophila repens	Rubiaceae	GEOR	NA	21	No
Gnetum leyboldii	Gnetaceae	GNEL	GNETLE	140	Yes
Gouania colombiana	Rhamnaceae	GOUC	GOUACO	62	Yes
Gouania lupuloides	Rhamnaceae	GOUL	GOUALU	723	Yes
Gouania polygama	Rhamnaceae	GOUP	GOUAPO	135	Yes
Guarea grandifolia	Meliaceae	GUA1	GUARGR	636	Yes
Guarea guidonia	Meliaceae	GUA2	GUARGU	443	Yes
Guatteria amplifolia	Annonaceae	GUAA	GUATAM	266	Yes
Guatteria lucens	Annonaceae	GUAD	GUATDU	778	Yes
Guapira standleyana	Nyctaginaceae	GUAS	GUAPST	1785	Yes
Guazuma ulmifolia	Malvaceae	GUAU	GUAZUL	320	Yes
Guettarda foliacea	Rubiaceae	GUEF	GUETFO	527	Yes
Gustavia superba	Lecythidaceae	GUSS	GUSTSU	602	Yes
Hamelia axillaris	Rubiaceae	HAM1	HAMEAX	96	Yes
Hamelia patens	Rubiaceae	HAMP	HAMEPA	38	Yes
Hasseltia floribunda	Salicaceae	HASF	HASSFL	331	Yes
Clusia flavida	Clusiaceae	HAVF	HAVEFL	190	No
Heisteria acuminata	Erythropalaceae	HEIA	HEISAC	429	Yes
Heisteria concinna	Erythropalaceae	HEIC	HEISCO	879	Yes
Heliconia platystachys	Heliconiaceae	HEL1	NA	55	No
Henriettea succosa	Melastomataceae	HENS	HENRSU	68	No
Herrania purpurea	Malvaceae	HERP	HERRPU	35	Yes
Heteropteris laurifolia	Malpighiaceae	HETL	HETELA	353	Yes
Hippocratea volubilis	Celastraceae	HIPV	HIPPVO	575	Yes
Hiraea reclinata	Malpighiaceae	HIR1	HIRARE	650	Yes
Hiraea fagifolia	Malpighiaceae	HIR3	HIRAF1	22	Yes
Hirtella americana	Chrysobalanaceae	HIRA	HIRTAM	73	Yes
Hiraea faginea	Malpighiaceae	HIRF	HIRAFA	184	Yes
Hiraea grandifolia	Malpighiaceae	HIRG	HIRAGR	152	Yes
Hiraea smilacina	Malpighiaceae	HIRQ	HIRAQU	152	Yes
Hirtella triandra	Chrysobalanaceae	HIRT	HIRTTR	181	Yes
Hura crepitans	Euphorbiaceae	HURC	HURACR	23	Yes

Pombalia prunifolia	Violaceae	HYBP	HYBAPR	694	Yes
Hieronyma alchorneoides	Euphorbiaceae	HYEL	HYERAL	7480	Yes
Hylenaea praecelsa	Celastraceae	HYLP	HYLEPR	466	Yes
Hymenaea courbaril	Fabaceae	HYMC	HYMECO	89	No
Inga acuminata	Fabaceae	INA1	INGAS1	32	Yes
Inga cocleensis	Fabaceae	INCO	INGACO	42	Yes
Inga laurina	Fabaceae	INFA	INGARU	505	Yes
Inga alba	Fabaceae	INGAAL	INGAAL	2	Yes
Inga goldmanii	Fabaceae	INGO	INGAGO	11	Yes
Inga mucuna	Fabaceae	INM1	INGAM1	19	Yes
Inga multijuga	Fabaceae	INM2	INGAM2	73	Yes
Inga marginata	Fabaceae	INMA	INGAMA	168	Yes
Inga oersterdiana	Fabaceae	INMI	INGAMI	24	Yes
Inga pauciflora	Fabaceae	INPA	INGAPA	120	Yes
Inga pezizifera	Fabaceae	INPE	INGAPE	35	Yes
Inga punctata	Fabaceae	INPU	INGAPU	315	Yes
Inga nobilis	Fabaceae	INQU	INGAQU	13	Yes
Inga ruiziana	Fabaceae	INRU	INGARU	17	Yes
Inga sapindoides	Fabaceae	INSA	INGASA	305	Yes
Inga thibaudiana	Fabaceae	INTH	INGATH	39	Yes
Inga umbellifera	Fabaceae	INUM	INGAUM	39	Yes
Jacaranda copaia	Bignoniaceae	JACC	JAC1CO	626	Yes
Jacaratia spinosa	Caricaceae	JACS	JAC2SP	2	No
Lacistema aggregatum	Lacistemataceae	LACA	LACIAG	1019	Yes
Lacmellea panamensis	Apocynaceae	LACP	LACMPA	783	Yes
Laetia procera	Salicaceae	LAEP	LAETPR	78	Yes
Laetia thamnia	Salicaceae	LAET	LAETTH	232	Yes
Lafoensia punicifolia	Lythraceae	LAFP	LAFOPU	312	Yes
Lagerstroemia speciosa	Lythraceae	LAGS	LAGESP	61	No
Lasiacis maculata	Poaceae	LASS	NA	690	No
Leandra dichotoma	Melastomataceae	LEAD	LEANDI	842	Yes
Licania hypoleuca	Chrysobalanaceae	LICH	LICAHY	2	Yes

Licania platypus	Chrysobalanaceae	LICP	LICAPL	627	Yes
Lindackeria laurina	Achariaceae	LINL	LINDLA	902	Yes
Lonchocarpus luteomaculatus	Fabaceae	LON1	NA	358	Yes
Lonchocarpus ferrugineus	Fabaceae	LONF	LONCFE	77	Yes
Lonchocarpus heptaphyllus	Fabaceae	LONL	LONCLA	654	Yes
Lozania pittieri	Lacistemataceae	LOZP	LOZAPI	30	Yes
Luehea seemannii	Malvaceae	LUE1	LUEHSE	778	Yes
Lycianthes maxonii	Solanaceae	LYCM	LYCIMA	48	Yes
Mabea occidentalis	Euphorbiaceae	MABO	MABEOC	750	No
Machaerium microphyllum	Fabaceae	MAC1	MACHM1	142	Yes
Machaerium milleflorum	Fabaceae	MAC2	MACHM2	443	Yes
Machaerium arboreum	Fabaceae	MACA	MACHAR	279	Yes
Macrocnemum roseum	Rubiaceae	MACG	MACRGL	1741	Yes
Machaerium kegelii	Fabaceae	MACK	MACHKE	295	Yes
Machaerium seemannii	Fabaceae	MACS	MACHSE	614	Yes
Dolichandra unguis-cati	Bignoniaceae	MACU	MACFUN	56	Yes
Mangifera indica	Anacardiaceae	MANI	MANGIN	1	No
Maquira guianensis	Moraceae	MAQC	MAQUCO	17	Yes
Margaritaria nobilis	Phyllanthaceae	MAR2	MARGNO	199	No
Martinella obovata	Bignoniaceae	MARO	MARTOB	13	Yes
Maripa panamensis	Convolvulaceae	MARP	MAR2PA	733	Yes
Markea panamensis	Solanaceae	MARU	MARKUL	8	Yes
Adelphia hiraea	Malpighiaceae	MASH	MASCHI	1094	Yes
Mascagnia divaricata	Malpighiaceae	MASN	MASCNE	1040	Yes
Mascagnia ovatifolia	Malpighiaceae	MASO	MASCNE	8	Yes
Matayba apetala	Sapindaceae	MATA	MATAAP	50	No
Maytenus schippii	Celastraceae	MAYS	MAYTSC	38	Yes
Mendoncia gracilis	Acanthaceae	MENG	MENDGR	116	Yes
Mendoncia retusa	Acanthaceae	MENL	MENDLI	67	Yes
Mesechites trifidus	Apocynaceae	MEST	MESETR	97	Yes
Miconia affinis	Melastomataceae	MIC1	MICOAF	481	Yes
Miconia argentea	Melastomataceae	MIC2	MICOAR	1446	Yes

Miconia nervosa	Melastomataceae	MICN	MICONE	47	Yes
Mikania leiostachya	Compositae	MIKL	MIKALE	10400	Yes
Mimosa pigra	Fabaceae	MIMP	NA	16	No
Monstera dubia	Araceae	MODU	MONSDU	25	No
Mouriri myrtilloides	Melastomataceae	MOUM	MOURMY	817	Yes
Mucuna mutisiana	Fabaceae	MUCM	MUCUMU	2	Yes
Myrospermum frutescens	Fabaceae	MYR2	MYROFR	14	Yes
Myroxylum balsamum	Fabaceae	MYRB	MYR3BA	16	No
Myrcia splendens_tip._gatunensis	Myrtaceae	MYRG	MYRCGA	796	Yes
Myrcia zetekiana	Myrtaceae	MYRZ	MYRCZE	16	Yes
Nectandra cissiflora	Lauraceae	NECC	NECTCI	291	Yes
Nectandra lineata	Lauraceae	NECL	NECTGL	413	Yes
Damburneya umbrosa	Lauraceae	NECP	NECTPU	96	No
Neea amplifolia	Nyctaginaceae	NEEA	NEEAAM	219	Yes
Ochroma pyramidale	Malvaceae	OCHP	OCHRPY	413	Yes
Ocotea cernua	Lauraceae	OCOC	OCOTCE	37	Yes
Ocotea oblonga	Lauraceae	OCOO	OCOTOB	116	Yes
Ocotea puberula	Lauraceae	OCOP	OCOTPU	958	Yes
Ocotea whitei	Lauraceae	OCOS	OCOTWH	60	Yes
Odontocarya tamoides	Menispermaceae	ODO1	ODO2TA	88	Yes
Odontocarya truncata	Menispermaceae	ODO2	ODO2TR	149	Yes
Odontadenia macrantha	Apocynaceae	ODOM	ODO1MA	343	Yes
Oenocarpus mapora	Arecaceae	OENM	OENOMA	2362	Yes
Trophis caucana	Moraceae	OLMA	OLMEAS	8	Yes
Omphalea diandra	Euphorbiaceae	OMPD	OMPHDI	38	Yes
Ormosia amazonica	Fabaceae	ORMA	ORMOAM	300	Yes
Ormosia coccinea	Fabaceae	ORMC	ORMOCR	30	Yes
Ormosia macrocalyx	Fabaceae	ORMM	ORMOMA	294	Yes
Ormosia panamensis	Fabaceae	ORMP	NA	3	Yes
Ouratea lucens	Ochnaceae	OURL	OURALU	270	Yes
Pachira aquatica	Malvaceae	PACA	POCHAQ	29	Yes
Pachyptera kerere	Bignoniaceae	PACK	MANSKE	1	Yes

Palicourea guianensis	Rubiaceae	PALG	PALIGU	1952	Yes
Parmentiera ceraifera	Bignoniaceae	PARC	PARMCE	1	No
Tanaecium pyramidatum	Bignoniaceae	PARP	PAR1PY	43	Yes
Passiflora ambigua	Passifloraceae	PAS1	PASSAM	53	Yes
Passiflora auriculata	Passifloraceae	PAS2	PASSAU	62	Yes
Passiflora seemannii	Passifloraceae	PASS	PASSSE	15	Yes
Paullinia baileyi	Sapindaceae	PAU1	PAULBA	294	Yes
Paullinia fuscescens	Sapindaceae	PAU3	PAULFU	67	Yes
Paullinia glomerulosa	Sapindaceae	PAU4	PAULG2	57	Yes
Paullinia pinnata	Sapindaceae	PAU5	PAULPI	577	Yes
Paullinia pterocarpa	Sapindaceae	PAU6	PAULPT	95	Yes
Paullinia fibrigera	Sapindaceae	PAUF	PAULFI	38	Yes
Paullinia rugosa	Sapindaceae	PAUR	PAULRU	435	Yes
Paullinia turbacensis	Sapindaceae	PAUT	PAULTU	228	Yes
Pentagonia macrophylla	Rubiaceae	PENM	PENTMA	60	Yes
Pera arborea	Peraceae	PERA	PERAAR	129	No
Petrea volubilis	Verbenaceae	PETA	PETRAS	336	Yes
Cinnamomum triplinerve	Lauraceae	PHOM	PHOECI	651	Yes
Bignonia corymbosa	Bignoniaceae	PHRC	PHRYCO	9	Yes
Picramnia latifolia	Picramniaceae	PICL	PICRLA	701	Yes
Amphilophium crucigerum	Bignoniaceae	PITC	PIT2CR	410	Yes
Cojoba rufescens	Fabaceae	PITR	PIT1RU	208	Yes
Platypodium elegans	Fabaceae	PLAE	PLA2EL	1209	Yes
Platymiscium pinnatum	Fabaceae	PLAP	PLA1PI	855	Yes
Posoqueria latifolia	Rubiaceae	POSL	POSOLA	245	Yes
Pouteria stipitata	Sapotaceae	POU2	POUTST	228	Yes
Poulsenia armata	Moraceae	POUA	POULAR	151	Yes
Pourouma bicolor	Urticaceae	POUB	POURBI	749	Yes
Pouteria fossicola	Sapotaceae	POUF	POUTFO	13	Yes
Pouteria reticulata	Sapotaceae	POUU	POUTRE	941	Yes
Prestonia portobellensis	Apocynaceae	PREP	PRESPO	62	Yes
Prionostemma aspera	Celastraceae	PRIA	PRI1AS	354	Yes

Prioria copaifera	Fabaceae	PRIC	PRI2CO	352	Yes
Protium costaricense	Burseraceae	PROC	PROTCO	621	Yes
Protium panamense	Burseraceae	PROP	PROTPA	1284	Yes
Protium tenuifolium	Burseraceae	PROT	PROTTE	1516	Yes
Pseudobombax septenatum	Malvaceae	PSE1	PSE1SE	75	Yes
Psidium guineense	Myrtaceae	PSI1	PSIDG1	26	Yes
Chamguava schippii	Myrtaceae	PSIA	CHA2SC	2	Yes
Psiguria triphylla	Cucurbitaceae	PSIB	PSIGBI	7	Yes
Psidium friedrichsthalianum	Myrtaceae	PSIF	PSIDFR	51	Yes
Psiguria warscewiczii	Cucurbitaceae	PSIW	PSIGWA	3	Yes
Pterocarpus hayesii	Fabaceae	PTER	PTERRO	446	Yes
Palicourea acuminata	Rubiaceae	PYAC	PSYCAC	428	Yes
Psychotria gracilenta	Rubiaceae	PYB2	PSYCB2	384	Yes
Psychotria capitata	Rubiaceae	PYC1	PSYCC1	380	Yes
Psychotria chagrensis	Rubiaceae	PYCH	PSYCCH	45	Yes
Psychotria longicuspis	Rubiaceae	PYCI	PSYCCI	223	Yes
Palicourea deflexa	Rubiaceae	PYDE	PSYCDE	973	Yes
Ronabea emetica	Rubiaceae	PYEM	PSYCEM	154	Yes
Ronabea latifolia	Rubiaceae	PYER	PSYCER	107	Yes
Palicourea hoffmannseggiana	Rubiaceae	PYFU	PSYCFU	264	Yes
Psychotria grandis	Rubiaceae	PYG3	PSYCG3	657	Yes
Psychotria horizontalis	Rubiaceae	PYHO	PSYCHO	744	Yes
Carapichea ipecacuanha	Rubiaceae	PYIP	PSYCIP	80	Yes
Psychotria limonensis	Rubiaceae	PYLI	PSYCLI	1750	Yes
Psychotria marginata	Rubiaceae	PYMA	PSYCMA	1184	Yes
Psychotria micrantha	Rubiaceae	PYMI	PSYCMI	1335	Yes
Palicourea cyanococca	Rubiaceae	PYPI	PSYCPI	50	Yes
Palicourea racemosa	Rubiaceae	PYRA	PSYCRA	825	Yes
Quararibea asterolepis	Malvaceae	QUA1	QUARAS	2872	Yes
Quassia amara	Simaroubaceae	QUA2	QUASAM	1	Yes
Quararibea pterocalyx	Malvaceae	QUAP	QUARPT	23	Yes
Randia armata	Rubiaceae	RANA	RANDAR	242	Yes

Renealmia alpinia	Zingiberaceae	RENA	NA	3	No
Garcinia madruno	Clusiaceae	RHEA	GAR2MA	50	Yes
Garcinia recondita	Clusiaceae	RHEE	GAR2IN	1264	Yes
Rhynchosia pyramidalis	Fabaceae	RHYP	RHYCPY	11	Yes
Rinorea squamata	Violaceae	RIN1	RINOSQ	54	Yes
Rinorea sylvatica	Violaceae	RIN2	RINOSY	599	Yes
Rourea adenophora	Connaraceae	ROUA	NA	55	Yes
Rourea glabra	Connaraceae	ROUG	ROURGL	19	Yes
Saccharum spontaneum	Poaceae	SACS	SACCSP	13	No
Sapium glandulosum	Euphorbiaceae	SAPG	SAPIAU	175	Yes
Schefflera morototoni	Araliaceae	SCHM	SCH2MO	480	No
Schizolobium parahyba	Fabaceae	SCHP	SCHIPA	18	Yes
Securidaca diversifolia	Polygalaceae	SECD	SECUDI	50	Yes
Senna dariensis	Fabaceae	SEND	SENNDA	67	Yes
Senna reticulata	Fabaceae	SENR	SENNRE	252	Yes
Senna undulata	Fabaceae	SENU	SENNUN	138	Yes
Serjania circumvallata	Sapindaceae	SER1	SERJCI	409	Yes
Serjania cornigera	Sapindaceae	SER2	SERJCO	149	Yes
Serjania paucidentata	Sapindaceae	SER3	SERJPA	320	Yes
Serjania pluvialiflorens	Sapindaceae	SER4	SERJPL	158	Yes
Serjania tenuifolia	Sapindaceae	SER5	NA	65	Yes
Serjania atrolineata	Sapindaceae	SERA	SERJAT	2	Yes
Serjania decapleuria	Sapindaceae	SERD	SERJDE	541	Yes
Serjania mexicana	Sapindaceae	SERM	SERJME	1234	Yes
Serjania trachygona	Sapindaceae	SERT	SERJTR	205	Yes
Simarouba amara	Simaroubaceae	SIMA	SIMAAM	1359	Yes
Siparuna guianensis	Siparunaceae	SIP2	SIPAGU	41	Yes
Siparuna cristata	Siparunaceae	SIPG	SIPAGU	19	Yes
Siparuna pauciflora	Siparunaceae	SIPP	SIPAPA	67	Yes
Sloanea terniflora	Elaeocarpaceae	SLOT	SLOATE	442	Yes
Smilax domingensis	Smilacaceae	SMIL	SMILLA	597	Yes
Smilax mollis	Smilacaceae	SMIM	SMILMO	522	Yes

Smilax purhampuy	Smilacaceae	SMIP	SMILPA	146	Yes
Socratea exorrhiza	Arecaceae	SOCE	SOCREX	792	Yes
Solanum asperum	Solanaceae	SOL4	SOLAAS	18	Yes
Solanum lanceifolium	Solanaceae	SOL5	SOLALA	127	Yes
Solanum hayesii	Solanaceae	SOLH	SOLAHA	172	Yes
Solanum jamaicense	Solanaceae	SOLJ	SOLAJA	101	Yes
Solanum adhaerens	Solanaceae	SOLL	NA	15	Yes
Sorocea affinis	Moraceae	SORA	SOROAF	1910	Yes
Souroubea sympetala	Marcgraviaceae	SOUS	SOURSY	232	Yes
Spachea membranacea	Malpighiaceae	SPAM	SPACME	650	Yes
Spondias mombin	Anacardiaceae	SPOM	SPONMO	1112	Yes
Spondias radlkoferi	Anacardiaceae	SPOR	SPONRA	1078	Yes
Sterculia apetala	Malvaceae	STEA	STERAP	142	Yes
Tabernaemontana grandiflora	Apocynaceae	STEG	STEMGR	28	Yes
Stigmaphyllon hypargyreum	Malpighiaceae	STIH	STIGHY	1	Yes
Stigmaphyllon lindenianum	Malpighiaceae	STIL	STIGLI	262	Yes
Strychnos brachistanta	Loganiaceae	STRB	STRYBR	18	Yes
Strychnos bredemeyeri	Loganiaceae	STRD	STRYDA	30	Yes
Strychnos panamensis	Loganiaceae	STRP	STRYPA	138	Yes
Strychnos toxifera	Loganiaceae	STRT	STRYTO	6	Yes
Stylogyne turbacensis	Myrsinaceae	STYS	STYLST	42	Yes
Swartzia simplex_var.grandiflora	Fabaceae	SWA1	SWARS1	126	Yes
Swartzia simplex_var.continentalis	Fabaceae	SWA2	SWARS2	367	Yes
Symphonia globulifera	Clusiaceae	SYMG	SYMPGL	117	Yes
Syngonium podophyllum	Araceae	SYNP	NA	1	No
Tabernaemontana arborea	Apocynaceae	TABA	TAB2AR	88	Yes
Handroanthus guayacan	Bignoniaceae	TABG	TAB1GU	476	Yes
Tabernaemontana panamensis	Apocynaceae	TABP	TAB2PA	1	Yes
Tabebuia rosea	Bignoniaceae	TABR	TAB1RO	1082	Yes
Tachigali panamensis	Fabaceae	TACV	TACHVE	221	Yes
Talisia nervosa	Sapindaceae	TALN	TALINE	835	Yes
Terminalia amazonia	Combretaceae	TERA	TERMAM	2006	Yes

Terminalia oblonga	Combretaceae	TERO	TERMOB	838	Yes
Tetracera portobellensis	Dilleniaceae	TET1	TET1PO	433	Yes
Tetragastris panamensis	Burseraceae	TET2	TET2PA	1024	Yes
Tetrapterys discolor	Malpighiaceae	TETD	TET3DI	399	Yes
Tetracera hydrophila	Dilleniaceae	TETH	TET1HY	558	Yes
Tetrathylacium johansenii	Salicaceae	TETJ	TET4JO	239	Yes
Tetrapterys goudotiana	Malpighiaceae	TETM	TET3MA	379	Yes
Tetracera volubilis	Dilleniaceae	TETV	TET1VO	195	Yes
Thevetia ahouai	Apocynaceae	THEA	THEVAH	23	Yes
Theobroma cacao	Malvaceae	THEC	THEOCA	14	Yes
Thinouia myriantha	Sapindaceae	THIM	THINMY	697	Yes
Tocoyena pittieri	Rubiaceae	TOCP	TOCOPI	146	Yes
Tontelea ovalifolia	Celastraceae	TONO	TONTOV	111	Yes
Topobea parasitica	Melastomataceae	TOPP	NA	66	No
Tovomita longifolia	Clusiaceae	TOVL	TOVOLO	28	Yes
Chrysochlamys eclipes	Clusiaceae	TOVN	CHR1EC	140	Yes
Tovomita stylosa	Clusiaceae	TOVS	TOVOST	8	Yes
Trattinnickia aspera	Burseraceae	TRAA	TRATAS	1064	Yes
Trema interregima	Cannabaceae	TREI	TREMIN	78	Yes
Trema micrantha	Cannabaceae	TREM	TREMMI	873	Yes
Trichilia pallida	Meliaceae	TRI1	TRI2PA	431	Yes
Trichilia pleeana	Meliaceae	TRI2	TRI2PL	71	Yes
Trichilia tuberculata	Meliaceae	TRI3	TRI2TU	6810	Yes
Trichospermum galeottii	Malvaceae	TRI6	TRI4GA	209	No
Triplaris cumingiana	Polygonaceae	TRIC	TRIPCU	1139	Yes
Trichanthera gigantea	Acanthaceae	TRIG	TRI1GI	72	Yes
Trichilia hirta	Meliaceae	TRIH	TRI2HI	546	Yes
Trophis racemosa	Moraceae	TROR	TROPRA	303	Yes
Turpinia occidentalis	Tapisciaceae	TURO	TURPOC	441	Yes
Tynnanthus croatianus	Bignoniaceae	TYNC	TYNNCR	249	Yes
Unonopsis pittieri	Annonaceae	UNOP	UNONPI	280	Yes
Vatairea erythrocarpa	Fabaceae	VATE	VATAER	123	No

Virola sebifera	Myristicaceae	VIR1	VIROSE	679	Yes
Virola nobilis	Myristicaceae	VIR2	VIROSU	1061	Yes
Virola multiflora	Myristicaceae	VIR3	VIROSP	65	Yes
Vismia macrophylla	Clusiaceae	VISM	VISMMA	41	Yes
Vitis tiliifolia	Vitaceae	VITT	VITITI	1956	No
Vochysia ferruginea	Vochysiaceae	VOCF	VOCHFE	94	Yes
Witheringia solanacea	Solanaceae	WITS	WITHSO	50	No
Xylopia macrantha	Annonaceae	XYLM	XYL1MA	20	Yes
Zanthoxylum panamense	Rutaceae	ZAN1	ZANTP1	254	Yes
Zanthoxylum acuminatum	Rutaceae	ZAN2	ZANTPR	2362	Yes
Zanthoxylum ekmanii	Rutaceae	ZANB	ZANTBE	76	Yes
Zuelania guidonia	Salicaceae	ZUEG	ZUELGU	166	Yes
Zygia latifolia	Fabaceae	ZYGL	ZYGILA	79	No

6 Table S2: List of documented interactions between plant species and seed predator species

A list of the 471 documented interactions between plant species and insect seed predator species documented in our study. The 'Count' column indicates how many times each plant species × seed predator species interaction was observed. The species codes used for seed predator species that could not be assigned to known species correspond to codes in S. Gripenberg's seed predator reference collection (kept at the Smithsonian Tropical Research Institute; Panama). For insect species that were successfully barcoded, the Barcode Index Number (BIN) is given. Interactions are sorted according to plant species, with plant species listed in alphabetical order.

Insect species	Insect family	Insect order	BIN	Host species	Host family	Count
Stator aegrotus	Chrysomelidae	Coleoptera	BOLD:AAJ5083	Acacia hayesii	Fabaceae	40
Stator monachus	Chrysomelidae	Coleoptera		Acacia hayesii	Fabaceae	2
Stator trisignatus	Chrysomelidae	Coleoptera	BOLD:AAH9942	Acacia hayesii	Fabaceae	2
Pyra lep327SG	Pyralidae	Lepidoptera	BOLD:ACG0975	Acacia polyphylla	Fabaceae	1
Stator monachus	Chrysomelidae	Coleoptera		Acacia polyphylla	Fabaceae	2
Stator trisignatus	Chrysomelidae	Coleoptera	BOLD:AAH9942	Acacia polyphylla	Fabaceae	12
Anthonomus sp. cur133SG	Curculionidae	Coleoptera	BOLD:ABV0303	Adelphia hiraea	Malpighiaceae	2
Apogeshna stenialisDHJ02	Crambidae	Lepidoptera	BOLD:AAA0336	Adelphia hiraea	Malpighiaceae	1
Bothryopteron sp. cur85SG	Brentidae	Coleoptera	BOLD:ABU8658	Adelphia hiraea	Malpighiaceae	333
Gele sp. lep326SG	Gelechiidae	Lepidoptera	BOLD:ACG2471	Adelphia hiraea	Malpighiaceae	2
Merobruchus sp. bru3SG	Chrysomelidae	Coleoptera		Albizia sp.	Fabaceae	5
Cosm sp. lep299SG	Cosmopterigidae	Lepidoptera	BOLD:AAH5906	Alchornea costaricensis	Euphorbiaceae	37
Eurn sp. hym22SG	Eurytomidae	Hymenoptera		Alibertia edulis	Rubiaceae	18
Eurn sp. hym23SG	Eurytomidae	Hymenoptera		Alibertia edulis	Rubiaceae	19
Aeatus costulatus	Curculionidae	Coleoptera	BOLD:ABV0286	Amphilophium crucigerum	Bignoniaceae	84
Aeatus sp. cur242SG	Curculionidae	Coleoptera	BOLD:ABV0287	Amphilophium crucigerum	Bignoniaceae	3
Clydonopteron pomponius	Crambidae	Lepidoptera	BOLD:AAP2098	Amphilophium crucigerum	Bignoniaceae	6
Pyra sp. lep81SG	Pyralidae	Lepidoptera		Amphilophium crucigerum	Bignoniaceae	3
Aeatus costulatus	Curculionidae	Coleoptera	BOLD:ABV0286	Amphilophium paniculatum	Bignoniaceae	188

Insect species	Insect family	Insect order	BIN	Host species	Host family	Count
Aeatus sp. cur263SG	Curculionidae	Coleoptera	BOLD:ACS6695	Amphilophium paniculatum	Bignoniaceae	2
Curc sp. cur180SG	Curculionidae	Coleoptera	BOLD:ACJ5780	Amphilophium paniculatum	Bignoniaceae	2
Anacampsis phytomiella	Gelechiidae	Lepidoptera	BOLD:AAP2073	Anacardium excelsum	Anacardiaceae	13
Conotrachelus sp. cur249SG	Curculionidae	Coleoptera		Anacardium excelsum	Anacardiaceae	1
Conotrachelus sp. cur68SG	Curculionidae	Coleoptera	BOLD:ACG2730	Anacardium excelsum	Anacardiaceae	15
Gele sp. lep302SG	Gelechiidae	Lepidoptera	BOLD:ACJ4457	Anacardium excelsum	Anacardiaceae	1
Cerambycidae sp. colcer7SG	Cerambycidae	Coleoptera	BOLD:ACJ3952	Andira inermis	Fabaceae	3
Trichapion sp. cur101SG	Brentidae	Coleoptera	BOLD:ACG1965	Andira inermis	Fabaceae	31
Cerconota anonella	Oecophoridae	Lepidoptera	BOLD:ABV2177	Annona hayesii	Annonaceae	23
Cerconota anonella	Oecophoridae	Lepidoptera	BOLD:ABV2177	Annona spraguei	Annonaceae	23
Pyra sp. lep478SG	Pyralidae	Lepidoptera	BOLD:ABW7510	Anthodon panamense	Celastraceae	1
Amblycerus whiteheadi	Chrysomelidae	Coleoptera	BOLD:ABV3598	Apeiba membranacea	Malvaceae	71
Desmia bajulalis	Crambidae	Lepidoptera	BOLD:AAA0408	Apeiba membranacea	Malvaceae	11
Amblycerus whiteheadi	Chrysomelidae	Coleoptera	BOLD:ABV3598	Apeiba tibourbou	Malvaceae	21
Cosm sp. lep459SG	Cosmopterigidae	Lepidoptera		Apeiba tibourbou	Malvaceae	1
Cryptorhynchus sp. cur27SG	Curculionidae	Coleoptera		Apeiba tibourbou	Malvaceae	1
Desmia bajulalis	Crambidae	Lepidoptera	BOLD:AAA0408	Apeiba tibourbou	Malvaceae	4
Metamasius sp. cur134SG	Dryophthoridae	Coleoptera	BOLD:ACA8671	Astrocaryum standleyanum	Arecaceae	5
Pachymerus bactris	Chrysomelidae	Coleoptera	BOLD:ABV3600	Astrocaryum standleyanum	Arecaceae	42
Pachymerus sp. bru67SG	Chrysomelidae	Coleoptera	BOLD:ACL6561	Astrocaryum standleyanum	Arecaceae	2
Pseudobaris sp. cur145SG	Curculionidae	Coleoptera		Astrocaryum standleyanum	Arecaceae	16
Cosm sp. lep271SG	Cosmopterigidae	Lepidoptera	BOLD:ACF9942	Astronium graveolens	Anacardiaceae	11
Bruc sp. bru54SG	Chrysomelidae	Coleoptera	BOLD:ACJ4643	Attalea rostrata	Arecaceae	2
Bruc sp. bru66SG	Chrysomelidae	Coleoptera	BOLD:ACJ6014	Attalea rostrata	Arecaceae	2
Speciomerus giganteus	Chrysomelidae	Coleoptera	BOLD:ABV3599	Attalea rostrata	Arecaceae	32
Gele sp. lep3SG	Gelechiidae	Lepidoptera	BOLD:ABV2181	Bactris coloniata	Arecaceae	1
Pachymerus bactris	Chrysomelidae	Coleoptera	BOLD:ABV3600	Bactris major	Arecaceae	2

Insect species	Insect family	Insect order	BIN	Host species	Host family	Count
Pseudobaris sp. cur145SG	Curculionidae	Coleoptera	BOLD:ACG0001	Bactris major	Arecaceae	13
Bruc sp. bru38SG	Chrysomelidae	Coleoptera	BOLD:ACJ4487	Bauhinia guianensis	Fabaceae	14
Bruc sp. bru43SG	Chrysomelidae	Coleoptera	BOLD:ACJ4490	Bauhinia guianensis	Fabaceae	4
Bruc sp. bru49SG	Chrysomelidae	Coleoptera	BOLD:ACJ4489	Bauhinia guianensis	Fabaceae	10
Bruc sp. bru43SG	Chrysomelidae	Coleoptera	BOLD:ACJ4490	Bauhinia reflexa	Fabaceae	1
Bruc sp. bru49SG	Chrysomelidae	Coleoptera	BOLD:ACJ4489	Bauhinia reflexa	Fabaceae	2
Heilipus sp. cur243SG	Curculionidae	Coleoptera	BOLD:ABV0296	Beilschmiedia tovarensis	Lauraceae	2
Heilipus sp. cur50SG	Curculionidae	Coleoptera	BOLD:ACG0412	Beilschmiedia tovarensis	Lauraceae	8
Histura panamana	Tortricidae	Lepidoptera	BOLD:ABV2176	Beilschmiedia tovarensis	Lauraceae	23
Steblopotamia streblopa	Tortricidae	Lepidoptera	BOLD:ABV2183	Beilschmiedia tovarensis	Lauraceae	1
Oeco sp. lep4SG	Oecophoridae	Lepidoptera	BOLD:ABV2156	Beilschmiedia tovarensis	Lauraceae	2
Pagiocerus frontalis	Curculionidae	Coleoptera	BOLD:ABV0305	Beilschmiedia tovarensis	Lauraceae	2072
Pyra sp. lep248SG	Pyralidae	Lepidoptera		Beilschmiedia tovarensis	Lauraceae	2
Pyra sp. lep346SG	Pyralidae	Lepidoptera	BOLD:AAA1807	Beilschmiedia tovarensis	Lauraceae	3
Aeatus sp. cur108SG	Curculionidae	Coleoptera		Bignonia aequinoctialis	Bignoniaceae	1
Clydonopteron pomponius	Crambidae	Lepidoptera	BOLD:AAP2098	Bignonia aequinoctialis	Bignoniaceae	1
Curc sp. cur177SG	Curculionidae	Coleoptera	BOLD:ACJ5713	Bignonia aequinoctialis	Bignoniaceae	22
Curc sp. cur188SG	Curculionidae	Coleoptera	BOLD:ACJ5921	Bignonia aequinoctialis	Bignoniaceae	3
Semnorrhynchus fulvopictus	Curculionidae	Coleoptera	BOLD:ABV0293	Bignonia aequinoctialis	Bignoniaceae	1
Semnorrhynchus sp. cur44SG	Curculionidae	Coleoptera	BOLD:ABV0295	Bignonia aequinoctialis	Bignoniaceae	2
Oxytenopterus sp. cur204SG	Curculionidae	Coleoptera	BOLD:ACJ3891	Buchenavia tetraphylla	Combretaceae	152
Loncophorus sp. cur16SG	Curculionidae	Coleoptera	BOLD:ABV3554	Bunchosia nitida	Malpighiaceae	14
Aeatus sp. cur262SG	Curculionidae	Coleoptera	BOLD:ACJ5665	Callichlamys latifolia	Bignoniaceae	7
Clydonopteron pomponius	Crambidae	Lepidoptera	BOLD:AAP2098	Callichlamys latifolia	Bignoniaceae	29
Cryptorhynchus sp. cur54SG	Curculionidae	Coleoptera	BOLD:ACJ5914	Callichlamys latifolia	Bignoniaceae	1
Curc sp. cur184SG	Curculionidae	Coleoptera	BOLD:ACJ5817	Callichlamys latifolia	Bignoniaceae	1
Anchonus sp. cur32SG	Curculionidae	Coleoptera		Calophyllum longifolium	Clusiaceae	1

Insect species	Insect family	Insect order	BIN	Host species	Host family	Count
Conotrachelus sp. cur124SG nr. punctiventris	Curculionidae	Coleoptera	BOLD:ACG1073	Calophyllum longifolium	Clusiaceae	46
Conotrachelus sp. cur164SG	Curculionidae	Coleoptera	BOLD:ACG0847	Calophyllum longifolium	Clusiaceae	1
Conotrachelus sp. cur22SG nr. punctiventris	Curculionidae	Coleoptera		Calophyllum longifolium	Clusiaceae	1
Conotrachelus sp. cur30SG	Curculionidae	Coleoptera		Calophyllum longifolium	Clusiaceae	29
Curc sp. cur183SG	Curculionidae	Coleoptera	BOLD:ACJ5814	Calophyllum longifolium	Clusiaceae	5
Gele sp. lep106SG	Gelechiidae	Lepidoptera	BOLD:ABV2170	Calophyllum longifolium	Clusiaceae	8
Geobyrza nodifera	Curculionidae	Coleoptera		Calophyllum longifolium	Clusiaceae	1
Bruc sp. bru47SG	Chrysomelidae	Coleoptera	BOLD:ACJ4644	Canavalia dictyota	Fabaceae	2
Bruc sp. bru55SG	Chrysomelidae	Coleoptera		Canavalia dictyota	Fabaceae	4
Bruc sp. bru42SG	Chrysomelidae	Coleoptera	BOLD:ACJ4573	Canavalia gladiata	Fabaceae	6
Curc sp. cur213SG	Curculionidae	Coleoptera		Capparidastrum frondosa	Capparaceae	2
Pyra sp. lep291SG	Pyralidae	Lepidoptera	BOLD:ACG2223	Capparidastrum frondosa	Capparaceae	4
Pyra sp. lep307SG	Pyralidae	Lepidoptera		Capparidastrum frondosa	Capparaceae	1
Schacontia AAP1991	Pyralidae	Lepidoptera	BOLD:AAP1991	Capparidastrum frondosa	Capparaceae	11
Conotrachelus sp. cur132SG	Curculionidae	Coleoptera	BOLD:ACC9965	Casearia commersoniana	Salicaceae	9
Ricula sp. lep494SG	Tortricidae	Lepidoptera	BOLD:ACJ4292	Casearia commersoniana	Salicaceae	4
Anthonomus sp. cur45SG	Curculionidae	Coleoptera	BOLD:ABW5965	Casearia guianensis	Salicaceae	2
Conotrachelus sp. cur132SG	Curculionidae	Coleoptera	BOLD:ACC9965	Casearia guianensis	Salicaceae	29
Gele sp. lep308SG	Gelechiidae	Lepidoptera		Casearia guianensis	Salicaceae	1
Oeco sp. lep311SG	Oecophoridae	Lepidoptera	BOLD:ACJ4735	Cavanillesia platanifolia	Malvaceae	4
Pyra sp. lep312SG	Pyralidae	Lepidoptera		Cavanillesia platanifolia	Malvaceae	1
Conotrachelus sp. cur203SG	Curculionidae	Coleoptera	BOLD:ACJ3710	Cedrela odorata	Meliaceae	5
Cerambycidae sp. colcer3SG	Cerambycidae	Coleoptera	BOLD:ABV0428	Ceiba pentandra	Malvaceae	1
Cryptorhynchus sp. cur26SG	Curculionidae	Coleoptera	BOLD:ABU9975	Ceiba pentandra	Malvaceae	512
Lechriops parotica	Curculionidae	Coleoptera	BOLD:ABU9974	Ceiba pentandra	Malvaceae	17
Pagiocerus frontalis	Curculionidae	Coleoptera	BOLD:ABV0305	Ceiba pentandra	Malvaceae	1
Curc sp. cur208SG	Curculionidae	Coleoptera	BOLD:ACL6950	Celtis iguanaea	Cannabaceae	14

Insect species	Insect family	Insect order	BIN	Host species	Host family	Count
Cosm sp. lep406SG	Cosmopterigidae	Lepidoptera		Chamaedorea tepejilote	Arecaceae	7
Conotrachelus sp. cur181SG	Curculionidae	Coleoptera		Chrysophyllum argenteum	Sapotaceae	3
Gele sp. lep264SG	Gelechiidae	Lepidoptera	BOLD:ACG1531	Chrysophyllum argenteum	Sapotaceae	4
Myrmex sp. 128SG	Curculionidae	Coleoptera		Chrysophyllum argenteum	Sapotaceae	4
Myrmex sp. cur48SG nr. panamensis	Curculionidae	Coleoptera	BOLD:ACG0025	Chrysophyllum argenteum	Sapotaceae	2
Heilipus draco	Curculionidae	Coleoptera	BOLD:ABV0306	Cinnamomum triplinerve	Lauraceae	2
Riculorampha ancyloides	Tortricidae	Lepidoptera	BOLD:ABV2167	Cinnamomum triplinerve	Lauraceae	4
Oeco sp. lep13SG	Oecophoridae	Lepidoptera	BOLD:AAA0941	Cnestidium rufescens	Connaraceae	5
Conotrachelus sp. cur92SG	Curculionidae	Coleoptera	BOLD:ACD0125	Coccoloba excelsa	Polygonaceae	8
Curc sp. cur201SG	Curculionidae	Coleoptera	BOLD:ACL6812	Coccoloba excelsa	Polygonaceae	2
Curc sp. cur203SG	Curculionidae	Coleoptera	BOLD:ACL6814	Coccoloba excelsa	Polygonaceae	44
Curc sp. cur213SG	Curculionidae	Coleoptera		Coccoloba excelsa	Polygonaceae	8
Conotrachelus pumilio	Curculionidae	Coleoptera	BOLD:ACF9943	Coccoloba manzinellensis	Polygonaceae	33
Conotrachelus sp. cur92SG	Curculionidae	Coleoptera	BOLD:ACD0125	Coccoloba manzinellensis	Polygonaceae	105
Curc sp. cur193SG	Curculionidae	Coleoptera	BOLD:ACL5605	Coccoloba manzinellensis	Polygonaceae	2
Curc sp. cur198SG	Curculionidae	Coleoptera	BOLD:ACL6681	Coccoloba manzinellensis	Polygonaceae	3
Amblycerus perfectus	Chrysomelidae	Coleoptera	BOLD:ABV3602	Combretum fruticosum	Combretaceae	26
Amblycerus sp. bru17SG	Chrysomelidae	Coleoptera	BOLD:ABV3597	Combretum fruticosum	Combretaceae	3
Anchonus sp. cur62SG	Curculionidae	Coleoptera	BOLD:ABV0290	Combretum fruticosum	Combretaceae	1
Conotrachelus sp. cur141SG	Curculionidae	Coleoptera		Combretum fruticosum	Combretaceae	1
Curc sp. cur129SG	Curculionidae	Coleoptera		Combretum fruticosum	Combretaceae	1
Curc sp. cur187SG	Curculionidae	Coleoptera	BOLD:ACJ5920	Combretum fruticosum	Combretaceae	8
Rhyssomatus sp. cur72SG	Curculionidae	Coleoptera		Combretum fruticosum	Combretaceae	2
Amblycerus sp. bru17SG	Chrysomelidae	Coleoptera	BOLD:ABV3597	Combretum laxum	Combretaceae	3
Depr sp. lep186SG	Depressariidae	Lepidoptera	BOLD:ABV2153	Connarus panamensis	Connaraceae	5
Oeco sp. lep13SG	Oecophoridae	Lepidoptera	BOLD:AAA0941	Connarus panamensis	Connaraceae	6
Depr sp. lep186SG	Depressariidae	Lepidoptera	BOLD:ABV2153	Connarus turczaninowii	Connaraceae	1

Insect species	Insect family	Insect order	BIN	Host species	Host family	Count
Oeco sp. lep13SG	Oecophoridae	Lepidoptera	BOLD:AAA0941	Connarus turczaninowii	Connaraceae	4
Amblycerus vegai	Chrysomelidae	Coleoptera	BOLD:ACG1138	Cordia alliodora	Cordiaceae	27
Cosm sp. lep313SG	Cosmopterigidae	Lepidoptera	BOLD:ACU7675	Cordia alliodora	Cordiaceae	1
Aeatus vestitus	Curculionidae	Coleoptera		Cordia bicolor	Cordiaceae	94
Amblycerus sp. bru59SG	Chrysomelidae	Coleoptera	BOLD:ACJ4830	Cordia bicolor	Cordiaceae	4
Amblycerus championi	Chrysomelidae	Coleoptera		Cordia bicolor	Cordiaceae	1
Amblycerus sp. bru22SG	Chrysomelidae	Coleoptera	BOLD:ACJ4831	Cordia bicolor	Cordiaceae	92
Bruc sp. bru44SG	Chrysomelidae	Coleoptera	BOLD:ACJ4642	Cordia bicolor	Cordiaceae	4
Bruc sp. bru45SG	Chrysomelidae	Coleoptera	BOLD:ACJ4645	Cordia bicolor	Cordiaceae	2
Eurn sp. hym166SG	Eurytomidae	Hymenoptera		Cordia bicolor	Cordiaceae	2
Eurn sp. hym276SG	Eurytomidae	Hymenoptera		Cordia bicolor	Cordiaceae	1
Eury sp. hym167SG	Eurytomidae	Hymenoptera		Cordia lasiocalyx	Cordiaceae	1
Eurytoma sp. 1SG	Eurytomidae	Hymenoptera		Cordia lasiocalyx	Cordiaceae	1
Eurytoma sp. 2SG	Eurytomidae	Hymenoptera		Cordia lasiocalyx	Cordiaceae	1
Gele sp. lep514SG	Gelechiidae	Lepidoptera	BOLD:ACJ4456	Couratari guianensis	Lecythidaceae	2
Carmenta sp. lep1SG	Sesiidae	Lepidoptera	BOLD:ABV4212	Coussarea curvigemmia	Rubiaceae	45
Eury sp. hym268SG	Eurytomidae	Hymenoptera		Coussarea curvigemmia	Rubiaceae	1
Eury sp. hym29SG	Eurytomidae	Hymenoptera		Coussarea curvigemmia	Rubiaceae	2
Eury sp. hym30SG	Eurytomidae	Hymenoptera		Coussarea curvigemmia	Rubiaceae	4
Tyrannion sp. cur33SG	Curculionidae	Coleoptera	BOLD:ABV0980	Coussarea curvigemmia	Rubiaceae	141
Plocetes beluosus	Curculionidae	Coleoptera	BOLD:ACS6350	Coutarea hexandra	Rubiaceae	52
Oeco sp. lep13SG	Oecophoridae	Lepidoptera	BOLD:AAA0941	Cupania latifolia	Sapindaceae	1
Curc sp. cur173SG	Curculionidae	Coleoptera	BOLD:ACC9906	Cupania seemannii	Sapindaceae	52
Oeco sp. lep13SG	Oecophoridae	Lepidoptera	BOLD:AAA0941	Cupania seemannii	Sapindaceae	1
Pyra sp. lep310SG	Pyralidae	Lepidoptera	BOLD:AAG0530	Cupania seemannii	Sapindaceae	2
Strephonota tephraeus	Lycaenidae	Lepidoptera	BOLD:ABW7063	Cupania seemannii	Sapindaceae	1
Tmolus echionDHJ01	Lycaenidae	Lepidoptera	BOLD:AAK2129	Cupania seemannii	Sapindaceae	1

Insect species	Insect family	Insect order	BIN	Host species	Host family	Count
Baris sp. cur46SG	Curculionidae	Coleoptera	BOLD:ABV3556	Davilla nitida	Dilleniaceae	2
Oeco sp. lep192SG	Oecophoridae	Lepidoptera	BOLD:ACJ4736	Davilla nitida	Dilleniaceae	2
Baris sp. cur100SG	Curculionidae	Coleoptera		Desmoncus orthacanthos	Arecaceae	6
Cyrionyx sp. cur69SG	Curculionidae	Coleoptera		Desmopsis panamensis	Annonaceae	1
Cyrionyx sp. cur91SG	Curculionidae	Coleoptera	BOLD:ACC9960	Desmopsis panamensis	Annonaceae	93
Talponia sp. lep40SG	Tortricidae	Lepidoptera	BOLD:ABV2175	Desmopsis panamensis	Annonaceae	41
Caryedes brasiliensis	Chrysomelidae	Coleoptera		Dioclea wilsonii	Fabaceae	679
Baris sp. cur7SG	Curculionidae	Coleoptera	BOLD:ABV3553	Doliocarpus major	Dilleniaceae	31
Pseudobaris sp. cur19SG	Curculionidae	Coleoptera		Doliocarpus major	Dilleniaceae	1
Baris sp. cur7SG	Curculionidae	Coleoptera	BOLD:ABV3553	Doliocarpus multiflorus	Dilleniaceae	197
Borisauletes sp. cur1SG	Curculionidae	Coleoptera	BOLD:ABV3591	Doliocarpus multiflorus	Dilleniaceae	11
Pyra sp. lep117SG	Pyralidae	Lepidoptera	BOLD:AAA3587	Doliocarpus multiflorus	Dilleniaceae	1
Sycophila sp. 3SG	Eurytomidae	Hymenoptera		Doliocarpus multiflorus	Dilleniaceae	1
Baris sp. cur7SG	Curculionidae	Coleoptera	BOLD:ABV3553	Doliocarpus olivaceus	Dilleniaceae	44
Borisauletes sp. cur1SG	Curculionidae	Coleoptera	BOLD:ABV3591	Doliocarpus olivaceus	Dilleniaceae	3
Grapholitini sp. lep181SG	Tortricidae	Lepidoptera		Doliocarpus olivaceus	Dilleniaceae	5
Amblycerus sp. bru28SG	Chrysomelidae	Coleoptera		Drypetes standleyi	Putranjivaceae	10
Mimosestes enterolobii	Chrysomelidae	Coleoptera	BOLD:ABV3595	Enterolobium cyclocarpum	Fabaceae	20
Mimosestes enterolobii	Chrysomelidae	Coleoptera	BOLD:ABV3595	Enterolobium schomburgkii	Fabaceae	3
Gele sp. lep41SG	Gelechiidae	Lepidoptera	BOLD:ABV2154	Eugenia nesiotica	Myrtaceae	2
Atractomerus caligatus	Curculionidae	Coleoptera	BOLD:ABU8995	Eugenia oerstediana	Myrtaceae	24
Curc sp. cur178SG	Curculionidae	Coleoptera	BOLD:ACJ5755	Eugenia oerstediana	Myrtaceae	2
Curc sp. cur210SG	Curculionidae	Coleoptera	BOLD:ACL7823	Eugenia oerstediana	Myrtaceae	2
Gele sp. lep41SG	Gelechiidae	Lepidoptera	BOLD:ABV2154	Eugenia oerstediana	Myrtaceae	1
Conotrachelus sp. cur11SG	Curculionidae	Coleoptera	BOLD:ACJ5935	Eugenia venezuelensis	Myrtaceae	5
Carmenta sp. lep1SG	Sesiidae	Lepidoptera	BOLD:ABV4212	Faramea luteovirens	Rubiaceae	46
Carmenta sp. lep1SG	Sesiidae	Lepidoptera	BOLD:ABV4212	Faramea occidentalis	Rubiaceae	159

Insect species	Insect family	Insect order	BIN	Host species	Host family	Count
Carmenta sp. lep539SG	Sesiidae	Lepidoptera	BOLD:ABV4213	Faramea occidentalis	Rubiaceae	1
Eurn sp. hym199SG	Eurytomidae	Hymenoptera		Faramea occidentalis	Rubiaceae	1
Eurn sp. hym275SG	Eurytomidae	Hymenoptera		Faramea occidentalis	Rubiaceae	2
Eury sp. hym37SG	Eurytomidae	Hymenoptera		Faramea occidentalis	Rubiaceae	1
Gele sp. lep106SG	Gelechiidae	Lepidoptera		Faramea occidentalis	Rubiaceae	1
Sycophila sp. 3SG	Eurytomidae	Hymenoptera		Faramea occidentalis	Rubiaceae	23
Agao sp. hym89SG	Agaonidae	Hymenoptera		Ficus citrifolia	Moraceae	1
Ceratopus sp. cur259SG	Curculionidae	Coleoptera	BOLD:ACL6680	Ficus citrifolia	Moraceae	2
Ceratopus sp. cur81SG	Curculionidae	Coleoptera	BOLD:ACJ3977	Ficus citrifolia	Moraceae	4
Cosm sp. lep5SG	Cosmopterigidae	Lepidoptera	BOLD:ABV2189	Ficus citrifolia	Moraceae	7
Gele sp. lep282SG	Pyralidae	Lepidoptera		Ficus citrifolia	Moraceae	1
Pyra sp. lep147SG	Pyralidae	Lepidoptera	BOLD:ACJ4602	Ficus citrifolia	Moraceae	2
Pyra sp. lep147SG	Pyralidae	Lepidoptera	BOLD:ACJ4602	Ficus colubrinae	Moraceae	1
Agao sp. hym105SG	Agaonidae	Hymenoptera		Ficus insipida	Moraceae	11
Ceratopus bisignatus	Curculionidae	Coleoptera	BOLD:ABV0300	Ficus insipida	Moraceae	51
Ceratopus sp. cur2SG	Curculionidae	Coleoptera	BOLD:ABV0301	Ficus insipida	Moraceae	48
Ceratopus sp. cur5SG	Curculionidae	Coleoptera	BOLD:ACG0136	Ficus insipida	Moraceae	1
Ceratopus sp. cur5SG	Curculionidae	Coleoptera	BOLD:ACG0136	Ficus obtusifolia	Moraceae	2
Ceratopus sp. cur137SG	Curculionidae	Coleoptera	BOLD:ACG0631	Ficus popenoei	Moraceae	11
Ceratopus sp. cur5SG	Curculionidae	Coleoptera	BOLD:ACG0136	Ficus popenoei	Moraceae	28
Ceratopus sp. cur81SG	Curculionidae	Coleoptera	BOLD:ACJ3977	Ficus popenoei	Moraceae	5
Eury sp. hym70SG	Eurytomidae	Hymenoptera		Ficus popenoei	Moraceae	1
Curc sp. cur183SG	Curculionidae	Coleoptera	BOLD:ACJ5814	Ficus tonduzii	Moraceae	24
Ceratopus sp. cur237SG	Curculionidae	Coleoptera		Ficus trigonata	Moraceae	1
Ceratopus sp. cur238SG	Curculionidae	Coleoptera	BOLD:ACG0137	Ficus trigonata	Moraceae	1
Ceratopus sp. cur38SG	Curculionidae	Coleoptera	BOLD:ABV0298	Ficus trigonata	Moraceae	21
Ceratopus sp. cur5SG	Curculionidae	Coleoptera	BOLD:ACG0136	Ficus trigonata	Moraceae	4

Insect species	Insect family	Insect order	BIN	Host species	Host family	Count
Ceratopus sp. cur81SG	Curculionidae	Coleoptera	BOLD:ACJ3977	Ficus trigonata	Moraceae	291
Ceratopus bisignatus	Curculionidae	Coleoptera	BOLD:ABV0300	Ficus yoponensis	Moraceae	3
Clydonopteron pomponius	Crambidae	Lepidoptera	BOLD:AAP2098	Fridericia candicans	Bignoniaceae	2
Pseudobaris sp. cur188SG	Curculionidae	Coleoptera	BOLD:ACJ3578	Fridericia candicans	Bignoniaceae	3
Curc sp. cur181SG	Curculionidae	Coleoptera	BOLD:ACJ5812	Fridericia florida	Bignoniaceae	2
Curc sp. cur182SG	Curculionidae	Coleoptera	BOLD:ACJ5813	Fridericia florida	Bignoniaceae	2
Cosm sp. lep34SG	Cosmopterigidae	Lepidoptera	BOLD:ABV2184	Garcinia madruno	Clusiaceae	3
Cosm sp. lep34SG	Cosmopterigidae	Lepidoptera	BOLD:ABV2184	Garcinia recondita	Clusiaceae	254
Eriosocia guttifera	Tortricidae	Lepidoptera	BOLD:ABV2165	Garcinia recondita	Clusiaceae	3
Grapholita mabea	Tortricidae	Lepidoptera	BOLD:ABV2185	Garcinia recondita	Clusiaceae	5
Spinipogon triangularis	Tortricidae	Lepidoptera	BOLD:AAA2890	Guapira standleyana	Nyctaginaceae	7
Heli sp. lep102SG	Heliodinidae	Lepidoptera	BOLD:ABV2172	Guapira standleyana	Nyctaginaceae	3
Heli sp. lep52SG	Heliodinidae	Lepidoptera	BOLD:ABV2180	Guapira standleyana	Nyctaginaceae	50
Sternechus sp. cur65SG	Curculionidae	Coleoptera	BOLD:ABV0288	Guapira standleyana	Nyctaginaceae	190
Conotrachelus sp. cur138SG	Curculionidae	Coleoptera	BOLD:ACG1267	Guarea grandifolia	Meliaceae	14
Conotrachelus sp. cur182SG	Curculionidae	Coleoptera	BOLD:ACJ3628	Guarea grandifolia	Meliaceae	1
Conotrachelus verticalis	Curculionidae	Coleoptera	BOLD:ACJ6094	Guarea grandifolia	Meliaceae	1
Myrmex sp. cur48SG nr. panamensis	Curculionidae	Coleoptera	BOLD:ACG0025	Guarea grandifolia	Meliaceae	2
Semnorrhynchus fulvopictus	Curculionidae	Coleoptera	BOLD:ABV0293	Guarea grandifolia	Meliaceae	5
Sesi sp. lep101	Sesiidae	Lepidoptera	BOLD:ABV2173	Guarea grandifolia	Meliaceae	2
Conotrachelus sp. cur138SG	Curculionidae	Coleoptera	BOLD:ACG1267	Guarea guidonia	Meliaceae	20
Cosm sp. lep425SG	Cosmopterigidae	Lepidoptera	BOLD:ACJ4377	Guarea guidonia	Meliaceae	2
Anthonomus sp. cur150SG	Curculionidae	Coleoptera		Guatteria lucens	Annonaceae	1
Pseudomopsis sp. cur254SG	Curculionidae	Coleoptera	BOLD:ACJ5756	Guatteria lucens	Annonaceae	2
Pseudomopsis sp. cur34SG	Curculionidae	Coleoptera	BOLD:ABV0299	Guatteria lucens	Annonaceae	10
Amblycerus sp. bru39SG	Chrysomelidae	Coleoptera	BOLD:ACJ3956	Guazuma ulmifolia	Malvaceae	39
Amblycerus cistelinus	Chrysomelidae	Coleoptera	BOLD:ACG0463	Guazuma ulmifolia	Malvaceae	72

Insect species	Insect family	Insect order	BIN	Host species	Host family	Count
Amblycerus sp. bru30SG	Chrysomelidae	Coleoptera	BOLD:ABW8381	Guazuma ulmifolia	Malvaceae	15
Curc sp. cur202SG	Curculionidae	Coleoptera	BOLD:ACL6813	Guettarda foliacea	Rubiaceae	2
Busckiella lecythidis	Curculionidae	Coleoptera	BOLD:ACF9829	Gustavia superba	Lecythidaceae	8
Carmenta foraseminis	Sesiidae	Lepidoptera	BOLD:ABV2190	Gustavia superba	Lecythidaceae	83
Lamprospilus collucia	Lycaenidae	Lepidoptera	BOLD:AAE6101	Gustavia superba	Lecythidaceae	1
Ziegleria hesperitisDHJ01	Lycaenidae	Lepidoptera	BOLD:AAD4593	Gustavia superba	Lecythidaceae	2
Aeatus sp. cur144SG	Curculionidae	Coleoptera		Handroanthus guayacan	Bignoniaceae	34
Eury sp. hym128SG	Eurytomidae	Hymenoptera		Handroanthus guayacan	Bignoniaceae	3
Hypocosmia bimaculalis	Pyralidae	Lepidoptera	BOLD:AAA1759	Handroanthus guayacan	Bignoniaceae	2
Lamprospilus collucia	Lycaenidae	Lepidoptera	BOLD:AAE6101	Handroanthus guayacan	Bignoniaceae	1
Grapholita? sp. lep55SG	Tortricidae	Lepidoptera	BOLD:ACJ4358	Hasseltia floribunda	Salicaceae	1
Gele sp. lep275SG	Gelechiidae	Lepidoptera	BOLD:ABV2166	Heisteria acuminata	Erythropalaceae	1
Maemactes sp. cur75SG	Curculionidae	Coleoptera	BOLD:ABV0302	Heisteria acuminata	Erythropalaceae	1
Ricula croceus	Tortricidae	Lepidoptera	BOLD:ABV2186	Heisteria acuminata	Erythropalaceae	16
Eulechriops sp. cur53SG	Curculionidae	Coleoptera	BOLD:ABV0292	Heisteria concinna	Erythropalaceae	3
Platynota obliqua complex	Tortricidae	Lepidoptera		Heisteria concinna	Erythropalaceae	1
Ricula croceus	Tortricidae	Lepidoptera	BOLD:ABV2186	Heisteria concinna	Erythropalaceae	81
Strymon ziba	Lycaenidae	Lepidoptera	BOLD:AAJ4460	Heliconia platystachys	Heliconiaceae	2
Anthonominae sp. 248SG	Curculionidae	Coleoptera	BOLD:ACL7471	Heteropteris laurifolia	Malpighiaceae	51
Curc sp. cur194SG	Curculionidae	Coleoptera	BOLD:ACL5639	Heteropteris laurifolia	Malpighiaceae	2
Eurn sp. hym48SG	Eurytomidae	Hymenoptera		Hieronyma alchorneoides	Euphorbiaceae	1
Eurn sp. hym49SG	Eurytomidae	Hymenoptera		Hieronyma alchorneoides	Euphorbiaceae	7
Eury sp. hym91SG	Eurytomidae	Hymenoptera		Hieronyma alchorneoides	Euphorbiaceae	2
Eury sp. hym92SG	Eurytomidae	Hymenoptera		Hieronyma alchorneoides	Euphorbiaceae	3
Eury sp. hym93SG	Eurytomidae	Hymenoptera		Hieronyma alchorneoides	Euphorbiaceae	3
Sycophila sp. hym243SG	Eurytomidae	Hymenoptera		Hieronyma alchorneoides	Euphorbiaceae	2
Anthonominae sp. 247SG	Curculionidae	Coleoptera	BOLD:ACL6562	Hiraea reclinata	Malpighiaceae	6

Insect species	Insect family	Insect order	BIN	Host species	Host family	Count
Anthonomus sp. cur133SG	Curculionidae	Coleoptera	BOLD:ABV0303	Hiraea reclinata	Malpighiaceae	2
Bothryopteron darlingtoni	Brentidae	Coleoptera	BOLD:ACG1146	Hiraea reclinata	Malpighiaceae	78
Bothryopteron sp. cur85SG	Brentidae	Coleoptera	BOLD:ABU8658	Hiraea reclinata	Malpighiaceae	5
Bothryopteron sp. 23SG	Brentidae	Coleoptera	BOLD:ABU8657	Hiraea smilacina	Malpighiaceae	6
Cosm sp. lep499SG	Cosmopterigidae	Lepidoptera	BOLD:ACJ4545	Hirtella triandra	Chrysobalanaceae	2
Cosm sp. lep542SG	Cosmopterigidae	Lepidoptera	BOLD:ACJ4462	Hirtella triandra	Chrysobalanaceae	1
Eurn sp. hym97SG	Eurytomidae	Hymenoptera		Hirtella triandra	Chrysobalanaceae	5
Eurytoma? sp. hym95SG	Eurytomidae	Hymenoptera		Hirtella triandra	Chrysobalanaceae	10
Tenuipetiolus? sp. hym98SG	Eurytomidae	Hymenoptera		Hirtella triandra	Chrysobalanaceae	2
Myrmex sp. cur48SG nr. panamensis	Curculionidae	Coleoptera	BOLD:ACG0025	Hura crepitans	Euphorbiaceae	1
Pyra sp. lep296SG	Pyralidae	Lepidoptera	BOLD:AAH5480	Hymenaea courbaril	Fabaceae	230
Rhinochenus stigna	Curculionidae	Coleoptera	BOLD:ACG0000	Hymenaea courbaril	Fabaceae	152
Gele sp. lep295SG	Gelechiidae	Lepidoptera		Inga cocleensis	Fabaceae	1
Anth sp. ant3SG	Anthribidae	Coleoptera	BOLD:ACG0844	Inga cocleensis	Fabaceae	1
Conotrachelus sp. cur104SG	Curculionidae	Coleoptera		Inga laurina	Fabaceae	5
Pyra sp. lep9SG	Pyralidae	Lepidoptera	BOLD:ABV2171	Inga laurina	Fabaceae	4
Rhyssomatus sp. cur174SG	Curculionidae	Coleoptera	BOLD:ACJ4033	Inga laurina	Fabaceae	41
Cosm sp. lep274SG	Cosmopterigidae	Lepidoptera	BOLD:AAL8816	Inga mucuna	Fabaceae	7
Oeco sp. lep289SG	Oecophoridae	Lepidoptera		Inga mucuna	Fabaceae	1
Oeco sp. lep293SG	Oecophoridae	Lepidoptera	BOLD:AAA1020	Inga mucuna	Fabaceae	1
Cydia pyraspis complex ACJ4819	Tortricidae	Lepidoptera	BOLD:ACJ4819	Inga multijuga	Fabaceae	1
Synanthedon sp. lep500SG	Sesiidae	Lepidoptera	BOLD:ACJ4196	Inga multijuga	Fabaceae	1
Atryparius sp. 1SG	Cerambycidae	Coleoptera	BOLD:ABV0425	Inga punctata	Fabaceae	2
Cosm sp. lep274SG	Cosmopterigidae	Lepidoptera	BOLD:AAL8816	Inga punctata	Fabaceae	1
Cosm sp. lep274SG	Cosmopterigidae	Lepidoptera	BOLD:AAL8816	Inga ruiziana	Fabaceae	2
Cosm sp. lep425SG	Cosmopterigidae	Lepidoptera	BOLD:ACJ4377	Inga ruiziana	Fabaceae	1
Gele sp. lep281SG	Gelechiidae	Lepidoptera	BOLD:ACJ4591	Inga ruiziana	Fabaceae	1

Insect species	Insect family	Insect order	BIN	Host species	Host family	Count
Gele sp. lep295SG	Gelechiidae	Lepidoptera		Inga sapindoides	Fabaceae	1
Synanthedon sp. lep413SG	Sesiidae	Lepidoptera	BOLD:ACJ4195	Inga sapindoides	Fabaceae	1
Gele sp. lep281SG	Gelechiidae	Lepidoptera	BOLD:ACJ4591	Inga thibaudiana	Fabaceae	1
Cydia pyraspis complex AAA4047	Tortricidae	Lepidoptera	BOLD:AAA4047	Inga umbellifera	Fabaceae	1
Curc sp. cur185SG	Curculionidae	Coleoptera	BOLD:ACJ5840	Jacaranda copaia	Bignoniaceae	30
Curc sp. cur186SG	Curculionidae	Coleoptera	BOLD:ACJ5919	Lacistema aggregatum	Lacistemataceae	66
Ricula lacistema	Tortricidae	Lepidoptera	BOLD:AAY4670	Lacistema aggregatum	Lacistemataceae	19
Ricula sp. 545SG nr. deflexa	Tortricidae	Lepidoptera	BOLD:ACU7912	Lacistema aggregatum	Lacistemataceae	1
Pycnotheantis sp. cur47SG	Curculionidae	Coleoptera	BOLD:ABV3492	Lacmellea panamensis	Apocynaceae	1
Anthonomus sp. cur45SG	Curculionidae	Coleoptera	BOLD:ABW5965	Laetia procera	Salicaceae	1
Carmenta foraseminis	Sesiidae	Lepidoptera	BOLD:ABV2190	Lafoensia punicifolia	Lythraceae	2
Gele sp. lep60SG	Gelechiidae	Lepidoptera	BOLD:ABV2178	Lafoensia punicifolia	Lythraceae	20
Conotrachelus posticatus	Curculionidae	Coleoptera	BOLD:ACJ3962	Licania platypus	Chrysobalanaceae	1
Conotrachelus sp. cur176SG	Curculionidae	Coleoptera	BOLD:ACC9932	Licania platypus	Chrysobalanaceae	38
Oeco sp. lep419SG	Oecophoridae	Lepidoptera		Lindackeria laurina	Achariaceae	1
Conotrachelus sp. cur152SG	Curculionidae	Coleoptera		Lonchocarpus heptaphyllus	Fabaceae	57
Pyra sp. lep449SG	Pyralidae	Lepidoptera	BOLD:AAA5505	Lonchocarpus heptaphyllus	Fabaceae	1
Coelocephalapion sp. cur13SG	Brentidae	Coleoptera	BOLD:ABV4175	Lonchocarpus luteomaculatus	Fabaceae	1
Ctenocolum colburni	Chrysomelidae	Coleoptera	BOLD:ABV3608	Lonchocarpus luteomaculatus	Fabaceae	2
Amblycerus simulator	Chrysomelidae	Coleoptera	BOLD:ABV3596	Luehea seemannii	Malvaceae	32
Cerambycidae sp. colcer5SG	Cerambycidae	Coleoptera	BOLD:ABW7350	Mabea occidentalis	Euphorbiaceae	1
Grapholita mabea	Tortricidae	Lepidoptera	BOLD:ABV2185	Mabea occidentalis	Euphorbiaceae	109
Apion sp. cur183SG	Brentidae	Coleoptera	BOLD:ACJ3666	Machaerium arboreum	Fabaceae	69
Eury sp. hym234SG	Eurytomidae	Hymenoptera		Machaerium arboreum	Fabaceae	25
Pyra sp. lep449SG	Pyralidae	Lepidoptera	BOLD:AAA5505	Machaerium milleflorum	Fabaceae	1
Apion sp. cur180SG	Brentidae	Coleoptera	BOLD:ACJ3612	Machaerium seemannii	Fabaceae	2
Talponia sp. lep273SG	Tortricidae	Lepidoptera	BOLD:ACG2213	Macrocnemum roseum	Rubiaceae	11

Insect species	Insect family	Insect order	BIN	Host species	Host family	Count
Megacerus lunulatus	Chrysomelidae	Coleoptera	BOLD:ABV3601	Maripa panamensis	Convolvulaceae	149
Oeco sp. lep4SG	Oecophoridae	Lepidoptera	BOLD:ABV2156	Maripa panamensis	Convolvulaceae	3
Spilomelinae sp. 80YB	Crambidae	Lepidoptera	BOLD:AAP1702	Maripa panamensis	Convolvulaceae	5
Bothryopteron darlingtoni	Brentidae	Coleoptera	BOLD:ACG1146	Mascagnia divaricata	Malpighiaceae	1
Amblycerus sp. bru27SG nr piurae	Chrysomelidae	Coleoptera		Mendoncia gracilis	Acanthaceae	4
Gele sp. lep185SG	Gelechiidae	Lepidoptera	BOLD:ABV2157	Miconia argentea	Melastomataceae	1
Sibinia sp. cur154SG	Curculionidae	Coleoptera	BOLD:ACJ5979	Mimosa pigra	Fabaceae	29
Gele sp. lep39SG	Gelechiidae	Lepidoptera	BOLD:ACG1530	Mouriri myrtilloides	Melastomataceae	4
Anthonomus sp. cur158SG	Curculionidae	Coleoptera	BOLD:ACF9809	Myrcia splendens_tip._gatunensis	Myrtaceae	4
Anthonomus sp. cur245SG	Curculionidae	Coleoptera	BOLD:ACS3899	Myrcia splendens_tip._gatunensis	Myrtaceae	3
Curc sp. cur179SG	Curculionidae	Coleoptera	BOLD:ACJ5779	Nectandra cissiflora	Lauraceae	4
Curc sp. cur204SG	Curculionidae	Coleoptera	BOLD:ACL6815	Nectandra cissiflora	Lauraceae	7
Heilipus draco	Curculionidae	Coleoptera	BOLD:ABV0306	Nectandra cissiflora	Lauraceae	4
Riculorampha ancyloides	Tortricidae	Lepidoptera	BOLD:ABV2167	Nectandra cissiflora	Lauraceae	16
Curc sp. cur175SG	Curculionidae	Coleoptera	BOLD:ACC9991	Nectandra lineata	Lauraceae	2
Curc sp. cur179SG	Curculionidae	Coleoptera	BOLD:ACJ5779	Nectandra lineata	Lauraceae	4
Pagiocerus frontalis	Curculionidae	Coleoptera	BOLD:ABV0305	Nectandra lineata	Lauraceae	15
Pyra sp. lep305SG	Pyralidae	Lepidoptera		Nectandra lineata	Lauraceae	2
Riculorampha ancyloides	Tortricidae	Lepidoptera	BOLD:ABV2167	Nectandra lineata	Lauraceae	6
Heli sp. lep438SG	Heliodinidae	Lepidoptera	BOLD:ACJ4711	Neea amplifolia	Nyctaginaceae	2
Pagiocerus frontalis	Curculionidae	Coleoptera	BOLD:ABV0305	Ocotea oblonga	Lauraceae	366
Riculorampha ancyloides	Tortricidae	Lepidoptera	BOLD:ABV2167	Ocotea oblonga	Lauraceae	7
Curc sp. cur176SG	Curculionidae	Coleoptera	BOLD:ACC9992	Ocotea puberula	Lauraceae	6
Pagiocerus frontalis	Curculionidae	Coleoptera	BOLD:ABV0305	Ocotea puberula	Lauraceae	71
Heilipus sp. cur15SG nr. lauri	Curculionidae	Coleoptera	BOLD:ABV0055	Ocotea whitei	Lauraceae	1
Oeco sp. lep4SG	Oecophoridae	Lepidoptera	BOLD:ABV2156	Ocotea whitei	Lauraceae	2
Riculorampha ancyloides	Tortricidae	Lepidoptera	BOLD:ABV2167	Ocotea whitei	Lauraceae	2

Insect species	Insect family	Insect order	BIN	Host species	Host family	Count
Curc sp. cur172SG	Curculionidae	Coleoptera		Oenocarpus mapora	Arecaceae	6
Curc sp. cur192SG	Curculionidae	Coleoptera	BOLD:ACJ6049	Oenocarpus mapora	Arecaceae	2
Geraeus sp. cur51SG	Curculionidae	Coleoptera	BOLD:ABV4317	Oenocarpus mapora	Arecaceae	112
Pycnotheantis sp. cur47SG	Curculionidae	Coleoptera	BOLD:ABV3492	Oenocarpus mapora	Arecaceae	98
Zyzzyva sp. cur42SG	Curculionidae	Coleoptera	BOLD:ABV3555	Oenocarpus mapora	Arecaceae	219
Curc sp. cur191SG	Curculionidae	Coleoptera	BOLD:ACJ6005	Ormosia macrocalyx	Fabaceae	2
Eulechriops sp. cur53SG	Curculionidae	Coleoptera	BOLD:ABV0292	Ouratea lucens	Ochnaceae	66
Heli sp. lep253SG	Heliodinidae	Lepidoptera	BOLD:ACJ1788	Ouratea lucens	Ochnaceae	30
Cosm sp. lep406SG	Cosmopterigidae	Lepidoptera	BOLD:ACJ4291	Paullinia fibrigera	Sapindaceae	1
Oeco sp. lep13SG	Oecophoridae	Lepidoptera	BOLD:AAA0941	Paullinia pinnata	Sapindaceae	5
Oeco sp. lep464SG	Oecophoridae	Lepidoptera		Paullinia pinnata	Sapindaceae	2
Sycophila sp. hym173SG	Eurytomidae	Hymenoptera		Paullinia pterocarpa	Sapindaceae	2
Oeco sp. lep466SG	Oecophoridae	Lepidoptera		Paullinia rugosa	Sapindaceae	1
Eurn sp. hym142SG	Eurytomidae	Hymenoptera		Paullinia turbacensis	Sapindaceae	28
Strephonota ambrax	Lycaenidae	Lepidoptera	BOLD:ABV4118	Paullinia turbacensis	Sapindaceae	1
Anthonomus sp. cur211SG nr. monostigma	Curculionidae	Coleoptera	BOLD:ACS4123	Pera arborea	Peraceae	4
Curc sp. cur211SG	Curculionidae	Coleoptera		Petrea volubilis	Verbenaceae	2
Conotrachelus sp. cur152SG	Curculionidae	Coleoptera	BOLD:ACC9959	Picramnia latifolia	Picramniaceae	167
Dichrorampha? sp. lep324SG	Tortricidae	Lepidoptera	BOLD:ACG1391	Picramnia latifolia	Picramniaceae	6
Pseudobaris sp. cur202SG	Curculionidae	Coleoptera	BOLD:ACJ3579	Platymiscium pinnatum	Fabaceae	1
Amblycerus sp. bru21SG	Chrysomelidae	Coleoptera	BOLD:ACJ4774	Platypodium elegans	Fabaceae	10
Atryparius sp. 1SG	Cerambycidae	Coleoptera	BOLD:ABV0425	Platypodium elegans	Fabaceae	14
Cydia? sp. lep356SG	Tortricidae	Lepidoptera	BOLD:ABV2351	Platypodium elegans	Fabaceae	1
Pyra sp. lep449SG	Pyralidae	Lepidoptera	BOLD:AAA5505	Platypodium elegans	Fabaceae	3
Cyrionyx sp. cur91SG	Curculionidae	Coleoptera		Pombalia prunifolia	Violaceae	11
Anchonus sp. cur53SG	Curculionidae	Coleoptera	BOLD:ACS6334	Poulsenia armata	Moraceae	1
Sycophila sp. 1SG	Eurytomidae	Hymenoptera		Poulsenia armata	Moraceae	12

Insect species	Insect family	Insect order	BIN	Host species	Host family	Count
Sycophila sp. 2SG	Eurytomidae	Hymenoptera		Poulsenia armata	Moraceae	13
Eubulus sp. cur151SG	Curculionidae	Coleoptera	BOLD:ACF9999	Pourouma bicolor	Urticaceae	4
Atryparius sp. 1SG	Cerambycidae	Coleoptera	BOLD:ABV0425	Pouteria reticulata	Sapotaceae	1
Conotrachelus sp. cur163SG	Curculionidae	Coleoptera		Pouteria reticulata	Sapotaceae	59
Conotrachelus turbatus	Curculionidae	Coleoptera	BOLD:ACJ3631	Pouteria reticulata	Sapotaceae	1
Curc sp. cur103SG	Curculionidae	Coleoptera		Pouteria reticulata	Sapotaceae	1
Curc sp. cur190SG	Curculionidae	Coleoptera	BOLD:ACJ5957	Pouteria reticulata	Sapotaceae	6
Curc sp. cur206SG	Curculionidae	Coleoptera	BOLD:ACL6820	Pouteria reticulata	Sapotaceae	2
Eubulus sp. cur90SG cf. dumicolus	Curculionidae	Coleoptera	BOLD:ACG1557	Pouteria reticulata	Sapotaceae	5
Myrmex sp. cur94SG	Curculionidae	Coleoptera	BOLD:ACG1997	Pouteria reticulata	Sapotaceae	2
Conotrachelus sp. cur17SG	Curculionidae	Coleoptera		Pouteria stipitata	Sapotaceae	3
Conotrachelus sp. cur18SG	Curculionidae	Coleoptera	BOLD:ACG2729	Pouteria stipitata	Sapotaceae	56
Carmenta sp. lep111SG	Sesiidae	Lepidoptera	BOLD:ABV4214	Prioria copaifera	Fabaceae	33
Cryptaspasma perseana	Tortricidae	Lepidoptera	BOLD:AAB0277	Prioria copaifera	Fabaceae	5
Eubulus fulvosquamis	Curculionidae	Coleoptera	BOLD:ABV0277	Prioria copaifera	Fabaceae	632
Anth sp. ant1SG	Anthribidae	Coleoptera	BOLD:ABV3606	Prioria copaifera	Fabaceae	9
Pyra sp. lep9SG	Pyralidae	Lepidoptera	BOLD:ABV2171	Prioria copaifera	Fabaceae	45
Apogeshna stenialis	Crambidae	Lepidoptera	BOLD:AAM8762	Protium tenuifolium	Burseraceae	4
Curc sp. cur174SG	Curculionidae	Coleoptera	BOLD:ACC9931	Protium tenuifolium	Burseraceae	2
Depr sp. lep8SG	Depressariidae	Lepidoptera	BOLD:ABV2188	Protium tenuifolium	Burseraceae	2
Lechriops sp. cur97SG	Curculionidae	Coleoptera		Protium tenuifolium	Burseraceae	2
Megacerus lunulatus	Chrysomelidae	Coleoptera	BOLD:ABV3601	Protium tenuifolium	Burseraceae	14
Cerambycidae sp. colcer3SG	Cerambycidae	Coleoptera	BOLD:ABV0428	Pseudobombax septenatum	Malvaceae	2
Colobothea sp. 1SG	Cerambycidae	Coleoptera	BOLD:ACJ3953	Pseudobombax septenatum	Malvaceae	1
Cryptorhynchus sp. cur26SG	Curculionidae	Coleoptera	BOLD:ABU9975	Pseudobombax septenatum	Malvaceae	5
Lechriops parotica	Curculionidae	Coleoptera	BOLD:ABU9974	Pseudobombax septenatum	Malvaceae	49
Platynota obliqua complex	Tortricidae	Lepidoptera	BOLD:ABV2162	Pseudobombax septenatum	Malvaceae	1

Insect species	Insect family	Insect order	BIN	Host species	Host family	Count
Cosm sp. lep528SG	Cosmopterigidae	Lepidoptera		Psychotria capitata	Rubiaceae	1
Eurn sp. hym253SG	Eurytomidae	Hymenoptera		Psychotria capitata	Rubiaceae	32
Sycophila sp. 2SG	Eurytomidae	Hymenoptera		Psychotria capitata	Rubiaceae	13
Eurn sp. hym137SG	Eurytomidae	Hymenoptera		Psychotria limonensis	Rubiaceae	5
Sycophila sp. 1SG	Eurytomidae	Hymenoptera		Psychotria limonensis	Rubiaceae	1
Sycophila sp. hym171SG	Eurytomidae	Hymenoptera		Psychotria marginata	Rubiaceae	3
Amblycerus pterocarpae	Chrysomelidae	Coleoptera		Pterocarpus hayesii	Fabaceae	2
Chelotonyx sp. 1SG	Curculionidae	Coleoptera	BOLD:ABV0294	Quararibea asterolepis	Malvaceae	93
Conotrachelus sp. cur141SG		Coleoptera	BOLD:ACC9894	Quararibea asterolepis	Malvaceae	24
Gele sp. lep19SG	Gelechiidae	Lepidoptera	BOLD:AAA0886	Quararibea asterolepis	Malvaceae	7
Myrmex sp. cur48SG nr. panamensis	Curculionidae	Coleoptera	BOLD:ACG0025	Quararibea asterolepis	Malvaceae	1
Cosm sp. lep38SG	Cosmopterigidae	Lepidoptera	BOLD:ABV2182	Rinorea sylvatica	Violaceae	47
Bruc sp. bru41SG	Chrysomelidae	Coleoptera	BOLD:ACJ4488	Senna reticulata	Fabaceae	108
Sennius lawrencei	Chrysomelidae	Coleoptera	BOLD:ACG0207	Senna reticulata	Fabaceae	192
Bruc sp. bru37SG	Chrysomelidae	Coleoptera	BOLD:ACJ4572	Senna undulata	Fabaceae	149
Fundella argentina	Pyralidae	Lepidoptera	BOLD:AAA0902	Senna undulata	Fabaceae	11
Pyra sp. 496SG	Pyralidae	Lepidoptera	BOLD:ACL6679	Serjania decapleuria	Sapindaceae	4
Geraeus sp. cur64SG	Curculionidae	Coleoptera	BOLD:ABV0289	Simarouba amara	Simaroubaceae	1
Conotrachelus sp. cur29SG	Curculionidae	Coleoptera		Sloanea terniflora	Elaeocarpaceae	1
Curc sp. cur205SG	Curculionidae	Coleoptera	BOLD:ACL6816	Socratea exorrhiza	Arecaceae	4
Eury sp. hym207SG	Eurytomidae	Hymenoptera		Socratea exorrhiza	Arecaceae	1
Eutoxus sp. cur21SG	Curculionidae	Coleoptera	BOLD:ACS9632	Socratea exorrhiza	Arecaceae	35
Gele sp. lep427SG	Gelechiidae	Lepidoptera	BOLD:ACJ4667	Souroubea sympetala	Marcgraviaceae	1
Pagiocerus frontalis	Curculionidae	Coleoptera	BOLD:ABV0305	Souroubea sympetala	Marcgraviaceae	1
Conotrachelus sp. cur8SG	Curculionidae	Coleoptera	BOLD:ABW8996	Spondias mombin	Anacardiaceae	78
Curc sp. cur189SG	Curculionidae	Coleoptera	BOLD:ACJ5951	Spondias mombin	Anacardiaceae	2
Ceratopus sp. cur2SG	Curculionidae	Coleoptera	BOLD:ABV0301	Spondias radlkoferi	Anacardiaceae	1

Insect species	Insect family	Insect order	BIN	Host species	Host family	Count
Conotrachelus sp. cur256SG	Curculionidae	Coleoptera	BOLD:ACD0167	Spondias radlkoferi	Anacardiaceae	2
Conotrachelus sp. cur8SG	Curculionidae	Coleoptera	BOLD:ABW8996	Spondias radlkoferi	Anacardiaceae	326
Cerambycidae sp. colcer1SG	Cerambycidae	Coleoptera	BOLD:ACJ3675	Sterculia apetala	Malvaceae	16
Cerambycidae sp. colcer3SG	Cerambycidae	Coleoptera	BOLD:ABV0428	Sterculia apetala	Malvaceae	2
Lepturges sp. 1SG	Cerambycidae	Coleoptera	BOLD:ABV0431	Sterculia apetala	Malvaceae	2
Eury sp. hym252SG	Eurytomidae	Hymenoptera		Stigmaphyllon lindenianum	Malpighiaceae	1
Carmenta sp. lep131	Sesiidae	Lepidoptera	BOLD:ABV2191	Strychnos panamensis	Loganiaceae	2
Curc sp. cur20SG	Curculionidae	Coleoptera		Strychnos panamensis	Loganiaceae	1
Eriosocia guttifera	Tortricidae	Lepidoptera	BOLD:ABV2165	Symphonia globulifera	Clusiaceae	97
Aeatus costulatus	Curculionidae	Coleoptera	BOLD:ABV0286	Tabebuia rosea	Bignoniaceae	21
Pagiocerus frontalis	Curculionidae	Coleoptera	BOLD:ABV0305	Tabebuia rosea	Bignoniaceae	2
Nealcidion sp. 1SG	Cerambycidae	Coleoptera	BOLD:ACJ3565	Tabernaemontana arborea	Apocynaceae	3
Amblycerus tachigaliae	Chrysomelidae	Coleoptera	BOLD:ABV3605	Tachigali panamensis	Fabaceae	16
Sennius sp. bru58SG	Chrysomelidae	Coleoptera	BOLD:ABV3603	Tachigali panamensis	Fabaceae	2
Sennius sp. bru8SG	Chrysomelidae	Coleoptera	BOLD:ABV3604	Tachigali panamensis	Fabaceae	2
Curc sp. cur131SG	Curculionidae	Coleoptera		Terminalia amazonia	Combretaceae	2
Curc sp. cur209SG	Curculionidae	Coleoptera	BOLD:ACL7472	Terminalia oblonga	Combretaceae	2
Baris sp. cur121SG	Curculionidae	Coleoptera	BOLD:ACG1145	Tetracera portobellensis	Dilleniaceae	12
Conotrachelus sp. cur8SG	Curculionidae	Coleoptera		Tetragastris panamensis	Burseraceae	1
Gele sp. lep3SG	Gelechiidae	Lepidoptera	BOLD:ABV2181	Tetragastris panamensis	Burseraceae	51
Anthonomus sp. cur136SG	Curculionidae	Coleoptera	BOLD:ACF9998	Tetrapterys discolor	Malpighiaceae	14
Phymatophosus sp. cur37SG	Curculionidae	Coleoptera	BOLD:ABU9761	Tetrapterys discolor	Malpighiaceae	44
Pseudomopsis sp. cur168SG	Curculionidae	Coleoptera	BOLD:ACJ5601	Tetrapterys discolor	Malpighiaceae	2
Curc sp. cur199SG	Curculionidae	Coleoptera	BOLD:ACL6683	Tetrapterys goudotiana	Malpighiaceae	2
Phymatophosus sp. cur107SG	Curculionidae	Coleoptera	BOLD:ACG1442	Tetrapterys goudotiana	Malpighiaceae	14
Phymatophosus sp. cur197SG	Curculionidae	Coleoptera		Tetrapterys goudotiana	Malpighiaceae	5
Cosm sp. lep5SG	Cosmopterigidae	Lepidoptera	BOLD:ABV2189	Tetrathylacium johansenii	Salicaceae	8

Insect species	Insect family	Insect order	BIN	Host species	Host family	Count
Atryparius sp. 1SG	Cerambycidae	Coleoptera	BOLD:ABV0425	Trattinnickia aspera	Burseraceae	1
Conotrachelus sp. cur139SG	Curculionidae	Coleoptera		Trichilia hirta	Meliaceae	2
Conotrachelus sp. cur10SG	Curculionidae	Coleoptera	BOLD:ACJ5904	Trichilia tuberculata	Meliaceae	78
Conotrachelus sp. cur175SG	Curculionidae	Coleoptera	BOLD:ACC9961	Trichilia tuberculata	Meliaceae	21
Pyra sp. lep231SG	Pyralidae	Lepidoptera	BOLD:ACF3503	Trichilia tuberculata	Meliaceae	2
Ricula sp. lep218SG	Tortricidae	Lepidoptera	BOLD:ACG2639	Trichilia tuberculata	Meliaceae	1
Oeco sp. lep441SG	Oecophoridae	Lepidoptera	BOLD:AAH5205	Trichospermum galeottii	Malvaceae	1
Conotrachelus sp. cur58SG	Curculionidae	Coleoptera	BOLD:ABV0304	Triplaris cumingiana	Polygonaceae	33
Oeco sp. lep250SG	Oecophoridae	Lepidoptera		Unonopsis pittieri	Annonaceae	1
Oxytenopterus sp. cur55SG	Curculionidae	Coleoptera	BOLD:ABV0291	Unonopsis pittieri	Annonaceae	21
Apion sp. cur255SG	Brentidae	Coleoptera		Vatairea erythrocarpa	Fabaceae	27
Curc sp. cur196SG	Curculionidae	Coleoptera	BOLD:ACL6563	Virola nobilis	Myristicaceae	2
Eubulus sp. cur24SG	Curculionidae	Coleoptera	BOLD:ABV4089	Virola nobilis	Myristicaceae	5
Lechriops sp. cur40SG	Curculionidae	Coleoptera	BOLD:ABV0297	Virola nobilis	Myristicaceae	12
Anthonomus sp. cur14SG	Curculionidae	Coleoptera	BOLD:ABV0278	Virola sebifera	Myristicaceae	1
Eubulus sp. cur24SG	Curculionidae	Coleoptera	BOLD:ABV4089	Virola sebifera	Myristicaceae	4
Anth sp. ant2SG	Anthribidae	Coleoptera	BOLD:ABW8380	Virola sebifera	Myristicaceae	1
Tallula sp. lep229SG	Pyralidae	Lepidoptera	BOLD:AAA3603	Zanthoxylum acuminatum	Rutaceae	1
Anthonomus sp. cur45SG	Curculionidae	Coleoptera	BOLD:ABW5965	Zuelania guidonia	Salicaceae	47
Platynota subargentea	Tortricidae	Lepidoptera	BOLD:AAA0948	Zuelania guidonia	Salicaceae	1

7 Table S3: Phylogenetic signal in seed predator incidence, seed predator richness, and seed predation rate

Table S3. Results from analyses testing for phylogenetic signal in the incidence of seed predators, seed predator richness, and seed predation rates in the sampled plant community. In the analyses of phylogenetic signal in incidence, *D* values significantly smaller than 1 indicate phylogenetic clustering. *D* values not significantly different from 0 suggest that the trait is as clumped as if it had evolved under Brownian evolution. In the analyses of phylogenetic signal in seed predator richness and rate, absolute values of κ smaller than 1 indicate that the phylogenetic signal is smaller than expected under a Brownian motion process. P-values <0.05 suggest that the phylogenetic signal is still larger than random.

Incidence	Seed predator richness	Seed predation rate					
Trait	Estimated D	PD=0	PD<1	K	P	K	P
Overall (all orders)	0.779	0.001	0.006	0.004	0.051	0.0006	0.082
Coleoptera	0.501	<0.001	<0.001				
Lepidoptera	0.662	0.005	0.001				
Hymenoptera	0.653	0.076	0.054				

8 Table S4: Analyses on host specialisation using a data set that excludes singleton observations

Table S4. The measured level of host specialisation will to some degree be influenced by the sampling intensity and/or potential errors in data recording leading to singleton interactions being recorded at a higher frequency than at which they occur in reality. To assess the robustness of the results presented in the main text, we re-run all analyses relating to host specialisation on three smaller data sets: 1) **well-sampled:** includes only 'well-sampled' insect species (species with a minimum of 10 individuals), no restrictions in terms of singleton interactions, 2) **singleton interactions excluded:** singleton interactions removed, but otherwise no restrictions in terms of number of insect individuals reared per species, and 3) **well-sampled, singleton interactions excluded:** singleton interactions removed, only well-sampled insect species included. In the summary tables below, these are all compared to the analyses presented in the main text ('**all data**'; including singleton interactions and no restrictions on the number of individuals per species).

Metric	All data	Well-sampled	Singleton interactions excluded	Well-sampled, singleton interactions excluded
d' (median)	0.831	0.990	0.897	1
d' (min)	0.204	0.447	0.204	0.511
d' (max)	1	1	1	1
H2'	0.972	0.983	0.975	0.986
% of non-specialists feeding on species in same genus	37.7%		43.1%	
% of non-specialists feeding on species in same family	62.2%		70.4%	

9 Figure S1: Effect of sample size on seed predator incidence

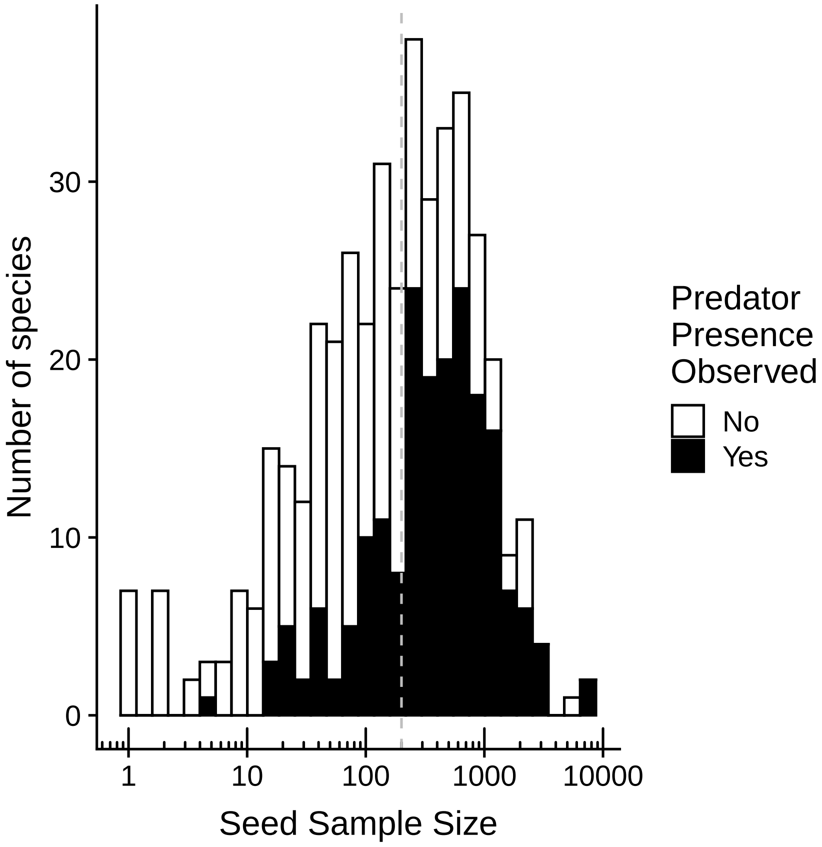

Figure S1 Histogram showing the frequency distribution of sampling effort across the 478 collected plant species. The black portion of the bars represent species from which seed predators were reared. For species with few collected seeds, the likelihood of encountering seed predators was smaller than for species with larger sample sizes (logistic regression: β=0.0001, SE=0.0003, z=5.396, P¡0.001). For species with sample sizes above 200 seeds (dashed line), the relationship between seed sample size and seed predator incidence was no longer statistically significant (logistic regression: β=0.0002, SE=0.0002, z=1.112, P=0.266).

10 Figure S2: Plant phylogeny including all plant species

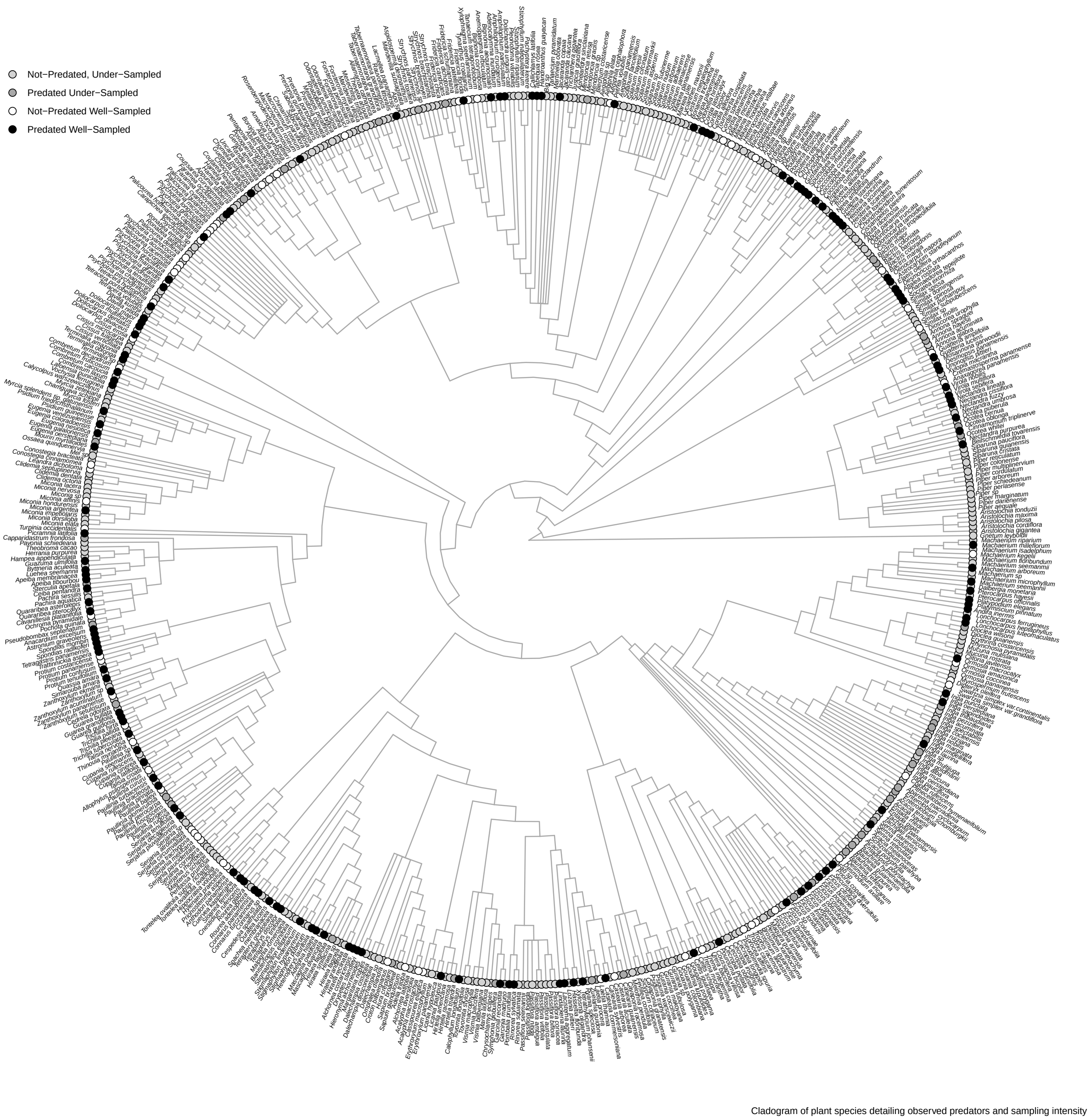

Figure S2. Presence or absence of seed predators (all orders combined) plotted against a plant phylogeny that includes all plant species sampled in this study for which phylogenetic information was available. The presence/absence of seed predators on well-samples plant species (minimum sample size of 200 seeds/fruits) is shown as black and white circles, respectively. For species with smaller sample sizes (¡200 seeds/fruits) presence/absence of seed predators is shown as dark versus light grey circles. Of the 478 plant species for which seed samples were collected for insect rearing, 58 (12.1%) could not be included in this figure because of lack of phylogenetic information.

11 Figure S3: Spine plots depicting relationships between seed predator incidence and plant traits

Figure S3. Spine plots depicting the relationships between seed predator incidence and the studied plant traits. The dark portion of the bars show the proportion of species within each trait category found to be attacked by one or more species of internally feeding insect seed predators. The width of each bar is proportionate to the number of plant species in the trait category.

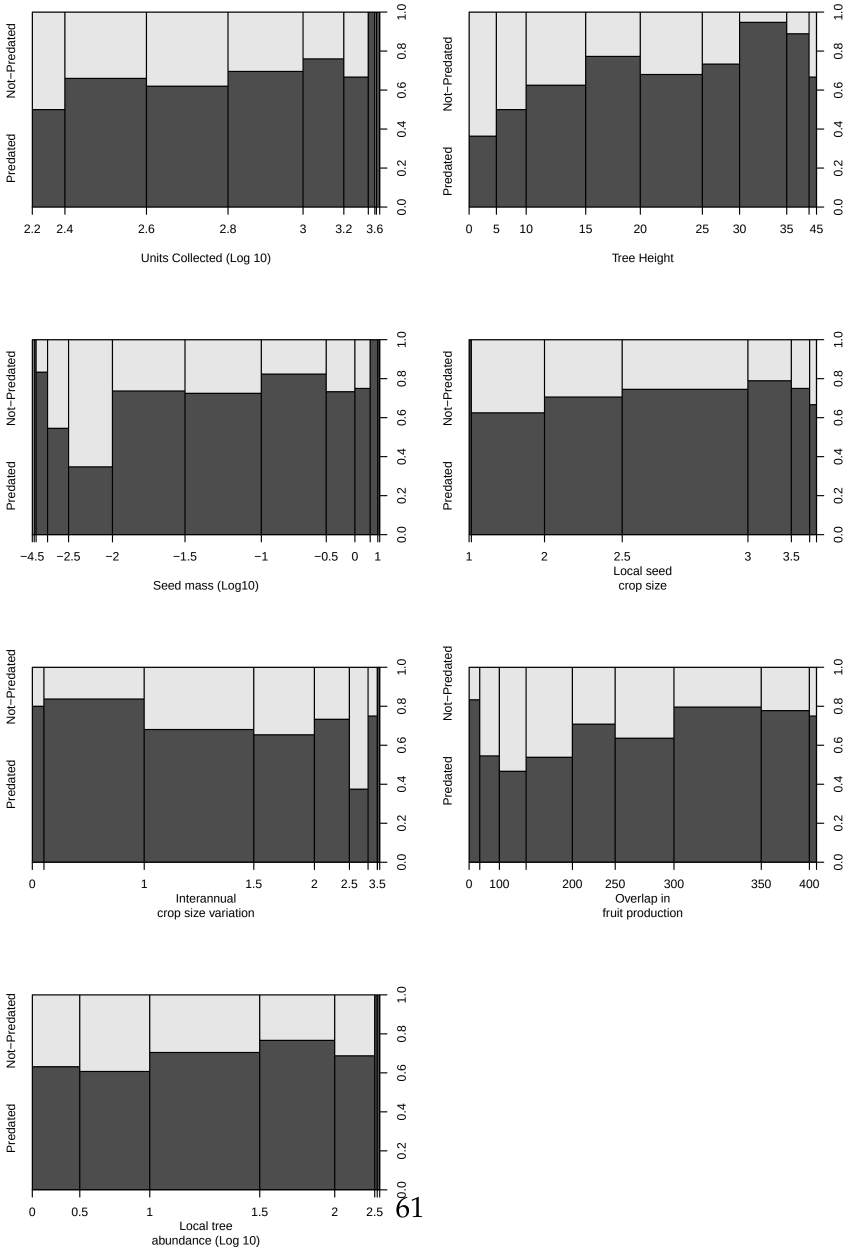

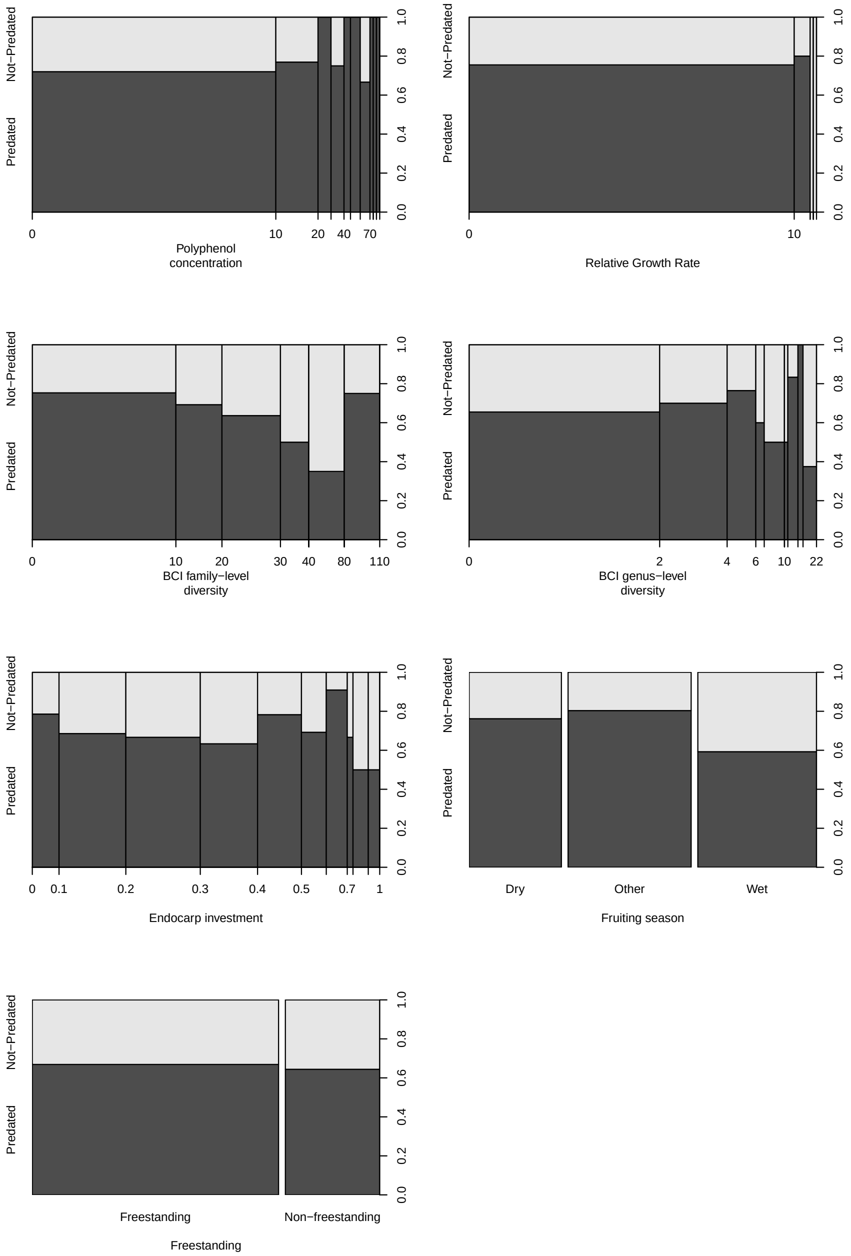
Not−Predated
Predated
0
10
20
40
70
Polyphenol concentration
Relative Growth Rate
BCI family−level diversity
0
10
20
30
40
80
110
BCI genus−level diversity
0
2
4
6
10
22
Endocarp investment
0
0.1
0.2
0.3
0.4
0.5
0.7
1
Fruiting season
Dry
Other
Wet
Freestanding
Non−freestanding
0.0
0.2
0.4
0.6
0.8
1.0

12 Figure S4: Quantitative food webs for Coleoptera and Lepidoptera

Figure S4. Quantitative food webs showing interactions between plants and their a) coleopteran and b) (overleaf) lepidopteran seed predators. For interpretation of the information in the food webs, see legend of Figure 4 in the main text.

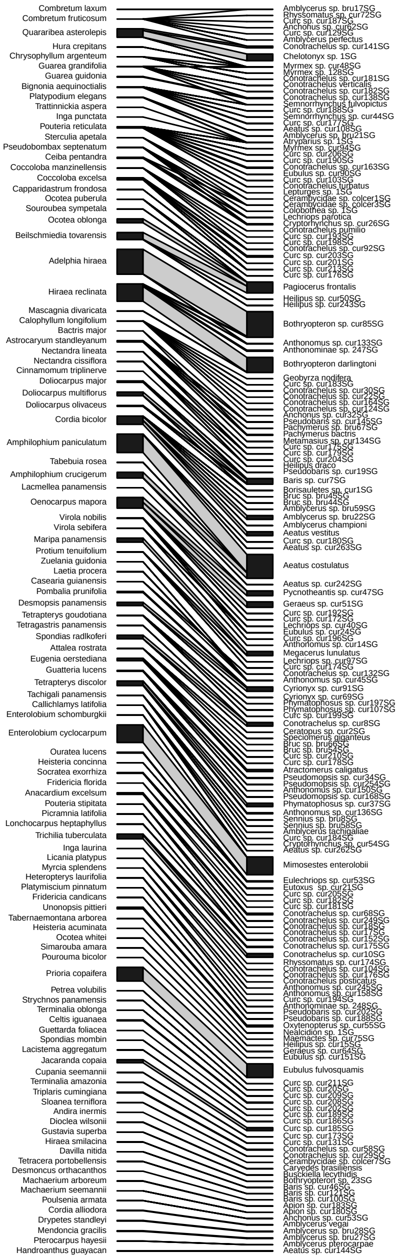

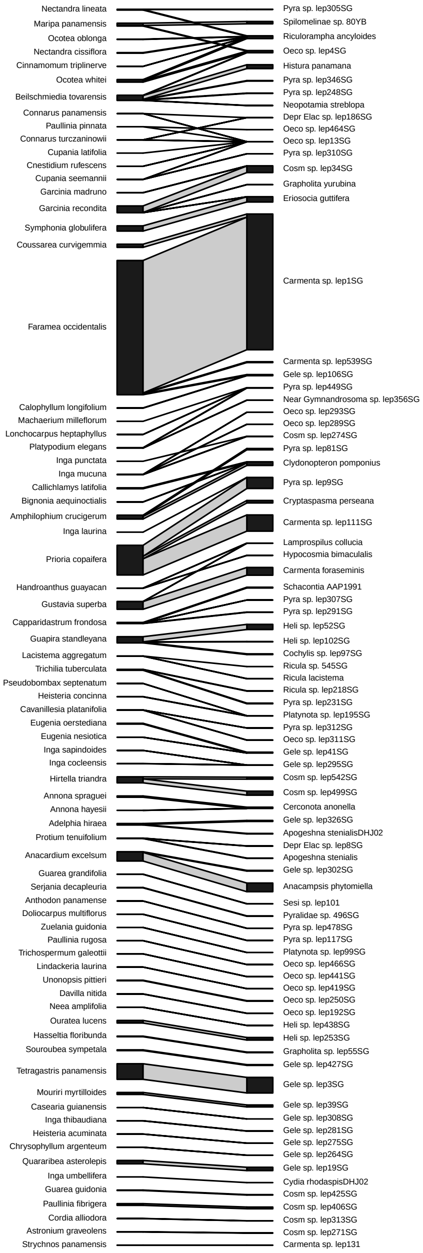
Nectandra lineata
Maripa panamensis
Ocotea oblonga
Nectandra cissiflora
Cinnamomum triplinerve
Ocotea whitei
Beilschmiedia tovarensis
Connarus panamensis
Paullinia pinnata
Connarus turczaninowii
Cupania latifolia
Cnestidium rufescens
Cupania seemannii
Garcinia madruno
Garcinia recondita
Symphonia globulifera
Coussarea curvigemmia
Faramea occidentalis
Calophyllum longifolium
Machaerium milleflorum
Lonchocarpus heptaphyllus
Platypodium elegans
Inga punctata
Inga mucuna
Callichlamys latifolia
Bignonia aequinoctialis
Amphilophium crucigerum
Inga laurina
Prioria copaifera
Handroanthus guayacan
Gustavia superba
Capparidastrum frondosa
Guapira standleyana
Lacistema aggregatum
Trichilia tuberculata
Pseudobombax septenatum
Heisteria concinna
Cavanillesia platanifolia
Eugenia oerstediana
Eugenia nesiotica
Inga sapindoides
Inga cocleensis
Hirtella triandra
Annona spraguei
Annona hayesii
Adelphia hiraea
Protium tenuifolium
Anacardium excelsum
Guarea grandifolia
Serjania decapleuria
Anthodon panamense
Doliocarpus multiflorus
Zuelania guidonia
Paullinia rugosa
Trichospermum galeottii
Lindackeria laurina
Unonopsis pittieri
Davilla nitida
Neea amplifolia
Ouratea lucens
Hasseltia floribunda
Souroubea sympetala
Tetragastris panamensis
Mouriri myrtilloides
Casearia guianensis
Inga thibaudiana
Heisteria acuminata
Chrysophyllum argenteum
Quararibea asterolepis
Inga umbellifera
Guarea guidonia
Paullinia fibrigera
Cordia alliodora
Astronium graveolens
Strychnos panamensis
Pyra sp. lep305SG
Spilomelinae sp. 80YB
Riculorampha ancyloides
Oeco sp. lep4SG
Histura panamana
Pyra sp. lep346SG
Pyra sp. lep248SG
Neopotamia streblopa
Depr Elac sp. lep186SG
Oeco sp. lep464SG
Oeco sp. lep13SG
Pyra sp. lep310SG
Cosm sp. lep34SG
Grapholita yurubina
Eriosocia guttifera
Carmenta sp. lep1SG
Carmenta sp. lep539SG
Gele sp. lep106SG
Pyra sp. lep449SG
Near Gymnandrosoma sp. lep356SG
Oeco sp. lep293SG
Oeco sp. lep289SG
Cosm sp. lep274SG
Pyra sp. lep81SG
Clydonopteron pomponius
Pyra sp. lep9SG
Cryptaspasma perseana
Carmenta sp. lep111SG
Lamprospilus collucia
Hypocosmia bimaculalis
Carmenta foraseminis
Schacontia AAP1991
Pyra sp. lep307SG
Pyra sp. lep291SG
Heli sp. lep52SG
Heli sp. lep102SG
Cochylis sp. lep97SG
Ricula sp. 545SG
Ricula lacistema
Ricula sp. lep218SG
Pyra sp. lep231SG
Platynota sp. lep195SG
Pyra sp. lep312SG
Oeco sp. lep311SG
Gele sp. lep41SG
Gele sp. lep295SG
Cosm sp. lep542SG
Cosm sp. lep499SG
Cerconota anonella
Gele sp. lep326SG
Apogeshna stenialisDHJ02
Depr Elac sp. lep8SG
Apogeshna stenialis
Gele sp. lep302SG
Anacampsis phytomiella
Sesi sp. lep101
Pyralidae sp. 496SG
Pyra sp. lep478SG
Pyra sp. lep117SG
Platynota sp. lep99SG
Oeco sp. lep466SG
Oeco sp. lep441SG
Oeco sp. lep419SG
Oeco sp. lep250SG
Oeco sp. lep192SG
Heli sp. lep438SG
Heli sp. lep253SG
Grapholita sp. lep55SG
Gele sp. lep427SG
Gele sp. lep3SG
Gele sp. lep39SG
Gele sp. lep308SG
Gele sp. lep281SG
Gele sp. lep275SG
Gele sp. lep264SG
Gele sp. lep19SG
Cydia rhodaspisDHJ02
Cosm sp. lep425SG
Cosm sp. lep406SG
Cosm sp. lep313SG
Cosm sp. lep271SG
Carmenta sp. lep131

13 Figure S5: Quantitative food web excluding singleton observations

Figure S5. Quantitative food web showing the interactions between seeds and their internally feeding seed predators. For interpretation, see legend of Figure 4 in the main text. In this version of the food web, singleton interactions (i.e. plant-seed predator interactions observed only once) have been excluded.

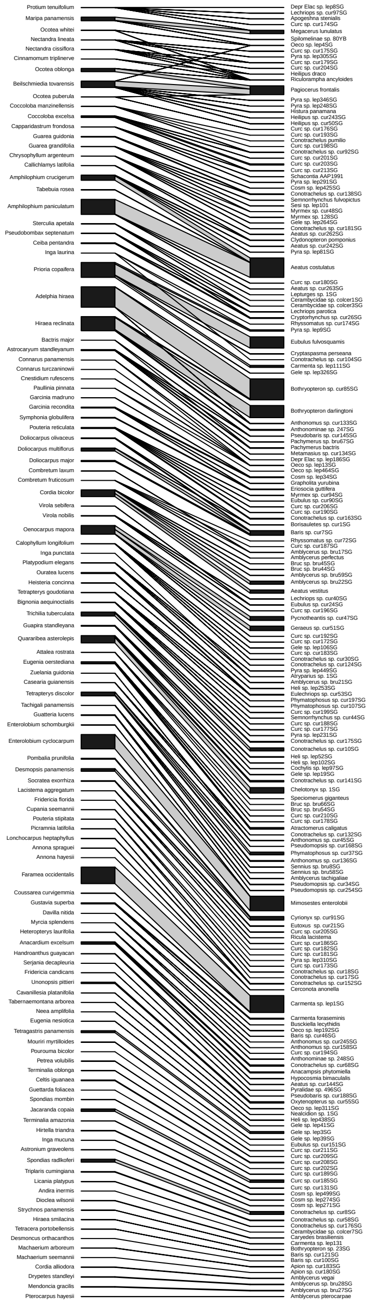

14 Figure S6: Relationship between potential for apparent competition and phylogenetic distance

Figure S6. Potential for indirect interactions (as assessed using the PAC index; see main text) plotted against the phylogenetic distance between pairs of plant species. Shown are only cases where PAC¿0. The red line is a trend line obtained from a linear model, and used for the purposes of plotting only (to visualise patterns in the relationship between PAC and pairwise phylogenetic distances).

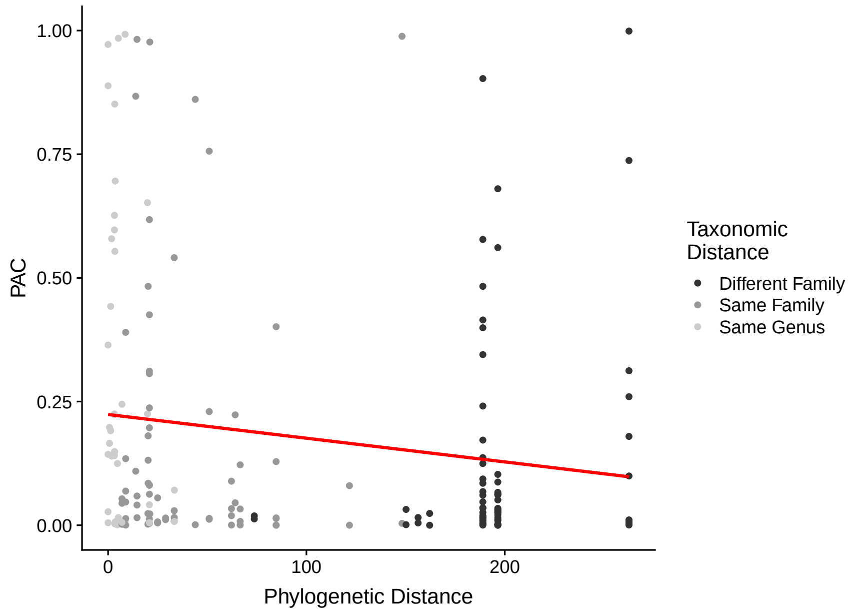

Supplement: Supplementary file 1 [file ELE-22-1638-s001.pdf]
